# Factors associated with the Single Leg Squat test in female soccer players: a cross-sectional study

**DOI:** 10.1186/s13102-024-00853-1

**Published:** 2024-04-02

**Authors:** John Ressman, Philip von Rosen, Wilhelmus Johannes Andreas Grooten, Eva Rasmussen-Barr

**Affiliations:** 1 Department of Neurobiology, Care Sciences and Society, Division of Physiotherapy, Karolinska Institutet, Alfred Nobels Allé 23, Huddinge, Stockholm, 141 83 Sweden; 2 Medical Unit Occupational Therapy and Physiotherapy, Allied Health Professionals, Karolinska University Hospital, Stockholm, 141 86 Sweden

**Keywords:** Visual assessment, Movement quality, Functional tests, Hip strength, Ankle dorsiflexion, Psychosocial factors, Rehabilitation

## Abstract

**Background:**

The Single Leg Squat (SLS) test is widely used in the clinical setting to examine and evaluate rehabilitation goals. It is simple to perform and is proposed to have biomechanical and neuromuscular similarities to athletic movements. The aim of the present study was to investigate whether demographics, previous injuries, and biomechanical and psychosocial factors are associated with the outcome of the SLS, assessed as a total score for all segments and as a separate knee segment in elite and sub-elite female soccer players.

**Methods:**

We conducted a cross-sectional study involving 254 female soccer players (22 yrs; SD ± 4, height 1.69 m; SD ± 0.1, weight 64 kg; SD ± 6) from divisions 1–3 of the Swedish Soccer League. During the preseason, we assessed the participants using the SLS and tested their hip strength and ankle mobility. Demographics, previous injury, sleep quality, fear of movement, anxiety, and perceived stress were assessed with questionnaires. Logistic regression models were built to analyse the association between the outcome of the SLS and the independent variables for the dominant and non-dominant leg.

**Results:**

Significantly more participants failed the SLS on the dominant leg compared with the non-dominant leg (*p* < 0.001). The outcome of the SLS associated with various biopsychosocial factors depending on if the dominant or non-dominant leg was tested. The total score associated with hip strength for the dominant (OR 0.99, 95% CI 0.98–0.99, *p* = 0.04) and the non-dominant leg (OR 0.99, 95% CI 0.97–0.99, *p* = 0.03). The knee segment associated with division level for the dominant (div 2; OR 2.34, 95% CI 1.01–5.12, *p* = 0.033. div 3; OR 3.07, 95% CI 1.61–5.85, *p* = 0.001) and non-dominant leg (div 2; OR 3.30, 95% CI 1.33-8.00, *p* = 0.01. div 3; OR 3.05, 95% CI 1.44–6.43, *p* = 0.003).

**Conclusions:**

This study identified that leg dominance, division level, hip strength, and psychosocial factors were associated with the outcome of the SLS when assessed as a total score and as a separate knee segment. This indicates that clinicians need to understand that movement control is associated with factors from several domains. Whether these factors and, the results of the SLS are related to injury need to be studied prospectively.

**Trial registration:**

Clinical Trials Gov, date of registration 2022-03-01. Clinical trials identifier: NCT05289284A.

**Supplementary Information:**

The online version contains supplementary material available at 10.1186/s13102-024-00853-1.

## Background

Injuries in female soccer players are common, where they are reported to have a 2–3 times higher risk of receiving an anterior cruciate ligament (ACL) injury compared to male players [1, 2]. In a recent meta-analysis, the pooled injury incidence rate (IIR) for a time-loss injury in female elite club players was reported to be 5.63/1000 hours for both training and match scenarios (overall) [1]. For amateur players, IIR is not frequently studied, but higher overall IIR is reported among amateur compared to elite players [1, 3, 4]. To better understand the cause of injury, and to prevent them, comprehensive biopsychosocial models have been proposed in which biomechanical as well as psychosocial risk factors are included [5, 6]. Dynamic knee valgus is a risk factor that has been associated with injuries in the lower extremity [7–10], and research has shown that this might be related to decreased ankle dorsiflexion [11, 12], decreased hip strength [13, 14] and neuromuscular recruitment [15–17].

Visual assessment of movement quality is commonly used in the clinical setting when examining an injured athlete or evaluating rehabilitation goals. Furthermore, injury prevention programmes for athletes emphasise movement quality such as knee control, neuromuscular control, soft landings, and leg alignment [18, 19]. Single screening tests or combined test batteries are used to evaluate movement quality by observing “compensatory movements” and are often used as a preseason screening tool to prevent injuries in athletes [20–23]. The Single Leg Squat (SLS) test is one such test that is widely used in sports medicine and included in various functional screening batteries [24–26]. The test is simple to perform and is proposed to have biomechanical and neuromuscular similarities to athletic movements, as it simulates common movements pattern such as cutting and landing [27, 28]. Unfortunately, there is no uniform SLS described in the literature, and different performance and assessment protocols are presented [29]. Some authors propose a uni-segmental approach [30] assessing just one joint or body segment (e.g., a knee joint or the position of the trunk), whereas others propose a multi-segmental approach, assessing multiple joints and/or body segments at the same time (foot, ankle joint, knee joint, hip joint, pelvis, and trunk) [13]. The latter is sometimes presented as a total score for all segments [31, 32]. The SLS, including the Forward Stepdown (FSD) and the Lateral Step Down (LSD), has been reported to have moderate reliability across all its variation and is proposed to be feasible and reliable in a clinical setting [29]. Furthermore, the SLS shows good validity in detecting abnormal kinematics in the lower extremity and trunk [25, 26, 33, 34]. However, when using both screening batteries [35] and different single functional tests, including the SLS [25, 36, 37], there is inconsistent evidence that poor movement quality is associated with an increased risk of injury in the lower extremity.

The wide clinical use of a visual assessment of the SLS highlights the need for further investigation to better understand what factors can explain the outcome of the SLS, both as a total score for all segments and for each separate segment. Given that several prevention programmes emphasise knee control, and that knee injuries are more common among female soccer players [2, 38, 39], information about the knee segment of the SLS is of great interest. A better understanding of the SLS and its association with demographics, previous injuries, and biomechanical and psychosocial factors might aid in rehabilitation and return-to-sport decisions, as well as guide safe and effective exercise prescriptions. To our knowledge, no previous study has investigated the association that both biomechanical and psychosocial factors have with the SLS. Thus, the aim of the present study was to investigate whether demographics, previous injuries, and biomechanical and psychosocial factors are associated with the outcome of the SLS, assessed as a total score for all segments and as a separate knee segment in elite and sub-elite female soccer players.

## Method

### Study design and participants

The present cross-sectional study is part of a larger prospective project investigating the predictive value of the SLS, as well as any associated risk factors, for acute and overuse injuries in female soccer players. The prospective project is registered at the United States National Library of Medicine, Clinical Trials Gov [40] 2022-03-01, and has the clinical trials identifier: NCT05289284A. The data collection for the present study was performed before the registration of the prospective project. A consecutive sample of twenty female soccer teams from the three highest division levels in the Swedish Soccer League was invited to participate via email, and those who accepted the invitation were screened for demographics, previous injuries, and biomechanical and psychosocial factors. Inclusion criteria were players 16 years or older who understood written and spoken Swedish and were contracted for the 2022 season with a team from one of the three top divisions. Exclusion criteria were two-footed players, players suffering from an ongoing injury that made it impossible to perform the physical tests without pain, and those who considered participation an additional risk for injury. Accordingly, 15 players were excluded as they were two-footed (*n* = 10), did not understand written or spoken Swedish (*n* = 2), were younger than 16 years old (*n* = 2), or had an ongoing injury (*n* = 1). Written informed consent was obtained for all participants, and the study was approved by the Regional Ethical Review Board in Stockholm: Ethical approval Dnr 2021–03067 with amendment Dnr 2021-05398-02.

### Data collection

After a series of pilot tests, all players were screened from January to February 2022 by one examiner (JR) with 25 years of experience as a physiotherapist in sports medicine. The participants completed a paper questionnaire on previous injuries and demographic factors (see Additional file 1) and performed three biomechanical tests (SLS, hip strength, and ankle dorsiflexion). This session took place at their local club. A web-based survey (SurveyMonkey©) was furthermore used to collect data from the participants on sleep quality, anxiety, perceived stress, and fear of avoidance (see Additional file 2). The web-based survey was sent to the participants the same day as the data collection at the local club and followed up the day after the test occasion, in cases of non-response.

The test leader (JR) started each test occasion at the player’s local club with a brief presentation about the upcoming session, which included information about how to handle ongoing injuries and pain during the tests. The participants were instructed to inform the test leader about possible ongoing injuries only if they experienced that the injuries would hamper their participation. Furthermore, they were informed that if they experienced pain during the test, a maximum limit of 3–4 on the Visual Analog Scale was acceptable [41, 42]. After receiving this information, the players answered the questionnaire on previous injuries and demographics.

### Questionnaires and biomechanical tests

#### Demographics and previous injuries

Information about the participant’s age, height, weight, soccer division, and leg dominance were collected as demographic data. The dominant leg was defined as the preferred kicking leg [43, 44], while the other leg was defined as the non-dominant leg. Each participant was able to register three kinds of injuries located in the head, lower belly, lower back, pelvis or lower extremities. Data on all injuries were collected with a pain manikin showing the exact location of an injury (see Additional file 1). *A time-loss injury* was defined as an injury that caused time-loss from training and competition that occurred any time during the 2022 season and/or the four weeks prior to the test occasion. The time-loss injuries were divided into 1–7 days, 8–28 days, or more than 28 days [45]. A severe injury was defined as at least one time-loss injury during the 2021 season, or earlier, that lasted three months or more, whereas *an injury problem* was defined as an injury that did not demand any time-loss from training and competition during the four weeks before the test occasion. The concept of injury problem, and the questions asked regarding this, were modified from the Oslo Sport Trauma Research Centre Overuse Injury Questionnaire, which is particularly designed to capture overuse injuries [46, 47].

### Biomechanical tests

#### The SLS

A multi-segmental SLS was used to assess the movement quality of the lower extremity and trunk [48]. The test was performed by standing on one leg with the arms folded across the chest, the non-weight bearing leg flexed so the foot was pointing backwards and the knee pointing straight down to the floor. The weight-bearing leg was positioned along a sagittal placed sticky tape on the floor, so that the toes pointed straight ahead, and the inside of the foot was parallel to the sticky tape. If the participants could not accomplish this, the foot could be placed in a way that felt comfortable. The participants were instructed to squat down in a controlled manner as deep as possible without lifting the heel from the ground or overly flexing the upper body (the test is described in detail elsewhere, see [48]). The original rating criteria [48] for the test are presented in Table 1 (no changes have been made, the article is licensed under a Creative Commons Attributions 4.0 International License, https://creativecommons.org/licenses/by/4.0/). In short, the test leader assessed the movement deviation from the vertical alignment of the four body segments (foot, knee, pelvis, and trunk) during three consecutive squats. In this context, the knee joint is defined as a segment due to the assessment of its position in the room/space. For the present study, the performance was dichotomously assessed for each separate segment, but the total score of zero to four points described in the original study (see Table 1 under movement deviation^a^) was changed to a pass/fail score (0/1 points) for all segments, that is, a total score of zero points if the subject passed the test (no fail in any segments), or one point if the subject failed the test, regardless of whether the subject failed in one or four segments. All SLS started with the left leg. The SLS has previously been reported to have a “moderate” interrater reliability and an “almost perfect” intrarater reliability for an active population [48].

**Table 1:** The original rating criteria of the SLS

Observed segment	Correct movement (pass = 0 point)	Movement deviation^a^ (fail = 1 point)
**Foot** ^**b**^ The relationship of the sagittal plane and metatarsale 2.	Os metatarsale 2 is in relation to the sagittal plane placed in a lateral angle of ≤ 10°.	The metatarsale 2 is in relation to the sagittal plane placed in a lateral angle that **clearly exceeds 10°.**
**Knee** Position of the knee in relation to the foot. Medial/lateral perturbation of the knee.	The centre of the knee is well aligned over the centre of the foot. The movement of the knee is vertical and smooth without any medial/lateral shake.	The centre of the knee is **clearly** over or medial to digitorum 1. The movement is jerky and **repeated** medial/lateral shake of the knee is seen.
**Pelvis** Lateral pelvis shift and/or pelvis rotation.	No lateral pelvis shift and/or pelvis rotation are seen.	The pelvis is **clearly** shifted lateral and/or rotated in any direction.
**Trunk** Centre of mass: trunk lean, perturbation and balance.	The trunk is well aligned over the pelvis, hip, knee and foot.	The trunk **clearly** leans in either direction, there is **obvious** trunk sway, loss of balance or movement of the arms.

^a^A movement deviation for a segment (1 point) can only be registered one time during the three squats, i.e., a total score of 0–4 points is possible

^b^The position of the foot should be observed before the test is executed. If the test person cannot place the foot in the correct position, they are allowed to put the feet where they feel comfortable.

The rater is only allowed to correct the tested person if they:

1. Flex the upper body as much as the hip, pelvis and groin cannot be observed.

2. If the heel is lifted from the ground and/or if the foot is moved from its starting position.

3. If the test person does not understand the instructions and performs a pistol squat instead of the SLS

#### Ankle dorsiflexion

For the measurement of ankle dorsiflexion (ADF), the weight-bearing lunge test that calculates the ADF by using a simple trigonometric function was used. The performance and method for calculation are described in detail elsewhere [49, 50]. Following the original protocol, the subjects stood in a weight-bearing lunge position, facing a wall with two 70-centimetre tape measures fixed perpendicularly to each other on the ground and the wall. From this position, the subjects were asked to increase the distance from the wall by moving the foot further back until their maximal distance was reached. The non-tested foot was instructed to be placed behind them in a standardised “fencing position.” This position for the non-tested leg differs from the protocol [49–51] since our prior pilot testing showed that the subjects could increase their range of ADF by not having a standardised position. In previous articles [49, 50], a mean value of three attempts was used. For this study, only the maximum distance was recorded (after three attempts) due to the practical time frame for screening a soccer team. In previous studies, the test has shown an “almost perfect” intrarater reliability, a standard error of measurement of 0.6°-1.2° and a minimal detectable change of 1.7°-3.3° [49, 50].

#### Hip strength

**Fig. 1:**
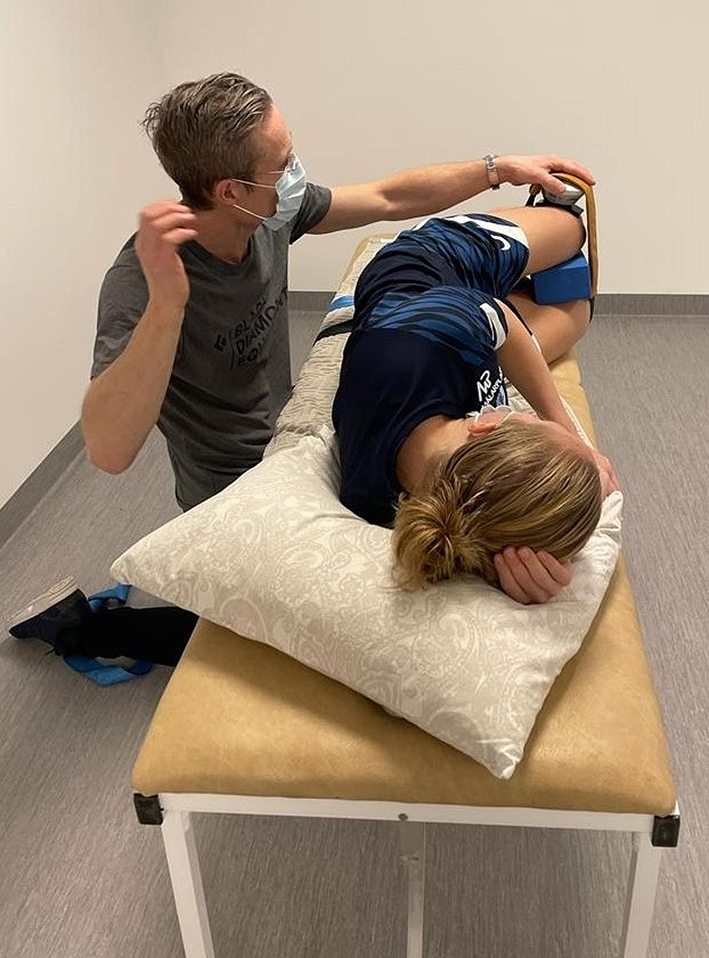
The set-up when testing hip strength with the Clamshell test

The combined hip abductor and external rotation strength was measured with a handheld dynamometer (MicroFET2TM wireless, Hoggan Scientific, LLC. USA) with the player performing an isometric clamshell (CLAM) test as described previously [52]. The subjects were placed on their sides, lying on an examination table, with the hips in a 45° flexion and 0° abduction/adduction, and the knees in a 90° flexion, while the hand-held dynamometer was fixated just proximal to the lateral epicondyle of the upper knee, see Fig. 1. Before fixation, the distance between the proximal greater trochanter and the proximal lateral epicondyle of the femur was measured with a measuring tape. Three maximal efforts were made with a 5-second duration and 15–30 s of rest in between, and a mean value for the efforts was calculated. The hand-held dynamometer values were measured in Newtons (N), and these were multiplied by the length of the femur (m) to calculate maximal peak torque values (Nm). Torque was then body size-normalised by the subject’s weight (kg) and height (m) [53, 54] and multiplied by 100 (Nm/(kg*m) *100). The CLAM test has an “almost perfect” test-retest reliability (ICC = 0.97, 95% CI 0.94–0.99) and good validity (Pearson’s correlation coefficient = 0.84) [52].

### Psychosocial questionnaires

#### Perceived stress scale − 14 items

General perceived stress was measured using the Swedish version of the Perceived Stress Scale − 14 items **(**PSS-14) [55], which has shown satisfactory psychometric properties [55]. The PSS-14 contains 14 items, and the total score ranges from 0 to 56, where 56 represents high stress [55, 56]. PSS-14 was originally developed by Cohen et al. [56].

#### Pittsburgh Sleep Quality Index

Sleep quality was measured with the Pittsburgh Sleep Quality Index (PSQI) [57]. It aims to measure sleep in different dimensions, but most fundamentally, it can be used as a simple screening measure to identify good and poor sleepers [57, 58]. The PSQI contains 19 items and a total global score that ranges from 0 to 21, where 21 represents poor sleep [57, 58]. The cut-off for poor sleepers has been set to six points, with a sensitivity of 89.6%, a specificity of 86.5% [58], and an area under the curve of 0.999 [57]. A Swedish unpublished translation of the PSQI exists, which has been used in clinical settings, and medical research and development.

#### Generalized anxiety Disorder-7 items

Anxiety was measured with the Generalized Anxiety Disorder-7 items (GAD-7) scale, which has shown good psychometric properties [59]. It contains seven items and ranges from 0 to 21, where 21 represents high anxiety [59]. Cut-off points of 5, 10, and 15 have been interpreted as mild, moderate, and severe levels of anxiety, respectively [59]. The Swedish version was used [60].

#### Athletic fear Avoidance Questionnaire

Fear of avoidance was measured with the Athletic Fear Avoidance Questionnaire (AFAQ).

This questionnaire scale measures sport-injury-related fear avoidance in athletes and could be used to identify potential psychological barriers, for example, to rehabilitation [61]. It contains 10 items and ranges from 10 to 50, where 50 represents a high fear of avoidance. Overall, the AFAQ has shown good internal and external validity [61], and for the present study, a Swedish version was used, and a pilot study found adequate test-retest reliability (ICC_2.1 =_ 0.74, unpublished data).

### Statistical analysis

The demographic data were checked for normality by comparison of means and medians, visual analyses of histograms and distributional diagnostic plots, as well as tested for skewness and kurtosis [62]. As not all demographic data were normally distributed, descriptive data were calculated and expressed as medians, minimum/maximum values, absolute numbers, and percentages. Before any calculation, data concerning the left or right leg were categorised as dominant and non-dominant leg. Regarding the outcome of the SLS, McNemar´s test was used to analyse the statistical difference between the dominant and non-dominant leg. For the difference in hip strength and ankle dorsiflexion between the dominant and non-dominant leg, the Wilcoxon signed rank test for paired non-normally distributed data was used, and for the difference in hip strength and ankle dorsiflexion within the dominant and non-dominant leg, the Wilcoxon rank sum test for unpaired non-normally distributed data was used.

The dependent variable of the present study was the SLS, and its outcome was pass or fail. For the dominant and non-dominant leg, two dependent variables were used in the statistical analysis: (1) the total score (pass/fail) of all segments and, (2) the pass/fail score for the separate knee segment. The choice of independent variables was based on clinical experience and previous research [63]. The continuous variables used in the study to build the regression models were ADF, hip strength, AFAQ, and PSS-14, while the categorical variables comprised age (16–19 yrs., 20–24 yrs., and 25–39 yrs.), soccer division (1–3), severe injury (yes/no), time-loss injury season 2021 (yes/no), time-loss injury (yes/no), injury problem (yes/no), PSQI (≤ 5 good sleepers/ ≥6 poor sleepers), and GAD-7 (no anxiety, ≥ 5 mild anxiety/≥10 moderate/severe anxiety).

As a first step, all independent variables were analysed one by one in a single univariate logistic regression. Separate models were then constructed for the total score and knee segment for both the dominant and non-dominant leg. A backward logistic regression analysis was used for the multivariate analyses that specified the significance level for the removal of eligible independent variables from the model at *p* ≥ 0.20. The results were expressed as an odds ratio (OR) with a 95% confidence interval (95% CI). A stepwise logistic regression model might be justified when investigating a relatively new outcome, and when the importance of the covariates (independent variables) and their association with the outcome is not well understood [63, 64]. Stepwise regression might then be a fast and effective way to screen a large number of covariates [64]. Therefore, a stepwise logistic regression was chosen in the present study, as the association with most of the independent variables is unknown or has not previously been studied. It is recommended in multiple regression models that for every variable screened for association, there are at least ten events [65]. However, this rule of thumb should not be applied categorically, as other factors could affect the stability of a model [65]. Moreover, there is in some cases evidence supporting the reduction of this rule to 5–9 events [66]. The final multivariate models were tested for adequacy by the Hosmer-Lemeshow Goodness of Fit test [67, 68] and by the linktest procedure in the statistical software program STATA *15.1.* The remaining variables were also tested for possible interactions. To ascertain that the basic assumptions for conducting logistic regression were met, data were checked for numerical limits, linearity of the log odds, multicollinearity, sample size, data independence, homogeneity, and outlying and influential points [63, 64, 69, 70]. All statistical analyses were performed using STATA version 15.1, and Microsoft Office Excel version 16 for Windows 10 was used to collect and organise the data before importing it to STATA. As the aim of the present study was to find associations with the outcome of the SLS; no adjustment for multiple comparisons (e.g., Bonferroni) was made as such an approach may inflate the risk of type II errors, which makes it more difficult to identify associations [71]. Throughout all calculations, the significance level was set to *p* ≤ 0.05. The code for all statistical analysis in STATA is included in Additional file 3.

## Results

### Participants

Altogether, a total of 254 players from soccer divisions 1–3 in Sweden were included in the study. Demographics, any previous severe injuries, and biomechanical and psychosocial factors stratified by division are described in Table 2.

**Table 2:** Subject characteristics for the total group, stratified by division 1–3

**Characteristics**	Total group	Division 1	Division 2	Division 3
	**(n = 254)**	**(n = 89)**	(n = 51)	(n = 114)
**Age, yr.**				
Mdn (min-max)	22 (16–39)	23 (17–38)	23 (16–31)	19 (16–39)
**Height, m**				
Mdn (min-max)	1.70 (1.52–1.83)	1.71 (1.57–1.82)	1.68 (1.52–1.83)	1.69 (1.55–1.83)
**Weight, kg**				
Mdn (min-max)	63 (50–85)	64 (55–85)	62.5 (50–78)	63 (50–83)
**Ankle dorsiflexion**				
Mdn (min-max)				
Dominant leg	45° (32°-56°)	44° (32°-54°)	45° (32°-55°)	45° (36°-56°)
Non-dominant leg	45° (34°-58°)	46° (35°-53°)	44° (34°-53°)	45° (36°-58°)
**Hip strength**				
Mdn (min-max)				
Dominant leg	96* (48–196)	93 (60–172)	101 (62–196)	96 (48–144)
Non-dominant leg	98* (40–204)	97 (40–160)	102 (63–204)	97 (55–149)
**AFAQ** ^**a**^				
Mdn (min-max)	23 (10–45)	23 (10–42)	25 (10–42)	21 (20–45)
**PSS-14** ^**b**^				
Mdn (min-max)	32 (20–42)	31 (22–42)	33 (22–41)	32 (20–39)
**PSQI** ^**c**^				
Mdn (min-max)	5 (0–15)	4 (0–15)	4 (0–15)	5 (1–14)
**GAD-7** ^**d**^				
Mdn (min-max)	5 (0–20)	4 (0–20)	6 (0–16)	6 (0–17)
**Severe ** **injuries DL**				
^e^Knee injuries, n (%)	40 (51%)	9 (39%)	16 (80%)	15 (43%)
^f^Other injuries, n (%)	38 (49%)	14 (61%)	4 (20%)	20 (47%)
**Severe ** **injuries NDL**				
^e^Knee injuries, n (%)	36 (58%)	15 (52%)	10 (91%)	11 (50%)
^f^Other injuries, n (%)	26 (42%)	14 (48%)	1 (9%)	11 (50%)

^*^Denotes statistically significant differences between groups, p-values at *p* ≤ 0.05;

^a^AFAQ: Athletic Fear Avoidance Questionnaire; ^b^PSS-14: Perceived Stress Scale 14-item; ^c^PSQI: Pittsburgh Sleep Quality Index; ^d^GAD-7: Generalized Anxiety Disorder 7-item scale; ^e^Knee injuries; contains fractures, ligament- and overuse injuries expressed in total numbers and percentage; ^f^Other injuries; contains all other injuries in the lower back and lower extremity except for the knee, expressed in total numbers and percentage.n: denotes the number of subjects in the total group and each division; Mdn: median; yr.=years; m = metres; kg = kilograms; DL = dominant leg; NDL = non-dominant leg

### Dominant versus non-dominant leg

The total number of cases (fail of the SLS) for the total score was 176 for the dominant leg (DL) compared to 117 cases for the non-dominant leg (NDL) (*p* < 0.001), and for the knee segment, 102 cases were found for the DL compared to 70 cases for the NDL (*p* < 0.001).

A significant difference was found between the DL and NDL regarding hip strength (*p* = 0.03) but not for ankle dorsiflexion (*p* = 0.11), see Table 2. There was a difference in hip strength between those who passed the SLS and those who failed the SLS for the total score and the knee segment. Within the NDL, the difference between those who passed and failed the SLS was significant for the total score (*p* = 0.02) and the knee segment (*p* = 0.01), but not for the DL (total score: *p* = 0.06, knee segment: 0.32). In the cohort, 231 players were right-footed, and 23 players were left-footed.

### The SLS for all segments, the total score

The univariate logistic regression analysis for the total score of all segments in the DL and NDL is reported in Table 3. For the DL, ankle dorsiflexion (ADF) and hip strength were significantly associated with a failure of the total score, and for the NDL four variables were significantly associated: soccer division, age, hip strength, and severe injury.

**Table 3:** Univariate analyses for the assessment of all segments during the SLS, the total score

Variable	**SLS test for all segments: non-dominant leg**					SLS test for all segments: dominant leg				
	n^a^	Cases (%) ^b^	**OR** ^**c**^	95% CI^d^	p-value^e^	n^a^	Cases (%) ^b^	**OR** ^**c**^	95% CI^d^	p-value^e^
**Division**	254	117 (46)	1.43	1.08–1.89	0.01*	254	**176 (69)**	1.25	0.93–1.6	0.14
Div. 1	89	32 (36)	1			89	56 (63)	1		
Div. 2	51	24 (47)	1.58	0.79–3.19		51	37 (73)	1.56	0.74–3.30	0.25
Div. 3	114	61 (54)	2.05	1.16–3.62	0.01*	114	83 (73)	1.58	0.87–2.86	0.13
**Age category**	254	115 (45)	0.65	0.47–0.91	0.01*	254	176 (69)	0.97	0.69–1.37	0.86
16–19 years	84	47 (56)	1			84	61 (73)	1		
20–24 years	99	45 (46)	0.66	0.37–1.18	0.16	99	64 (65)	0.69	0.37–1.30	0.25
25–39 years	71	25 (35)	0.43	0.22–0.82	0.01*	71	51 (72)	0.96	0.48–1.95	0.91
**Ankle dorsiflexion** ^**f**^	254	117 (46)	0.97	0.92–1.03	0.39	254	176 (69)	0.92	0.86–0.99	0.03*
**Hip strength** ^**g**^	253	117 (46)	0.99	0.98–0.99	0.04*	252	174 (69)	0.99	0.98–0.99	0.02*
**A severe injury** ^**h**^	247	114 (46)				247	170 (69)			
No	195	100 (51)	1			190	130 (68)	1		
Yes	52	14 (27)	0.35	0.18–0.69	0.00*	57	40 (70)	1.09	0.57–2.07	0.80
**A time-loss injury** ^**i**^	250	113 (45)				250	174 (70)			
No	161	76 (47)				135	88 (65)	1		
Yes	89	39 (44)	1	0.52–1.47	0.61	115	86 (75)	1.58	0.91–2.75	0.10
**An injury problem** ^**j**^	254	117 (46)	0.87			254	176 (69			
No	224	99 (44)	1			214	147 (69)	1		
Yes	30	18 (60)	1.89	0.87–4.11	0.11	40	29 (73)	1.20	0.57–2.55	0.63
**AFAQ** ^**g**k^	252	115 (46)	0.97	0.93-1.00	0.07	252	174 (69)	0.99	0.96–1.03	0.72
**PSS-14** ^**l**^	252	115 (46)	0.96	0.90–1.03	0.25	252	174 (69)	0.94	0.87–1.01	0.09
**PSQI** ^**m**^	252	115 (46)				252	174 (69)			
Good sleepers, ≤ 5 points	156	66 (42)	1			156	105 (68)	1		
Poor sleepers, ≥ 6 points	96	49 (51)	1.42	0.85–2.37	0.18	96	69 (71)	1.24	0.71–2.17	0.45
**GAD-7** ^**n**^	252	115 (46)	1.07	0.77–1.49	0.70	252	174 (69)	1.36	0.94–1.97	0.11
No anxiety	108	49 (45)	1			108	68 (63)	1		
Mild anxiety ≥ 5 points	98	43 (44)	0.94	0.54–1.63	0.83	98	72 (74)	163	0.90–2.95	0.11
Moderate/severe anxiety ≥ 10 points	46	23 (50)	1.20	0.60–2.40	0.60	46	34 (74)	1.67	0.78–2.51	0.19

^*^Denotes a statistically significant differences at *p* ≤ 0.05;

^a^n: total sample; ^b^Cases (%): total number of subjects failing on the SLS test, and in brackets, the risk/probability to fail the SLS test expressed in the percentage of the total number of performed Single Leg Squats; ^c^OR: odds ratio; ^d^95% CI: 95% confidence interval; ^e^p-value: probability value; ^f^Ankle dorsiflexion: measured with the Weight Bearing Dorsiflexion Lunge Test (WBLT) and calculated with a trigonometric dorsiflexion angle (TA); ^g^Hip strength: Side-Lying Clamshell (CLAM), body-sized independent measurement; Newton (N)*length of femur (m)/ (Body weight (kg)*height (m)) *100; ^h^A severe Injury: One or more time-loss injuries located in the head, lower belly, lower back, pelvis or lower extremities, during season 2021 or earlier, that lasted three months or more; ^i^A time-loss injury: One or more time-loss injuries located in the head, lower belly, lower back, pelvis or lower extremities during season 2021; ^j^An injury problem: An injury problem located in the head, lower belly, lower back, pelvis or lower extremities that did not demand any time-loss from game or training during the four weeks before or during the test occasion; ^k^AFAQ: Athletic Fear Avoidance Questionnaire; ^l^PSS-14: Perceived Stress Scale 14-item instrument; ^m^PSQI: Pittsburgh Sleep Quality Index; ^n^GAD-7: Generalized Anxiety Disorder 7-item scale

The multivariate models for the total scores are reported in Tables 4 and 5. The independent variables associated with the outcome of the SLS for the total score differed depending on which leg was tested, except for hip strength, which was associated with both the DL and the NDL (DL: OR 0.99, 95% CI 0.98–0.99, *p* = 0.04, NDL: OR 0.99, 95% CI 0.97–0.99, *p* = 0.03).

**Table 4:** Multivariate analysis of failing on the total score for the dominant leg during the SLS

	SLS test for all segments: dominant leg		
Variables	OR^a^	95% CI^b^	p-value^c^
**PSS-14** ^**d**^			
(No stress/stress; 0–56)	0.91	0.83–0.98	0.02*
**GAD-7** ^**e**^			
No anxiety	1		
Mild anxiety ≥ 5	1.83	0.96–3.50	0.07
Moderate/severe anxiety ≥ 10	2.21	0.96–5.07	0.06
**Ankle dorsiflexion** ^**f**^			
WBLT^f^ measured in degrees (TA^f^)	0.94	0.87–1.01	0.08
**Hip strength** ^**g**^			
CLAM^g^ measured in Nm/(kg*m) *100	0.99	0.98–0.99	0.04*

^*^Denotes statistically significant p-values at *p* ≤ 0.05

^a^OR: odds ratio; ^b^95% CI: 95% confidence interval; ^c^p-value: probability value; ^d^PSS-14: Perceived Stress Scale 14-item instrument; ^e^GAD-7: Generalized Anxiety Disorder 7-item scale; ^f^Ankle dorsiflexion: measured with the Weight Bearing Dorsiflexion Lunge Test (WBLT) and calculated with a trigonometric dorsiflexion angle (TA). ^g^Hip strength: Side-Lying Clamshell (CLAM)

**Table 5:** Multivariate analysis of failing on the total score for the non-dominant leg during the SLS

	SLS test for all segments: non-dominant leg		
Variables	OR^a^	95% CI^b^	p-value^c^
**Division**			
Div. 1	1		
Div. 2	1.79	0.85–3.79	0.13
Div. 3	1.94	1.06–3.57	0.03*
**A previous severe injury** ^**d**^			
No	1		
Yes	0.38	0.19–0.77	0.01*
**An injury problem** ^**e**^			
No	1		
Yes	2.28	0.98–5.31	0.06
**Hip strength** ^**f**^			
CLAM^e^ measured in Nm/(kg*m) *100	0.99	0.97–0.99	0.03*

^*^Denotes statistically significant p-values at *p* ≤ 0.05

^a^OR: odds ratio; ^b^95% CI: 95% confidence interval; ^c^p-value: probability value; ^d^A previous severe injury: One or more time-loss injuries during season 2021, or earlier, that lasted three months or more; ^e^An injury problem: An injury problem located in the head, lower belly, lower back, pelvis or lower extremities that did not demand any time-loss from game or training during the four weeks before or during the test occasion; ^f^Hip strength: Side-Lying Clamshell (CLAM)

### SLS for the knee segment

The univariate logistic regression analysis for the assessment of the knee segment in the DL and NDL is reported in Table 6. For the DL, four variables were significantly associated with a failure of the knee segment: soccer division, age, PSS-14, and GAD-7 if the subject belonged to the category of mild anxiety. For the NDL, five variables were significantly associated: soccer division, age, hip strength, an injury problem, and AFAQ.

**Table 6:** Univariate analyses for the assessment of the knee segment during the SLS

Variable	**SLS test for the knee segment: non-dominant leg**					SLS test for the knee segment: dominant leg				
	n^a^	Cases (%) ^b^	**OR** ^**c**^	95% CI^d^	p-value^e^	n^a^	Cases (%) ^b^	**OR** ^**c**^	95% CI^d^	p-value^e^
**Division**	254	**70 (28)**	1.70	1.22–2.37	0.00*	254	**102 (40)**	1.71	1.27–2.30	0.00*
Div. 1	89	14 (16)	1		0.06	89	22 (25)	1		
Div. 2	51	15 (29)	2.23	0.97–5.12	0.00*	51	23 (45)	2.5	1.20–5.20	0.01*
Div. 3	114	41 (36)	3.01	1.51–5.98	0.01*	114	83 (73)	3.05	1.66–5.58	0.00*
**Age category**	254	70 (28)	0.62	0.43–0.89	0.01*	254	102 (40)	0.75	0.54–1.03	0.08
16–19 years	84	34 (41)	1			84	43 (52)	1		
20–24 years	99	20 (20)	0.37	0.19–0.72	0.00*	99	32 (32)	0.46	0.25–0.83	0.01*
25–39 years	71	16 (23)	0.43	0.21–0.87	0.02*	71	27 (38)	0.59	0.31–1.11	0.10
**Ankle dorsiflexion** ^**f**^	254	70 (28)	1.0	0.93–1.06	0.89	254	102 (40)	0.95	0.89–1.01	0.11
**Hip strength** ^**g**^	253	70 (28)	0.98	0.97–0.99	0.00*	252	100 (40)	0.99	0.98–1.01	0.26
**A severe injury** ^**h**^	247	69 (28)				247	98 (40)			
No	195	60 (31)	1			190	76 (40)	1		
Yes	52	9 (17)	0.47	0.22–1.03	0.06	57	22 (39)	0.94	0.51–1.73	0.85
**A time-loss injury last 4 weeks** ^**i**^	250	69 (28)				250	100 (40)			
No	238	63 (27)	1			237	94 (40)	1		
Yes	12	6 (50)	2.78	0.86–8.93	0.09	13	6 (46)	1.30	0.43-4.0	0.64
**An injury problem** ^**j**^	254	70 (28)				254	102 (40)			
No	224	57 (26)	1			214	85 (40)	1		
Yes	30	13 (43)	2.24	1.03–4.90	0.04*	40	17 (43)	1.12	0.57–2.22	0.74
**AFAQ** ^**k**^	252	69 (27)	0.95	0.91–0.99	0.01*	252	101 (40)	0.99	0.95–1.02	0.41
**PSS-14** ^**l**^	252	69 (27)	0.99	0.91–1.06	0.69	252	101 (40)	0.93	0.87-1.00	0.05*
**PSQI** ^**m**^	252	69 (27)				252	101 (40)			
Good sleepers, ≤ 5 points	156	38 (24)	1			156	58 (37)	1		
Poor sleepers, ≥ 6 points	96	31 (32	1.48	0.84–2.60	0.17	96	43 (45)	1.37	0.82–2.30	0.23
**GAD-7** ^**n**^	252	69 (27)	0.90	0.61–1.31	0.57	252	101 (40)	1.16	0.82–1.62	0.40
No anxiety	108	29 (27)	1			108	37 (34)	1		
Mild anxiety ≥ 5 points	98	31 (32)	1.26	0.69–2.30	0.45	89	47 (48)	1.77	1.01–3.10	0.05*
Moderate/severe anxiety ≥ 10 points	46	9 (20)	0.66	0.29–1.54	0.34	46	17 (37)	1.13	0.55–2.31	0.75

^*^Denotes a statistically significant differences at *p* ≤ 0.05;

^a^n: total sample; ^b^Cases (%): total number of subjects failing the SLS test, and in brackets, the risk/probability to fail the SLS test expressed in the percentage of the total number of performed Single Leg Squats; ^c^OR: odds ratio; ^d^95% CI: 95% confidence interval; ^e^p-value: probability value; ^f^Ankle dorsiflexion: measured with the Weight Bearing Dorsiflexion Lunge Test (WBLT) and calculated with a trigonometric dorsiflexion angle (TA); ^g^Hip strength: Side-Lying Clamshell (CLAM), body-sized independent measurement; Newton (N)*length of femur (m)/ (Body weight (kg)*height (m)) *100; ^h^A severe injury: One or more time-loss injuries located in the head, lower belly, lower back, pelvis or lower extremities, during season 2021 or earlier, that lasted three months or more; ^i^A time-loss injury last 4 weeks: A time-loss injury located in the head, lower belly, lower back, pelvis or lower extremities four weeks before or during the test occasion, no pain allowed during the test occasion; ^j^An injury problem: An injury problem located in the head, lower belly, lower back, pelvis or lower extremities that did not demand any time-loss from game or training during the four weeks before or during the test occasion; ^k^AFAQ: Athletic Fear Avoidance Questionnaire; ^l^PSS-14: Perceived Stress Scale 14-item instrument; ^m^PSQI: Pittsburgh Sleep Quality Index; ^n^GAD-7: Generalized Anxiety Disorder 7-item scale

The multivariate models for the knee segment are reported in Tables 7 and 8. The independent variables associated with the outcome of the SLS for the knee segment differed depending on which leg was tested, except for division, which was associated with both the DL and NDL (DL: div 2; OR 2.34, 95% CI 1.01–5.12, *p* = 0.033. div 3; OR 3.07, 95% CI 1.61–5.85, *p* = 0.001.NDL: div 2; OR 3.30, 95% CI 1.33-8.00, *p* = 0.01. div 3; OR 3.05, 95% CI 1.44–6.43, *p* = 0.003).

**Table 7:** Multivariate analysis of failing on the knee segment for the dominant leg during the Single Leg Squat test

	SLS test for the knee segment: dominant leg		
Variables	OR^a^	95% CI^b^	p-value^c^
**PSS-14** ^**d**^			
(No stress/stress; 0–56)	0.90	0.83–0.98	0.01*
**GAD-7** ^**e**^			
No anxiety	1		
Mild anxiety ≥ 5	1.95	1.04–3.66	0.04*
Moderate/severe anxiety ≥ 10	1.52	0.68–3.39	**0.31**
**Ankle dorsiflexion** ^**f**^			
WBLT^f^ measured in degrees (TA^f^)	0.95	0.88–1.01	0.11
**Division**			
Div. 1	1		
Div. 2	2.34	1.01–5.12	0.03*
Div. 3	3.07	1.61–5.85	0.00*

^*^Denotes statistically significant p-values at *p* ≤ 0.05;

^a^OR: odds ratio; ^b^95% CI: 95% confidence interval; ^c^p-value: probability value; ^d^PSS-14: Perceived Stress Scale 14-item instrument; ^e^GAD-7: Generalized Anxiety Disorder 7-item scale; ^f^Ankle dorsiflexion: measured with the Weight Bearing Dorsiflexion Lunge Test (WBLT) and calculated with a trigonometric dorsiflexion angle (TA)

**Table 8:** Multivariate analysis of failing on the knee segment for the non-dominant leg during the Single Leg Squat test

	SLS test for the knee segment: non-dominant leg		
Variables	OR^a^	95% CI^b^	p-value^c^
**AFAQ** ^**d**^			
(No fear/fear; 10–50)	0.95	0.91–0.99	0.01*
**Hip strength** ^**e**^			
CLAM^e^ measured in Nm/(kg*m) *100	0.98	0.96–0.99	0.00*
**Division**			
Div. 1	1		
Div. 2	3.30	1.33-8.00	0.01*
Div. 3	3.05	1.44–6.43	0.00*
**An injury problem** ^**f**^			
No	1		
Yes	3.11	1.25–7.76	0.02*

^*^Denotes statistically significant p-values at *p* ≤ 0.05;

^a^OR: odds ratio; ^b^95% CI: 95% confidence interval; ^c^p-value: probability value; ^d^AFAQ: Athletic Fear Avoidance Questionnaire; ^e^Hip strength: Side-Lying Clamshell (CLAM); ^f^An injury problem: An injury problem located in the head, lower belly, lower back, pelvis or lower extremities that did not demand any time-loss from game or training during the four weeks before or during the test occasion

## Discussion

This study revealed various demographic, biomechanical and psychosocial factors associated with the outcome of the performance of the SLS, both for the total score and for the knee segment, and these factors differed between the dominant and the non-dominant leg. In a general perspective, and with small variations, the same independent variables turned out to be of importance in the multivariate models within the dominant and non-dominant leg, but they differed between the dominant and non-dominant leg. Regardless of whether the SLS was assessed as a total score for all segments, or as a separate knee segment, it was significantly more common to fail on the dominant leg than on the non-dominant leg. This has previously been reported in a cohort of 558 youth soccer players (boys and girls) aged 11–14 years [72]. The authors suggested that this was due to an imbalance in knee control between the legs, but urged caution when interpreting the results, as it could have been due to a learning effect. This is because the testing procedure for how they performed the SLS always started on the right leg, which for most of the players was the dominant leg. In the present study, most players reported that the right leg was their dominant leg, but in contrast to Räisänen et al. [72], the testing started with the left leg. It can be interpreted that the proposed bias due to a learning effect stated by Räisänen et al. [72] is questionable. However, the better SLS performance for the non-dominant leg shown in the present study, and that reported by Räisänen et al. [72], might not be surprising if the nature of the sport is considered. To perform repeated soccer drills in a unipedal stance will most likely modify proprioceptive factors, muscular control, and strength in the non-dominant leg [73–76].

For the non-dominant leg, players in the lowest division had increased odds of failing on the total score, compared to players from the highest division, but not for the dominant leg. Moreover, for the dominant and non-dominant leg, we found 2–3 times higher odds of failing on the knee segment for players in a lower division. It could be debated that the players in the higher division, who are more skilled players, also have a higher skill in controlling the weight-bearing leg on the soccer field, and therefore might be better in the performance of the SLS.

Regarding injuries, for the non-dominant leg, there were 2–3 times higher odds of failing on the SLS for the total score and the knee segment, respectively, if the player had an injury problem compared to no injury problem. Unexpectedly, the odds of failing on the SLS for the total score on the non-dominant leg was significantly lower for those with a previous severe injury. A possible explanation for this might be that 50% or more of the reported severe injuries were knee injuries (ligament injuries or fractures) that caused a time-loss of at least three months. These subjects most likely underwent rehabilitation where knee control and thus the SLS were integrated. Conversely, Whatman et al. [77] showed that individuals with a history of previous intra-articular knee injuries (3–11 years ago) did not have an increased likelihood of failing on a visually assessed SLS. They [77] discussed several reasons for their results and proposed that the time since the injury was an important factor.

Concerning hip strength, the non-dominant leg was significantly stronger compared to the dominant leg. The observed higher levels of hip strength for the non-dominant leg might not be surprising when considering the nature of the sport with repeated soccer drills in a unipedal stance. For the total score we found significantly lower odds of failing on the SLS for higher levels of hip strength on both legs; however, for the knee segment, this was only seen for the non-dominant leg. Overall, the results implicate that hip strength is of importance and associated with the outcome of the SLS. Consistent with our results, previous studies on the visual assessment of movement quality for the SLS, FSD, and LSD report conflicting results regarding associations with hip strength [13, 14, 31, 78]. Similar conflicting results have in addition been reported for kinematic studies on different single-leg tasks, including the SLS [11, 79, 80]. This might, however, be unsurprising considering the variety of test situations where isokinetic and isometric testing is used in different positions [16, 81–83] with different body-size normalisations [16, 83, 84]. Furthermore, it seems that the association between hip strength and knee valgus might be conditional on task demand [80] and that there is a gender difference in the performance of the SLS [17, 81, 84, 85]. The present study used the CLAM test to investigate hip strength, which is a combined external rotation and abduction strength test for the hip muscles. We used a method to calculate body-size independence that differs somewhat from other methods, thus in addition to weight, we also used height as a factor for body-size normalisation. This makes it difficult to compare our results with those of other studies.

Regarding ADF, we found non-significant lower odds of failing on the total score and the knee segment for higher levels of ADF for the dominant leg, but not for the non-dominant leg. Several studies on the visual assessment of the LSD have shown that poor performance is associated with reduced ADF [31, 78, 86, 87], while a study on the FSD [14] and one on the SLS [88] do not support this. Furthermore, quantitative studies using 3-dimensional analysis on both healthy subjects and those with a knee condition suggest that poor performance in the SLS, LSD, and FSD is associated with reduced ADF [11, 12, 89]. The reason for the non-significant association in the present study might be related to the relatively good ankle mobility displayed by this sample of female soccer players, as well as the lack of contrast in our data, as the range of data was small, which might have hampered the possibility of finding significant associations with the outcome of the SLS.

As far as we know, no previous research has investigated the association between the SLS and psychosocial factors. Three out of four investigated psychosocial factors were significantly associated with the models; only sleep quality was not associated with the SLS. Regarding the variable perceived stress, the outcome might be seen as odd, and we believe that it is not stress per se that explains the outcome of the SLS. Instead, stress might be related to other non-measured variables directly associated with the outcome. On the other hand, the decreased odds of failing the SLS due to higher levels of fear avoidance behaviour could be explained, as discussed above, by those subjects also previously having experienced a severe knee injury and most likely undergone rehabilitation where knee control and the SLS were integrated. Another possible explanation might be that subjects with a previous injury and/or higher levels of fear avoidance behaviour perform the SLS more carefully, with a greater chance to succeed. Fear of movement has previously been reported to be associated with a return to previous levels of activity in athletes and is therefore recommended to be taken into account during rehabilitation after ACL injuries [90, 91]. Our findings, i.e., that subjects with an increased level of anxiety (for the dominant leg) had higher odds of failing the SLS, might be of clinical interest if anxiety may increase due to the situation around the SLS assessment. Anxiety has been associated with performance problems in sports and other fields [92, 93], and is also reported as a psychological factor that negatively affects the return to play after an ACL injury [94]. The link between anxiety disorders and competitive performance is, however, not well understood, and it is unclear whether interventions that decrease anxiety are associated with better performance [93].

*All in all, f*rom a clinical perspective, this study contributes to an increased understanding of the SLS when assessed as a total score or as a separate knee segment. The clinician seemingly needs to consider leg dominance, division level and hip strength when using the SLS as a functional test among elite and sub-elite female soccer players. The results give implication for the clinician to further investigate these factors. Moreover, it seems to be of importance to address psychosocial factors in testing situations and focus on modifiable factors in rehabilitation. Furthermore, it seems also to be of importance to focus on leg dominance, rather than left and right leg, in the clinical context.

From the research perspective, it is of interest to further study the predictive value of the SLS in relation to these associated factors in a longitudinal design. The differences found in this study between the dominant leg and non-dominant leg indicate that data should be stratified, rather than adjusted, for leg dominance in the statistical analysis.

### Methodological considerations

The major strengths of this study are the inclusion of a specific sample of both elite and sub-elite female soccer players, the recruitment of a large number of players, and the inclusion of different associated factors. Furthermore, in our analyses, we used a multi-segmental SLS, which has been found to be reliable for use in an active population [48]. Our analyses of the four models were based on valid and reliable instruments for collecting the data and a robust statistical analysis. Nevertheless, we cannot rule out the risk that other unmeasured or confounding factors might have affected the results. There are, however, some limitations that need to be considered when interpreting the results. This study used a cross-sectional design, meaning that we cannot conclude a causal relationship between the dependent and independent variables. In addition, there was only one person (JR) who performed all physical tests, which could have rendered a systematic error in the assessment. The results might furthermore only be generalised to female soccer players of the same age and players at the same competition level (divisions 1–3). Finally, retrospective questions about previous injuries have been shown to have low recall accuracy [95, 96].

## Conclusion

This study identified a variety of different demographic, biomechanical and psychosocial factors, which associated with the outcome of the SLS for both the total score, assessed for all segments, and the separate knee segment. These factors differed between the dominant and non-dominant leg. The clinician seemingly needs to consider several factors when assessing the SLS among female soccer players, such as leg dominance, division level, hip strength, and psychosocial factors. These results might be of importance to consider in future prospective studies on the predictive value of the SLS for injury prevention in female soccer players.

### Electronic supplementary material

Below is the link to the electronic supplementary material.

Additional file 1

Additional file 2

Additional file 3

Additional file 4

## Data Availability

Data is not made open and available, since sensitive personal data could be traced to a living person which contrasts with our regulations § 6.3 in our Guidelines for research documentation and data management at Karolinska Institutet (DNR 1–20/2021), see Additional file 4. However, requests for data can be sent to the corresponding author and if there are no conflicts against the regulations, data could be made available in specific cases.

## Abbreviations

95% CI: 95% confidence interval
AFAQ: Athletic Fear Avoidance Questionnaire
ADF: Ankle dorsiflexion
ACL injury: Anterior cruciate ligament injury
CLAM: Clamshell
DL: Dominant leg
FSD: Forward Step Down
GAD-7: Generalized Anxiety Disorder-7 items
LSD: Lateral Step Down
N: Newton
Nm: Peak torque values
NDL: Non-dominant leg
OR: Odds ratio
PSS-14: Perceived Stress Scale-14 items
PSQI: Pittsburgh Sleep Quality Index
SLS: Single Leg Squat
