# Supplementary material for: Factors associated with the Single Leg Squat test in female soccer players: a cross-sectional study
Source: BMC Sports Sci Med Rehabil. 2024 Apr 2;16:76. doi: 10.1186/s13102-024-00853-1 (PMC10985895; doi:10.1186/s13102-024-00853-1)
Supplement: Supplementary file 1 — Additional file 1 [file 13102_2024_853_MOESM1_ESM.pdf]

Additional file 1

Injury registration

This questionnaire is about your past and current injuries. Read each question carefully. The form is divided into three parts.

1. **A serious injury** that occurred earlier than the year 2021
2. **An injury that led to time loss** from soccer training in 2021
3. **An injury problem that did not lead to time loss** from soccer training in 202

A serious injury, a time loss injury and an injury problem that did not lead to time loss are defined in the text below. If you have the slightest doubt about how to interpret these words or how to answer the questionnaire, ask your test leader.

Start by filling in the questions below.

Player code (to be filled in by the test leader)	
First and last name	
E-mail (will be used for the web-based questionnaire)	
Cell phone number (will be used for text reminders)	
Club	
Division	
What year and day were you born?<br>(E.g., 1998-04-14)	
Weight in Kg	
Height in Cm	
Number of years as a player in senior soccer	
Dominant leg (preferred kicking leg)<br>right/left/both	
Player position (specify one option: back, midfield, forward)	

1. Serious injuries earlier than the year 2021

The following questions are about **serious injuries that you suffered** before the year 2021 and which meant that you **were away from soccer for more than 3 months.**

Have you at any time **before the year 2021** suffered a serious injury that kept you away from soccer **for more than 3 months?**

YES ☐

NO ☐ If you answered no to this question, you can proceed to part 2.

When did you get hurt? Answer in years (e.g., 2019) ………………………………

If you answered yes, describe the type of injury and where you had the injury

(e.g., broken leg right foot) ……………………………………………………………

………………………………………………………………………………………..

……………………………………………………………………………..

Have you had more serious injuries? If so, describe the type of injury and where you had the injury:

……………………………………………………………………………………………….

…………………………………………………………………………………………

…………………………………………………………………………………………

2. Injury that led to time loss from soccer during the year 2021

The following question is about an injury or injuries which meant that you **had to refrain from training or a match during the year 2021**.

If you are **injured now** or have **an injury** that you sustained in **the last four weeks** which meant that **you had to refrain from** training or a match, **you start by registering that injury**.

If it is the same injury (a recurring injury) that caused **you time loss from match/training** in 2021, we want you to **report this** as a **separate injury** (injury 1, injury 2, etc.).

If you did not have **an injury during the year 2021** that led **to time loss from** training or a match **during the year 2021**, you can tick the following box ☐ and then proceed to part 3 of the questionnaire.

Injury 1

Has this injury occurred at any time within the last four weeks?

YES ☐

NO ☐

If yes, how many days or weeks ago? ________________

Are you injured today? ____________

Mark on the figure where you have had your injury.

Please tick the area in which you had/have an injury:

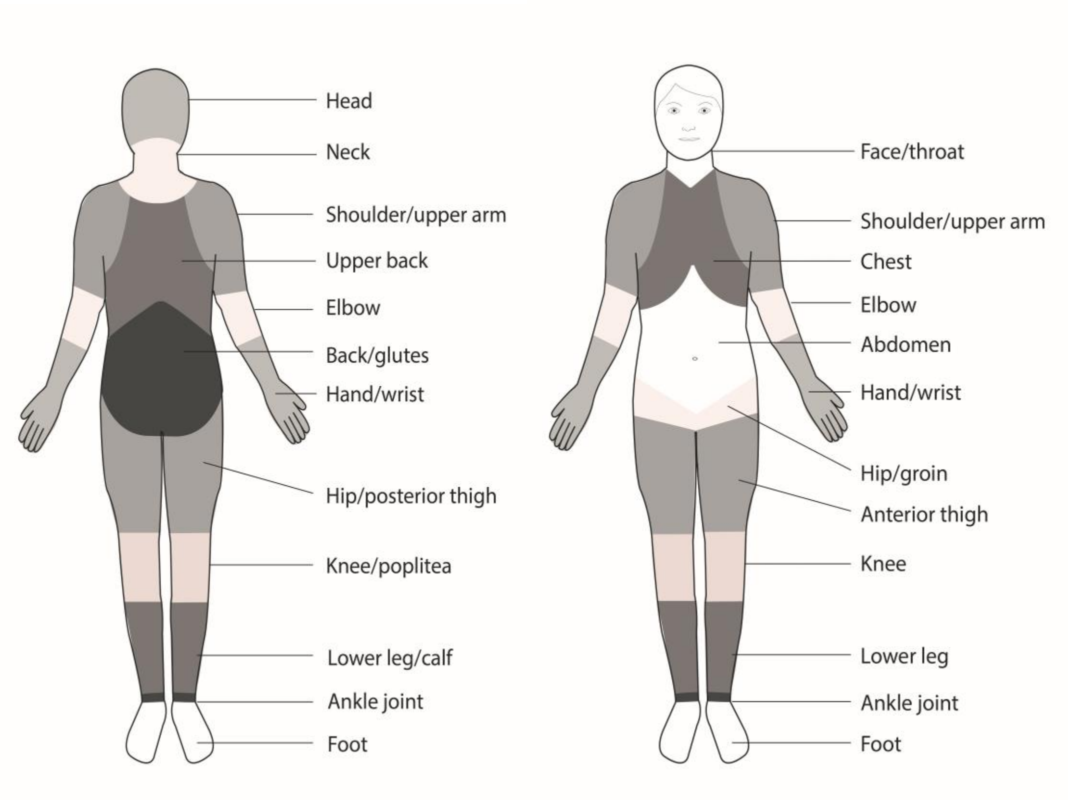

On which side of the body was the injury?

Right/left/both sides/not applicable.

Injury Severity-Time Loss

Estimate how many days you have had to miss training or matches due to this injury:

1–7 days
8–28 days
More than 28 days

If you **didn’t have any more injuries that led to time loss and absence from soccer**, you can now move on to part 3 of the questionnaire.

Injury 2

Please tick the area in which you had/have an injury:

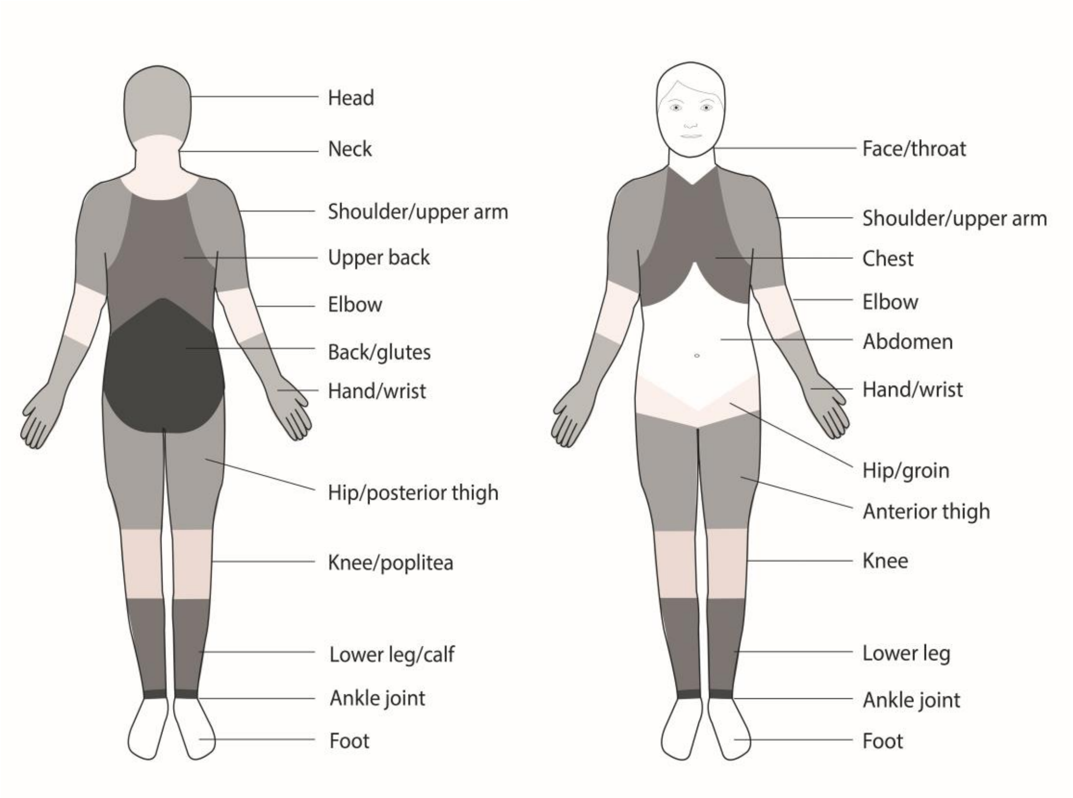

On which side of the body was the injury?

Right/left/both sides/not applicable.

Injury Severity-Time Loss

Estimate how many days you have had to miss training or matches due to this injury:

1–7 days
8–28 days
More than 28 days

If you **didn’t have any more injuries that led to time loss and absence from soccer**, you can now move on to part 3 of the questionnaire.

Injury 3

Please tick the area in which you had/have an injury:

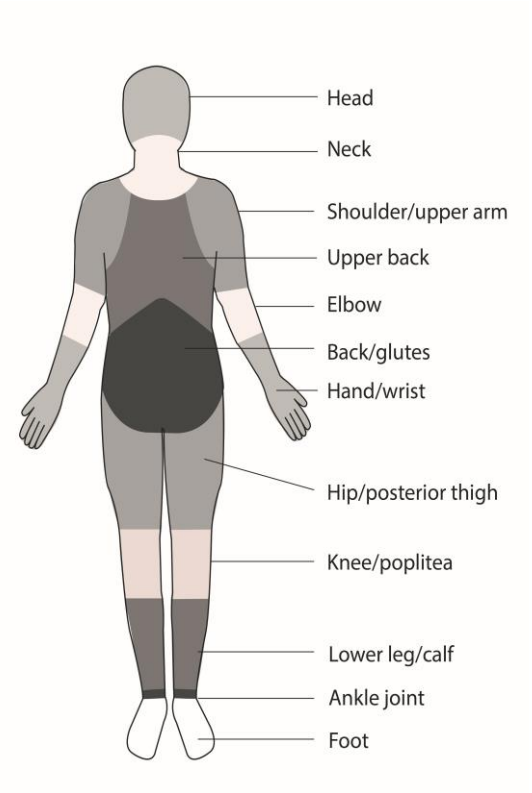

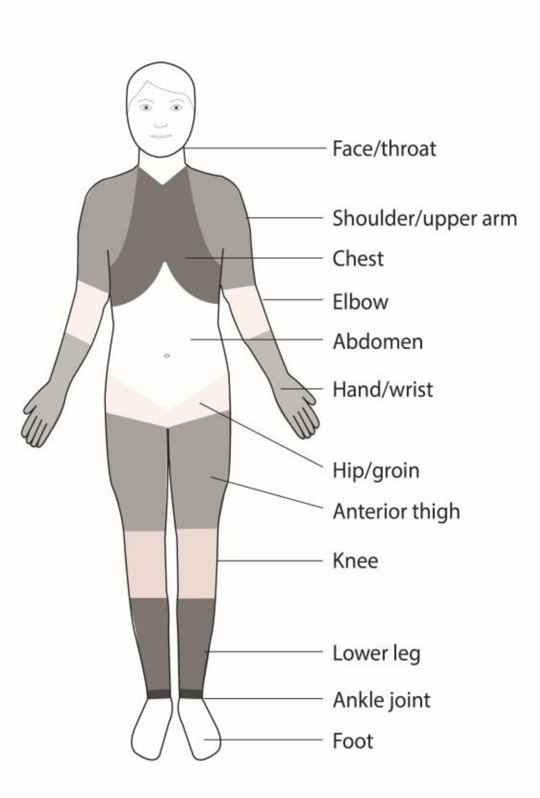

On which side of the body was the injury?

Right/left/both sides/not applicable.

Injury Severity-Time Loss

Estimate how many days you have had to miss training or matches due to this injury:

1–7 days
8–28 days
More than 28 days

If you **didn't have any more injuries that led to time loss and absence from soccer**, you can now move on to part 3 of the questionnaire.

3. Injury problems that did not lead to time loss from soccer in 2021:

The following questions are **about less serious injuries or injury problems** that you experienced in 2021 but **for which you did not have to give up training/match**.

We are interested in all types of physical complaints which meant that despite a certain degree **of pain/discomfort** you were **able to participate in all or some parts** of the match/training but **experienced difficulties in participating**, or **due to pain/discomfort performed worse.**

The term pain/discomfort refers to pain, ache, swelling, instability/giving way, locking etcetera.

If you **currently have an injury problem** or **have had** an injury problem in **the last four weeks,** you **start by registering that injury problem.**

If during the year 2021, you have **had several different** "injury problems that did not lead to time loss", you must report this as an additional "injury problem that did not lead to time loss" (i.e., injury problem 1, injury problem 2, etc.).

If you did not have any "injury problems", you do not need to answer any more questions.

Injury problem 1

Did this injury problem occur at any time within the last four weeks?

YES ☐

NO ☐

If yes, how many days or weeks ago? ________

How many days did this injury problem last? __________

Do you have an injury problem today? _____________

During 2021, did you have any injury problem
that lasted 2 weeks or more: yes/no

If yes, how many weeks: ________

Was your injury problem recurring in 2021?
(i.e., for periods that disappeared and then returned): yes/no

If yes, how often: ________________

* Please tick the area in which you had/have an injury:

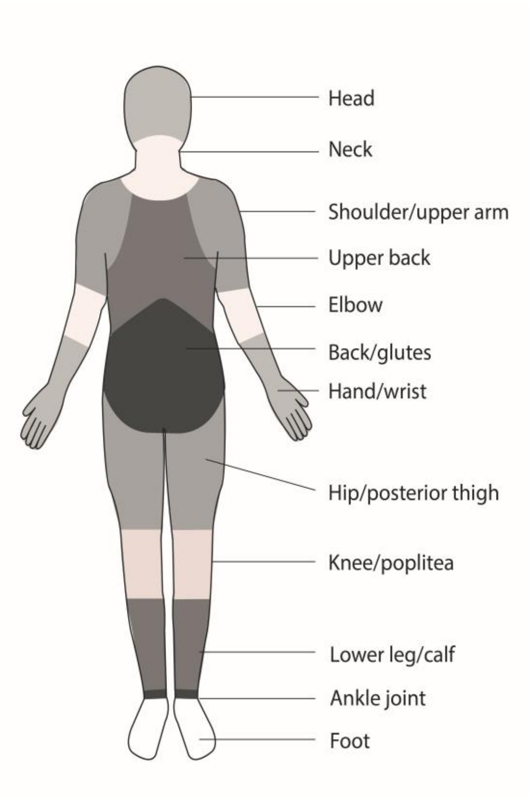

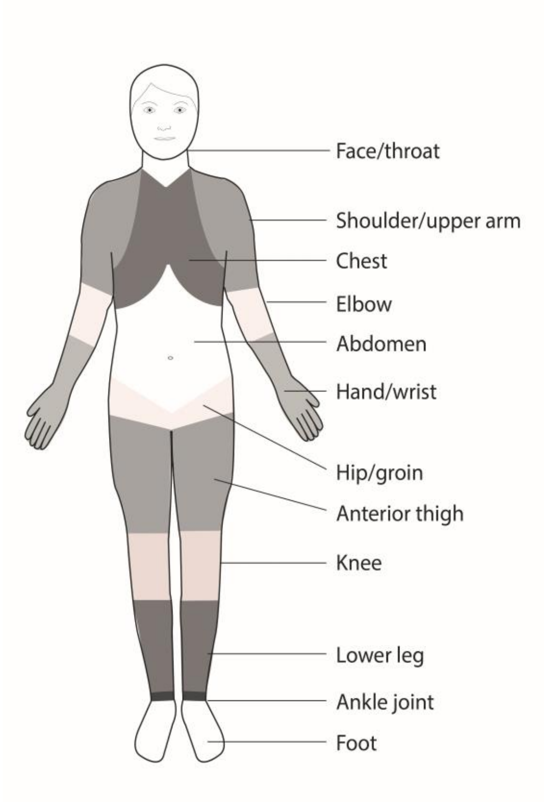

*On which side of the body was the injury?

Right/left/both sides/not applicable

If you **didn't have any more injury problems** in 2021, you can now submit the questionnaire.

Injury problem 2

Did you have any injury problem that lasted 2 weeks or more: yes/no

If yes, how many weeks: ________

If yes, was your injury problem recurring during the season?
(i.e., for periods that disappeared and then returned): yes/no

If yes, how often: _____________

* Please tick the area in which you had/have an injury:

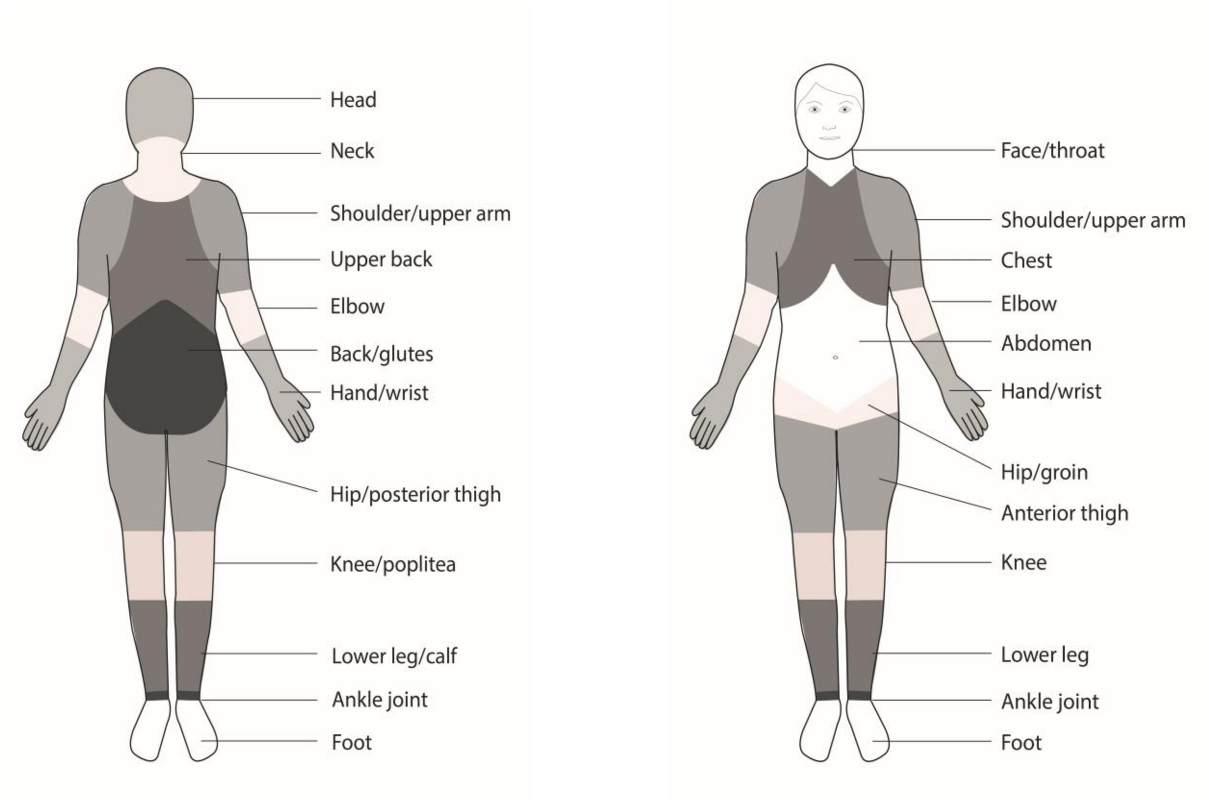

*On which side of the body was the injury?

Right/left/both sides/not applicable.

If you **didn't have any more injury problems** in 2021, you can now submit the questionnaire.

Injury problem 3

Did you have any injury problem that lasted 2 weeks or more: yes/no

If yes, how many weeks: ________

If yes, was your injury problem recurring during the season?
(i.e., for periods that disappeared and then returned): yes/no

If yes, how often: _____________

* Please tick the area in which you had/have an injury:

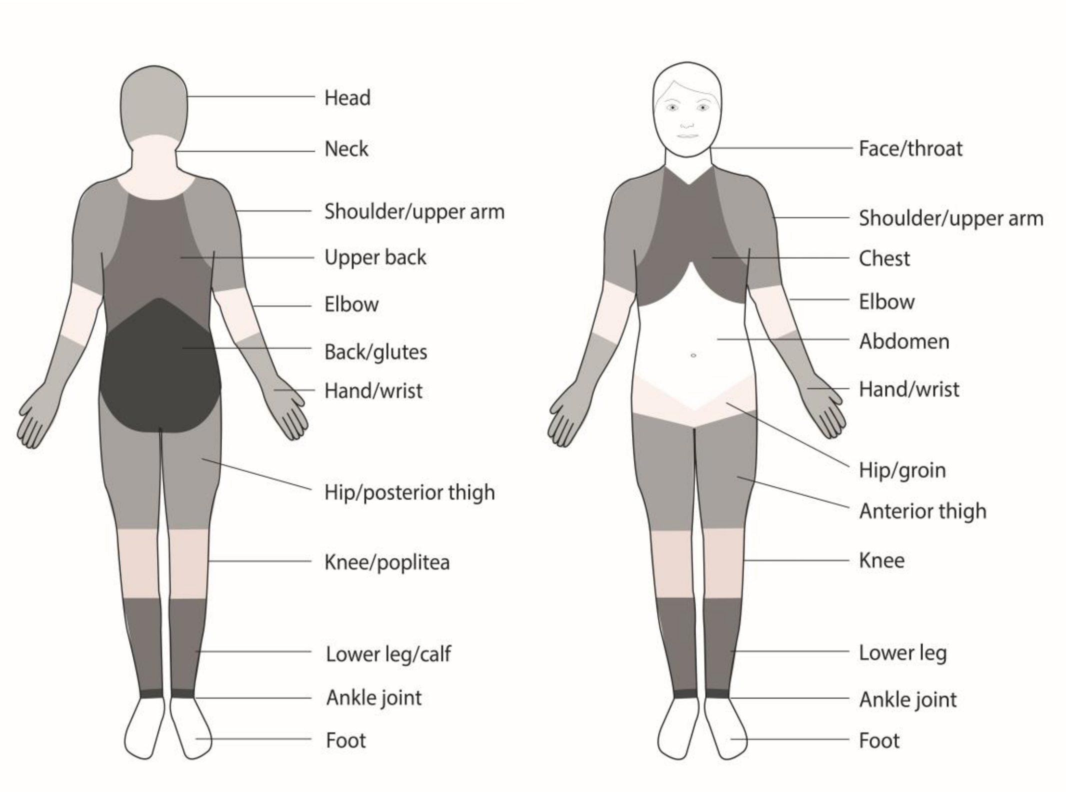

*On which side of the body was the injury?

Right/left/both sides/not applicable.

If you **had more injury problems** in 2021, you could continue to write on the back of this paper.
