# Supplementary material for: Factors associated with the Single Leg Squat test in female soccer players: a cross-sectional study
Source: BMC Sports Sci Med Rehabil. 2024 Apr 2;16:76. doi: 10.1186/s13102-024-00853-1 (PMC10985895; doi:10.1186/s13102-024-00853-1)
Supplement: Supplementary file 2 — Additional file 2 [file 13102_2024_853_MOESM2_ESM.pdf]

Karolinska Institutet

Risk factors for injuries in female soccer: sleep, fear avoidance, stress, and anxiety.

Aim and time consumption of the questionnaire.

The purpose of this survey is to investigate whether certain personal factors such as sleep, fear avoidance, stress and anxiety can predict which soccer players have an increased risk of injury.

The questionnaire is estimated to take 10-15 minutes to answer and consists of four different parts, distributed on 49 questions. Please note that you must answer all questions.

Thank you for contributing to research in sports medicine.

Karolinska Institutet

Risk factors for injuries in female soccer: sleep, fear avoidance, stress, and anxiety.

Part 1. AFAQ

We are interested in your feelings or thoughts <u>when in pain as a result of a sports injury.</u> Using the following scale, please indicate the degree to which you have these thoughts and feeling <u>when you are in pain due to a sports injury</u>.

1. I will never be able to play as I did before my injury.

1. Not at all	2. To a slight degree	3. To a moderate degree	4.To a great degree	5. Completely agree
◯	◯	◯	◯	◯

2. I am worried about my role with the team changing.

1. Not at all	2. To a slight degree	3. To a moderate degree	4.To a great degree	5. Completely agree
◯	◯	◯	◯	◯

3. I am worried about what other people will think of me if I don’t perform at the same level.

1. Not at all	2. To a slight degree	3. To a moderate degree	4.To a great degree	5. Completely agree
◯	◯	◯	◯	◯

4. I am not sure what my injury is.

1. Not at all	2. To a slight degree	3. To a moderate degree	4.To a great degree	5. Completely agree
◯	◯	◯	◯	◯

5. I believe that my current injury has jeopardized my future athletic abilities.

1. Not at all	2. To a slight degree	3. To a moderate degree	4.To a great degree	5. Completely agree
◯	◯	◯	◯	◯

6. I am not comfortable going back to play until I am 100%.

1. Not at all	2. To a slight degree	3. To a moderate degree	4.To a great degree	5. Completely agree
◯	◯	◯	◯	◯

7. People don't understand how serious my injury is.

1. Not at all	2. To a slight degree	3. To a moderate degree	4.To a great degree	5. Completely agree
◯	◯	◯	◯	◯

8. I don't know if I am ready to play.

1. Not at all	2. To a slight degree	3. To a moderate degree	4.To a great degree	5. Completely agree
◯	◯	◯	◯	◯

9. I worry if I go back to play too soon I will make my injury worse.

1. Not at all	2. To a slight degree	3. To a moderate degree	4.To a great degree	5. Completely agree
◯	◯	◯	◯	◯

10. When my pain is intense, I worry that my injury is a very serious one.

1. Not at all	2. To a slight degree	3. To a moderate degree	4.To a great degree	5. Completely agree
○	○	○	○	○

Karolinska Institutet

Risk factors for injuries in female soccer: sleep, fear avoidance, stress, and anxiety.

Part 2. PSS-14

The questions in this scale ask you about your feelings and thoughts during the last month.

In each case, you will be asked to indicate how often you felt or thought a certain way. Although some of the questions are similar, there are differences between them and you should treat each one as a separate question.

The best approach is to answer each question fairly quickly. That is, don't try to count up the number of times you felt a particular way, but rather indicate the alternative that seems like a reasonable estimate.

1. In the last month, how often have you been upset because of something that happened unexpectedly?

0. Never	1. Almost never	2. Sometimes	3. Fairly often	4. Very often
○	○	○	○	○

2. In the last month, how often have you felt that you were unable to control the important things in your life?

0. Never	1. Almost never	2. Sometimes	3. Fairly often	4. Very often
○	○	○	○	○

3. In the last month, how often have you felt nervous and "stressed"?

0. Never	1. Almost never	2. Sometimes	3. Fairly often	4. Very often
○	○	○	○	○

4. In the last month, how often have you dealt successfully with irritating life hassles?

0. Never	1. Almost never	2. Sometimes	3. Fairly often	4. Very often
○	○	○	○	○

5. In the last month, how often have you felt that you were effectively coping with important changes that were occurring in your life?

0. Never	1. Almost never	2. Sometimes	3. Fairly often	4. Very often
○	○	○	○	○

6. In the last month, how often have you felt confident about your ability to handle your personal problems?

0. Never	1. Almost never	2. Sometimes	3. Fairly often	4. Very often
○	○	○	○	○

7. In the last month, how often have you felt that things were going your way?

0. Never	1. Almost never	2. Sometimes	3. Fairly often	4. Very often
○	○	○	○	○

8. In the last month, how often have you found that you could not cope with all the things that you had to do?

0. Never	1. Almost never	2. Sometimes	3. Fairly often	4. Very often
○	○	○	○	○

9. In the last month, how often have you been able to control irritations in your life?

0. Never	1. Almost never	2. Sometimes	3. Fairly often	4. Very often
○	○	○	○	○

10. In the last month, how often have you felt that you were on top of things?

0. Never	1. Almost never	2. Sometimes	3. Fairly often	4. Very often
○	○	○	○	○

11. In the last month, how often have you been angered because of things that happened that were outside of your control?

0. Never	1. Almost never	2. Sometimes	3. Fairly often	4. Very often
○	○	○	○	○

12. In the last month, how often have you found yourself thinking about things that you have to accomplish?

0. Never	1. Almost never	2. Sometimes	3. Fairly often	4. Very often
○	○	○	○	○

13. In the last month, how often have you been able to control the way you spend your time?

0. Never	1. Almost never	2. Sometimes	3. Fairly often	4. Very often
○	○	○	○	○

14. In the last month, how often have you felt difficulties were piling up so high that you could not overcome them?

0. Never	1. Almost never	2. Sometimes	3. Fairly often	4. Very often
○	○	○	○	○

Karolinska Institutet

Risk factors for injuries in female soccer: sleep, fear avoidance, stress, and anxiety.

Part 3. PSQI

The following questions relate to your usual sleep habits during the past month only. Your answers should indicate the most accurate reply for the majority of days and nights in the past month.

1. During the past month, when have you usually gone to bed at night? (e.g.,22.30)

2. During the past month, how long (in minutes) has it usually taken you to fall asleep each night?

<15 min	16-30 min	31-60 min	>60 min
○	○	○	○

3. During the past month, when have you usually gotten up in the morning? (e.g., 07.00)

4. During the past month, how many hours of actual sleep did you get at night?

>7 h	6-7 h	5-6 h	<5 h
◯	◯	◯	◯

5. During the past month, how often have you had trouble sleeping because you cannot get to sleep within 30 minutes?

None	<1 time/week	1-2 times/week	≥ 3 times/week
◯	◯	◯	◯

6. During the past month, how often have you had trouble sleeping because you wake up in the middle of the night or early morning?

None	<1 time/week	1-2 times/week	≥ 3 times/week
◯	◯	◯	◯

7. During the past month, how often have you had trouble sleeping because you have to get up to use the bathroom?

None	<1 time/week	1-2 times/week	≥ 3 times/week
◯	◯	◯	◯

8. During the past month, how often have you had trouble sleeping because you cannot breathe comfortably?

None	<1 time/week	1-2 times/week	≥ 3 times/week
◯	◯	◯	◯

9. During the past month, how often have you had trouble sleeping because you cough or snore loudly?

None	<1 time/week	1-2 times/week	≥ 3 times/week
◯	◯	◯	◯

10. During the past month, how often have you had trouble sleeping because you feel too cold?

None	<1 time/week	1-2 times/week	≥ 3 times/week
○	○	○	○

11. During the past month, how often have you had trouble sleeping because you feel too hot?

None	<1 time/week	1-2 times/week	≥ 3 times/week
○	○	○	○

12. During the past month, how often have you had trouble sleeping because you had bad dreams?

None	<1 time/week	1-2 times/week	≥ 3 times/week
○	○	○	○

13. During the past month, how often have you had trouble sleeping because you have pain?

None	<1 time/week	1-2 times/week	≥ 3 times/week
○	○	○	○

14. During the past month, how often have you had trouble sleeping due to other reason(s)?

None	<1 time/week	1-2 times/week	≥ 3 times/week
○	○	○	○

Please describe.

15. During the past month, how often have you taken medicine to help you sleep?

None	<1 time/week	1-2 times/week	≥ 3 times/week
○	○	○	○

16. During the past month, how often have you had trouble staying awake while driving, eating meals, or engaging in social activity?

None	<1 time/week	1-2 times/week	≥ 3 times/week
○	○	○	○

17. During the past month, how much of a problem has it been for you to keep up enough enthusiasm to get things done?

None	<1 time/week	1-2 times/week	≥ 3 times/week
○	○	○	○

18. During the past month, how would you rate your sleep quality overall?

Very good	Fairly good	Fairly bad	Very bad
○	○	○	○

Karolinska Institutet

Risk factors for injuries in female soccer: sleep, fear avoidance, stress, and anxiety.

Part 4. GAD-7

Over the last 2 weeks, how often have you been bothered by the following problems?

1. Feeling nervous, anxious or on edge

0. Not at all ◯ 1. Several days ◯ 2. More than half the days ◯ 3. Nearly every day ◯

2. Not being able to stop or control worrying

0. Not at all ◯ 1. Several days ◯ 2. More than half the days ◯ 3. Nearly every day ◯

3. Worrying too much about different things

0. Not at all ◯ 1. Several days ◯ 2. More than half the days ◯ 3. Nearly every day ◯

4. Trouble relaxing

0. Not at all　1. Several days　2. More than half the days　3. Nearly every day

5. Being so restless that it is hard to sit still

0. Not at all　1. Several days　2. More than half the days　3. Nearly every day

6. Becoming easily annoyed or irritable

0. Not at all　1. Several days　2. More than half the days　3. Nearly every day

7. Feeling afraid as if something awful might happen

0. Not at all　1. Several days　2. More than half the days　3. Nearly every day
