# Supplementary material for: Factors associated with the Single Leg Squat test in female soccer players: a cross-sectional study
Source: BMC Sports Sci Med Rehabil. 2024 Apr 2;16:76. doi: 10.1186/s13102-024-00853-1 (PMC10985895; doi:10.1186/s13102-024-00853-1)
Supplement: Supplementary file 3 — Additional file 3 [file 13102_2024_853_MOESM3_ESM.pdf]

Additional file 3

```
*******************************************************************************************
*MULTI-SEGMENTAL MODELS FOR THE DOMINANT AND NON-DOMINANT LEG
*******************************************************************************************

*Categorisation of some variables:

//Variable GAD-7:
gen GAD7_cat2=.
replace GAD7_cat2=0 if GAD7<5
replace GAD7_cat2=1 if GAD7>=5 & GAD7<10
replace GAD7_cat2=2 if GAD7>=10 & GAD7<.
label define GAD7_cat2_label 0 "No GAD" 1 "Mild GAD" 2 "Moderate/Severe GAD"
label values GAD7_cat2 GAD7_cat2_label


//Variable div_cat
//already defined:gen div_cat=.
//already defined:replace div_cat=1 if div<2
//already defined:replace div_cat=2 if div>=2 & div<3
//already defined:replace div_cat=3 if div>=3 & div<.
//already defined:label define div_label 1 "Damallasvenskan" 2 "Elitettan" 3 "Div 1"
//already defined:label values div_cat div_label


//variable Age_cat
sum Age, detail //for the whole material: Q1=19, Q2=22 and Q3=25
gen Age_cat=.
replace Age_cat=1 if Age<20
replace Age_cat=2 if Age>=20 & Age<25
replace Age_cat=3 if Age>=25 & Age<.
label define Age_label 1 "<20" 2 "20-24" 3 ">24"
label values Age_cat Age_label


//Variable NDL_ADF_cat:
sum NDL_ADF_cat, detail // Q1=42.36025 , Q2=44.85346 , Q3=47.8549
gen NDL_ADF_cat=.
replace NDL_ADF_cat=1 if NDL_ADF<42.37
replace NDL_ADF_cat=2 if NDL_ADF>=42.37 & NDL_ADF<44.86
replace NDL_ADF_cat=3 if NDL_ADF>=44.86 & NDL_ADF<47.85
replace NDL_ADF_cat=4 if NDL_ADF>=47.85 & NDL_ADF<.

label define NDL_ADF_label 1 "<42.37" 2 "42.37-44.85" 3 "44.86-47.84" 4 ">=47.85"
label values NDL_ADF_cat NDL_ADF_label



//Variable DL_ADF_cat:
sum DL_ADF, detail //Q1=42.23914, Q2=44.96423  , Q3=47.33566

gen DL_ADF_cat=.
replace DL_ADF_cat=1 if DL_ADF<42.239
replace DL_ADF_cat=2 if DL_ADF>=42.239 & DL_ADF<44.964
```

```
replace DL_ADF_cat=3 if DL_ADF>=44.964 & DL_ADF<47.335
replace DL_ADF_cat=4 if DL_ADF>=47.335 & DL_ADF<.

label define DL_ADF_label 1 "<42.239" 2 "42.239-44.963" 3 ">=44.964-47.334" 4 ">=47.335"
label values DL_ADF_cat DL_ADF_label



//Variable PSQI_cat2:
sum PSQI, detail //According to the PSQI scale the cut off >5 can be used to distinguish
between good and poor sleepers
gen PSQI_cat2=.
replace PSQI_cat2=0 if PSQI <=5
replace PSQI_cat2=1 if PSQI >=6 & PSQI<.
label define PSQI_cat2_label 0 "<=5" 1 ">5"
label values PSQI_cat2 PSQI_cat2_label


//Variable Nm3Bwm
gen NDL_Nm3BWm_100=NDL_Nm3BWm*100
gen DL_Nm3BWm_100=DL_Nm3BWm*100


**********************************************************************************************
*MCNEMAR´S CHI2 TEST FOR PARIED DATA ON NOMINAL LEVEL TO INVESTIGATE IF THERE IS ANY
SIGNIFICANT DIFFERENCE IN THE OUTCOME WHEN PERFORMING A SLS TEST ON THE DL OR NDL
**********************************************************************************************
tab NDL_SLSfailpass DL_SLSfailpass
mcci 52 85 26 91 //p=0.00
mcc NDL_SLSfailpass DL_SLSfailpass //p=0.00


**********************************************************************************************
*WILCOXON SIGNED RANK TEST FOR PAIRED (DEPENDENT)NON-NORMALLY DISTRIBUTED DATA TO INVESTIGATE
THE DIFFERENCES IN STRENGTH AND ANKLE DORSIFLEXION FOR THE DOMINANT AND
*NON-DOMINANT LEG
**********************************************************************************************
signrank NDL_Nm3BWm_100=DL_Nm3BWm_100 //p=0.0260 (Wilcoxon signed-rank test Is a paired test
for non normaly distributed data)
signrank NDL_ADF=DL_ADF //p=0.1091 (Wilcoxon signed-rank test Is a paired test for non normaly
distributed data)
ttest NDL_ADF=DL_ADF //p=0.1333 (paired t test: this variable is normally distributed and
could use t test statistics)


**********************************************************************************************
*WILCOXON RANK SUM TEST FOR UNPAIRED (INDEPENDENT) NON-NORMALLY DISTRIBUTED DATA TO
INVESTIGATE THE DIFFERENCE IN STRENGTH AND ADF BETWEEN THOSE WHO FAILED AND PASSED THE SLS
*IN EACH SEPARATE LEG
**********************************************************************************************
ranksum NDL_Nm3BWm_100, by(NDL_SLSfailpass) //p=0.023 (Wilcoxon rank sum test is an unpaired
test for non-normally distributed data)
ttest NDL_Nm3BWm_100, by(NDL_SLSfailpass) //p=0.04
ttest NDL_Nm3BWm_100, by(NDL_SLSfailpass)unequal //p=0.04

ranksum DL_Nm3BWm_100, by(DL_SLSfailpass) //p=0.06
ttest DL_Nm3BWm_100, by(DL_SLSfailpass) //p=0.02
ttest DL_Nm3BWm_100, by(DL_SLSfailpass) unequal //p=0.04
```

```
ranksum NDL_ADF, by(NDL_SLSfailpass) //p=0.36
ranksum DL_ADF, by(DL_SLSfailpass) //p=0.04

ttest NDL_ADF, by(NDL_SLSfailpass) //p=0.40 (this variable is normally distributed and could use t test statistics)
ttest NDL_ADF, by(NDL_SLSfailpass) unequal //p=0.40(The assumption of equal variances can be optionally relaxed in the unpaired two-sample case.)
ttest DL_ADF, by(DL_SLSfailpass) //p=0.02
ttest DL_ADF, by(DL_SLSfailpass) unequal //p=0.02
```

****

***MODEL BUILDING WITH THE COMMAND STEPWISE, A BACKWARD LOGISTIC REGRESSION MODEL FOR MULTI-SEGMENTALL PASS/FAIL MODEL: NON-DOMINANT LEG**

****

*** STEP 1.**

```
//Looking at univariable logistic regression model for all possible covariate (see table 2) and evaluating the
//variables with descriptive statistics (i.e Outliers, normal distribution)

//The variable div
logistic NDL_SLSfailpass div
logistic NDL_SLSfailpass i.div
tab NDL_SLSfailpass div


//The variable Age
logistic NDL_SLSfailpass Age
sum Age if NDL_SLSfailpass==0, detail
sum Age if NDL_SLSfailpass==1, detail
graph box Age, over (NDL_SLSfailpass) //Three outliers
scatter Age Subject, mlabel(Subject)
list Subject NDL_SLSfailpass Age
logistic NDL_SLSfailpass Age if Subject!=76 & Subject !=143 & Subject !=12 // Conclusion: No significant change in OR, CI, Std error or P when the outliers are excluded.

histogram Age, normal //not normally distributed
symplot Age // skewed to the right
qnorm Age, grid //focus on the tails, fairly normally distributed
pnorm Age, grid //Focus on the middle of the distribution, fairly normally distributed
sktest Age // P<0.05, thus we can reject the hypothesis that Age is normally distributed
tabstat Age, stat(n mean sd p25 p50 p75)
display 22.27559-22 //Mean-Median = .27559 (if skewed to the right/positive skeweness the median is lower than the mean)
display 25-22 //Upper quartile-median= 3
display 22-19 //median-lower quartile= 3


 //Conlusion: The variable Age is not normally distributed
```

```
//The categorized variable Age,
tab NDL_SLSfailpass Age_cat
logistic NDL_SLSfailpass Age_cat



//The variable NDL_ADF
logistic NDL_SLSfailpass NDL_ADF
sum NDL_ADF if NDL_SLSfailpass==0, detail
sum NDL_ADF if NDL_SLSfailpass==1, detail
graph box NDL_ADF, over (NDL_SLSfailpass) //Three outliers
scatter NDL_ADF Subject, mlabel(Subject) //Two outliers: 109 and 251
list Subject NDL_SLSfailpass NDL_ADF
logistic NDL_SLSfailpass NDL_ADF if Subject!=251 & Subject !=109 // Conclusion: No significant
change in OR, CI, Std error or P when the outliers are excluded.

histogram NDL_ADF, normal //Looks relatively normally distributed
symplot NDL_ADF //looks ok
qnorm NDL_ADF, grid //looks ok
pnorm NDL_ADF, grid //looks ok
sktest NDL_ADF // p=0.93, thus we can not reject the hypothesis that Age is normally
distributed
tabstat NDL_ADF, stat(n mean sd p25 p50 p75)
di 45.01126-44.85346 //Mean-Median = .16 (if skewed to the right/positive skeweness the median
is lower than the mean)
di 47.8549- 44.85346 //Upper quartile-median= 3
di 44.85346 -42.36025 //median-lower quartile= 2.5



              //Conlusion: The variable NDL_ADF can bee seen as normally distributed


//The variable NDL_ADF_cat
logistic NDL_SLSfailpass NDL_ADF_cat
logistic NDL_SLSfailpass i.NDL_ADF_cat
tab NDL_SLSfailpass NDL_ADF_cat



//The variable NDL_Nm3BWm_100
logistic NDL_SLSfailpass NDL_Nm3BWm_100
sum NDL_Nm3BWm_100 if NDL_SLSfailpass==0, detail
sum NDL_Nm3BWm_100 if NDL_SLSfailpass==1, detail
graph box NDL_Nm3BWm_100, over (NDL_SLSfailpass) //Two outliers
scatter NDL_Nm3BWm_100 Subject, mlabel(Subject) //Two outliers: 261, 90, (12 is low but not an
outlier)
list Subject NDL_SLSfailpass NDL_Nm3BWm_100
logistic NDL_SLSfailpass NDL_Nm3BWm_100 if Subject!=261 & Subject !=90 & Subject !=41 //
Conclusion: No significant change in OR, CI, Std error or P when the outliers are excluded.

histogram NDL_Nm3BWm_100, normal //Looks relatively normally distributed
symplot NDL_Nm3BWm_100 //The variable is skewed to the right
qnorm NDL_Nm3BWm_100, grid //focus on the tails, looks skewed to the right
pnorm NDL_Nm3BWm_100, grid //Focus on the middle of the distribution, looks ok
sktest NDL_Nm3BWm_100 // p=0.0001, thus we can reject the hypothesis that NDL_Nm3BWm is
normally distributed
tabstat NDL_Nm3BWm_100, stat(n mean sd p25 p50 p75)
```

```
di 98.97194 -97.53 //Mean-Median = 1.44194 (if skewed to the right/positive skeweness the
median is lower than the mean)
di di 113.09- 97.53 //Upper quartile-median= 15.56
di 97.53 -82.91  //median-lower quartile= 14.62



//Conlusion: Visually the variable looks normally distributed but is rejected
//on the sktest, the variable will be seen as not normally distributed


//The dichotomized variables NDL_SI_1, NDL_TLI_4W, NDL_TLI_1, NDL_TLI_total_time, NDL_IP_4W
logistic NDL_SLSfailpass NDL_SI_1
tab NDL_SLSfailpass NDL_SI_1

logistic NDL_SLSfailpass NDL_TLI_4W
tab NDL_SLSfailpass NDL_TLI_4W //this variable has to low cell count and should be taken away
from the final model

logistic NDL_SLSfailpass NDL_TLI_1
tab NDL_SLSfailpass NDL_TLI_1

logistic NDL_SLSfailpass NDL_TLI_total_time
tab NDL_SLSfailpass NDL_TLI_total_time

logistic NDL_SLSfailpass NDL_IP_4W
tab NDL_SLSfailpass NDL_IP_4W



//The variable AFAQ
logistic NDL_SLSfailpass AFAQ
sum AFAQ if NDL_SLSfailpass==0, detail
sum AFAQ if NDL_SLSfailpass==1, detail
graph box AFAQ, over (NDL_SLSfailpass) //One outlier
scatter AFAQ Subject, mlabel(Subject) //Three subjects with the same values are seen: 80, 43
or 55
list Subject NDL_SLSfailpass AFAQ
logistic NDL_SLSfailpass AFAQ if Subject!=80 & Subject !=43 & Subject !=55 // Conclusion: No
significant change in OR, CI, Std error or P when the outliers are excluded.


histogram AFAQ, normal // Skewed to the right
symplot AFAQ // Skewed to the right
qnorm AFAQ, grid //focus on the tails, looks fairly normally distributed
pnorm AFAQ, grid //Focus on the middle of the distribution, looks fairly normally distributed
sktest AFAQ //P>0.05, thus we cannot reject the hypothesis that AFAQ is normally distributed
tabstat AFAQ, stat(n mean sd p25 p50 p75)
di  23.56746 -23 //Mean-Median = .56746
di 28-23 //Upper quartile-median = 5
di 23-19 //median-lower quartile= 4

//Conclusion: the variable AFAQ are fairly normally distributed but a little bit
//skewed to the right.
```

```
//The variable PSS14
logistic NDL_SLSfailpass PSS14
sum PSS14 if NDL_SLSfailpass==0, detail
sum PSS14 if NDL_SLSfailpass==1, detail
graph box PSS14, over (NDL_SLSfailpass) //Three outliers
scatter PSS14 Subject, mlabel(Subject) //Three outliers: 46, 52, 189
list Subject NDL_SLSfailpass PSS14
logistic NDL_SLSfailpass PSS14 if Subject!=46 & Subject !=52 & Subject !=189  //No significant
change in OR, CI, Std error or P when the outliers are excluded.

histogram PSS14, normal // Fairly normally distributed but a little bit Skewed to the left
symplot PSS14 // Skewed to the left
qnorm PSS14, grid //focus on the tails, does not look normally distributed
pnorm PSS14, grid //Focus on the middle of the distribution, does not look normally
distributed
sktest PSS14 //P>0.05, thus we cannot reject the hypothesis that PSS14 is normally distributed
tabstat PSS14, stat(n mean sd p25 p50 p75)
di 31.74206 -32 //Mean-Median = -.25794
di 34-32 //Upper quartile-median = 2
di 32-29.5 //median-lower quartile= 2.5

//Conclusion: Divergent results, the variable PSS14 will not be treated as normally
distributed.


//The variable PSQI
logistic NDL_SLSfailpass PSQI
sum PSQI if NDL_SLSfailpass==0, detail
sum PSQI if NDL_SLSfailpass==1, detail
graph box PSQI, over (NDL_SLSfailpass) // Six outliers
scatter PSQI Subject, mlabel(Subject) //five outliers: 92, 225, 173, 246, 31, 154
list Subject NDL_SLSfailpass PSQI
list Subject NDL_SLSfailpass PSQI if NDL_SLSfailpass==0
logistic NDL_SLSfailpass PSQI if Subject!=92 & Subject!=225 & Subject!=154 & Subject!=173 &
Subject!=246  & Subject!=31

// Conclusion: No significant change in OR, CI, Std error or P when the outliers are excluded.

histogram PSQI, normal // Skewed to the right
symplot PSQI // Skewed to the right
qnorm PSQI, grid //focus on the tails, not ok
pnorm PSQI, grid //Focus on the middle of the distribution, not ok
sktest PSQI //P<0.05, thus we can reject the hypothesis that PSQI is normally distributed
tabstat PSQI, stat(n mean sd p25 p50 p75)
di 5.198413-5 //Mean-Median = .198413
di 7-5 //Upper quartile-median = 2
di 5-3 //median-lower quartile= 2

//Conclusion: the variable PSQI is not normally distributed

logistic NDL_SLSfailpass PSQI_cat2
tab NDL_SLSfailpass PSQI_cat2
```

```
//The variable GAD7
logistic NDL_SLSfailpass GAD7_cat
logistic NDL_SLSfailpass i.GAD7_cat
tab NDL_SLSfailpass GAD7_cat

graph box GAD7, over (NDL_SLSfailpass) //Seven outliers
scatter GAD7 Subject, mlabel(Subject) //Looking at the five highest outliers: 45, 31, 7, 230,
75
list Subject NDL_SLSfailpass GAD7
list Subject NDL_SLSfailpass GAD7 if NDL_SLSfailpass==1
list Subject NDL_SLSfailpass GAD7 if NDL_SLSfailpass==0
logistic NDL_SLSfailpass GAD7 if Subject !=45 & Subject !=31 & Subject!=7 & Subject!=230 &
Subject!=75

// Conclusion: There is some changes in OR, CI, Std error and P when the outliers are
excluded, but nothing major

histogram GAD7, normal // Skewed to the right and not normally distributed
symplot GAD7 // Skewed to the right
qnorm GAD7, grid //focus on the tails, not ok
pnorm GAD7, grid //Focus on the middle of the distribution, not ok
sktest GAD7 //P<0.05, thus we can reject the hypothesis that GAD/ is normally distributed
tabstat GAD7, stat(n mean sd p25 p50 p75)
di 6.115079-5 //Mean-Median = 1.115079
di 8-5 //Upper quartile-median = 3
di 5-3 //median-lower quartile= 2


//The variable GAD7 is not normally distributed


//The variable GAD7_cat
logistic NDL_SLSfailpass GAD7_cat2
logistic NDL_SLSfailpass i.GAD7_cat2
tab NDL_SLSfailpass GAD7_cat2
```

***STEP 2: CHECKING FOR COLLINEARITY AND LINEAR ASSUMPTIONS**

```
*Check for collinearity between the continous independent variables; This will be checked in
the stepwise command

*Check for correlation between the dichotomous independent variables;
corr NDL_SI_1 NDL_TLI_4W NDL_TLI_1 NDL_TLI_total_time NDL_IP_4W
spearman NDL_SI_1 NDL_TLI_4W NDL_TLI_1 NDL_TLI_total_time NDL_IP_4W //There is a correlation
between some variables

corr NDL_TLI_4W NDL_TLI_1 //corr=0.30
spearman NDL_TLI_4W NDL_TLI_1 //spearman=0.30
kap NDL_TLI_4W NDL_TLI_1 //kap=0.17

corr NDL_TLI_4W NDL_TLI_total_time //corr=0.23
spearman NDL_TLI_4W NDL_TLI_total_time //spearman=0.25
kap NDL_TLI_4W NDL_TLI_total_time //kap=0.09

corr NDL_TLI_1 NDL_TLI_total_time //corr=0.77
spearman NDL_TLI_1 NDL_TLI_total_time //spearman=0.82
```

```
kap NDL_TLI_1 NDL_TLI_total_time //kap=0.44

corr NDL_SI_1 NDL_TLI_4W NDL_TLI_1 NDL_IP_4W PSQI_cat2 //no correlation seen
spearman NDL_SI_1 NDL_TLI_4W NDL_TLI_1 NDL_IP_4W PSQI_cat2 //no correlation seen
kap NDL_SI_1 PSQI_cat2 //no correlation seen
kap NDL_TLI_4W PSQI_cat2 //no correlation seen
kap NDL_TLI_1 PSQI_cat2 //no correlation seen
kap NDL_IP_4W PSQI_cat2 //no correlation seen

//Spearman's correlation coefficients were interpreted as follows:
//0.0-0.3:negligible correlation,
//0.3-0.5:low correlation,
//0.5-0.7:moderate correlation,
//0.7-0.9:high correlation,
//0.9-1.0:very high correlation.

//conclusion: The two variabled NDL_TLI_1 and NDL_TLI_total_time are correlated and should not
be //used in the same model. The variable NDL_TLI_1 is choosen to stay in the model as it´s p
value //is under 0.20 for the dominant leg.


*Test the assumptions for liniarity for each continuous variable in the model; thus that the
logit increases/decreases linearly
//as a function of the continuous covariates.


//FIRSTLY, linearity is checked with plots:
logistic NDL_SLSfailpass i.div Age NDL_ADF NDL_Nm3BWm_100 NDL_SI_1 NDL_TLI_4W NDL_TLI_1
NDL_IP_4W AFAQ PSS14 PSQI_cat2 i.GAD7_cat2
predict logits_NDL, xb

scatter logits_NDL Age || lowess logits_NDL Age //Ok with linearity up to 30 yr. after that we
have to outliers which affects the linearity. Will be dichotomized

                                                                                  //due to we
dichotomized this variable in the knee model
scatter logits_NDL NDL_ADF || lowess logits_NDL NDL_ADF //looks ok for linearity
scatter logits_NDL NDL_Nm3BWm_100 || lowess logits_NDL NDL_Nm3BWm_100 //There is some non-
linearity in this plot due to two outliers, see below. However we will see the variable as
linear.


scatter logits_NDL NDL_Nm3BWm_100 if Subject!=261 & Subject !=12 || lowess logits_NDL
NDL_Nm3BWm_100 if Subject!=261 & Subject !=12 // H
scatter logits_NDL AFAQ || lowess logits_NDL AFAQ //looks ok for linearity
scatter logits_NDL PSS14 || lowess logits_NDL PSS14 //Looks ok
scatter logits_NDL PSQI || lowess logits_NDL PSQI //this variable will be dichotomiez due to
we found a good reference for a cut-off


//In order to see if transformation could change Age and NDL NDL_Nm3BWm_100:

//Age: Transformation
gen Age_sqrt= sqrt(Age)
boxtid logit NDL_SLSfailpass Age_sqrt //p=0.30
gen Age_sq=Age^2
boxtid logit NDL_SLSfailpass Age_sq //p=0.27
gen Age_log=log(Age)
```

```
boxtid logit NDL_SLSfailpass Age_log //p=0.31

//Age: Visual check of transformation
logistic NDL_SLSfailpass i.div Age NDL_ADF NDL_Nm3BWm NDL_SI_1 NDL_TLI_4W NDL_IP_4W AFAQ PSS14
PSQI i.GAD7_cat2 Age_sqrt Age_sq Age_log
predict logits_NDL_Age, xb

scatter logits_NDL_Age Age_sqrt || lowess logits_NDL_Age Age_sqrt //does not look ok
scatter logits_NDL_Age Age_sq || lowess logits_NDL_Age Age_sq //does not look ok
scatter logits_NDL_Age Age_log || lowess logits_NDL_Age Age_log //does not look ok



//NDL_Nm3BWm_100: Transformation
gen NDL_Nm3BWm_100_sqrt= sqrt(NDL_Nm3BWm_100)
boxtid logit NDL_SLSfailpass NDL_Nm3BWm_100_sqrt //p=0.72
gen NDL_Nm3BWm_100_sq=NDL_Nm3BWm_100^2
boxtid logit NDL_SLSfailpass NDL_Nm3BWm_100_sq //p=0.59
gen NDL_Nm3BWm_100_log=log(NDL_Nm3BWm_100)
boxtid logit NDL_SLSfailpass NDL_Nm3BWm_100_log //p=0.48


//NDL_Nm3BWm_100: Visual check of transformation
logistic NDL_SLSfailpass i.div Age NDL_ADF NDL_Nm3BWm_100 NDL_SI_1 NDL_TLI_4W NDL_IP_4W AFAQ
PSS14 PSQI i.GAD7_cat2 NDL_Nm3BWm_sqrt NDL_Nm3BWm_sq NDL_Nm3BWm_log
predict logits_NDL_Nm3BWm_100, xb

scatter logits_NDL_Nm3BWm NDL_Nm3BWm_sqrt || lowess logits_NDL_Nm3BWm NDL_Nm3BWm_sqrt //does
not look ok
scatter logits_NDL_Nm3BWm NDL_Nm3BWm_sq || lowess logits_NDL_Nm3BWm NDL_Nm3BWm_sq //does not
look ok
scatter logits_NDL_Nm3BWm NDL_Nm3BWm_log || lowess logits_NDL_Nm3BWm NDL_Nm3BWm_log //does not
look ok


//SECONDLY, the boxtid test is also used to check if the variables fullfills the assumption of
linearity. If P>0.05 the assumption is fulfilled:
boxtid logit NDL_SLSfailpass Age NDL_Nm3BWm_100 AFAQ PSS14 PSQI GAD7 NDL_ADF // *convergence
not achieved r(430)?
boxtid logit NDL_SLSfailpass Age NDL_Nm3BWm_100 AFAQ PSS14 PSQI GAD7 // Age, p=0.93;
NDL_Nm3BWm, p=0.18; AFAQ, p=0.55; PSS14, p=0.25; PSQI, p=0.34; GAD7, p=0.89

//the variable NDL_ADF can not be calculated:


boxtid logit NDL_SLSfailpass Age //p=0.79
boxtid logit NDL_SLSfailpass NDL_Nm3BWm_100 //p=0.99
boxtid logit NDL_SLSfailpass AFAQ //p=0.41
boxtid logit NDL_SLSfailpass PSS14 //p=0.02
boxtid logit NDL_SLSfailpass PSQI //p=0.06
boxtid logit NDL_SLSfailpass GAD7 //P=0.22
boxtid logit NDL_SLSfailpass NDL_ADF //p=0.997

//In the final model the continous variables age, PSQI and GAD will be categorized. Therfore
those variables are taken away from the boxtid test.
```

boxtid logit NDL_SLSfailpass NDL_Nm3BWm_100 NDL_ADF AFAQ PSS14 //STATA gives the following message: Hessian is not negative semidefinite. Separating AFAQ and PSS14 and runs //the calculation again.

boxtid logit NDL_SLSfailpass NDL_Nm3BWm_100 NDL_ADF AFAQ // All variables are non significant
boxtid logit NDL_SLSfailpass NDL_Nm3BWm_100 NDL_ADF PSS14 // NDL_Nm3BWm_100 and NDL_ADF are non significant but PSS14 are significant p=0.028 indicating a non linearity //relationship with NDL_SLSfailpass.

//ADF: Transformation

gen NDL_ADF_sqrt=sqrt (NDL_ADF) // Square root transformation in order to get linearity
boxtid logit NDL_SLSfailpass NDL_ADF_sqrt //*insufficient observations r(2001); ??

gen NDL_ADF_sq=NDL_ADF^2
boxtid logit NDL_SLSfailpass NDL_ADF_sq //p=0.002

gen NDL_ADF_log=log(NDL_ADF)
boxtid logit NDL_SLSfailpass NDL_ADF_log //Insufficient observation???

//conclusion: //Age will be categorized due to the visual inspection, and due to that we dichotomized it for the knee model. //ADF will stay in the model as it is, this due to the visual inspection where it looked ok for linearity//NDL_Nm3BWm_100 will stay in the model as it is due to the combination of an ok visual inspection and the box-tidwell test.
//PSS14 will stay in the model as the visual inspection was good even though the box-tidwell test was n.s

***STEP 3: STEPWISE LOGISTISTIC REGRESSION**
//stepwise logistic without excluding outliers. No outliers affected the univariate models in a significant way.
xi: stepwise, pr(.2) pe(.05): logistic NDL_SLSfailpass (i.div) (i.Age_cat) NDL_ADF NDL_Nm3BWm_100 NDL_SI_1 NDL_TLI_1 NDL_IP_4W AFAQ PSS14 (i.PSQI_cat2) (i.GAD7_cat2)

*STEP 4: CHECKING FOR INTERACTION AMONG THE VARIABLES THAT ARE LEFT IN THE FINAL MODEL FROM ABOVE (in an exploratory purpose)
xi: stepwise, pr(.2) pe(.05): logistic NDL_SLSfailpass (i.div) (i.Age_cat) NDL_ADF NDL_Nm3BWm_100 NDL_SI_1 NDL_TLI_1 NDL_IP_4W AFAQ PSS14 (i.PSQI_cat2) (i.GAD7_cat2)

//The variables that are left in the model are: div, NDL_SI_1, NDL_IP_4W and NDL_Nm3BWm_100

//div vs NDL_SI_1
gen div_NDL_SI_1=div*NDL_SI_1
logistic NDL_SLSfailpass div NDL_SI_1 div_NDL_SI_1 //p=0.75

//div vs NDL_IP_4W
gen div_NDL_IP_4W=div*NDL_IP_4W
logistic NDL_SLSfailpass div NDL_IP_4W div_NDL_IP_4W //p=0.27

//div vs NDL_Nm3BWm_100
gen div_NDL_Nm3BWm_100=div*NDL_Nm3BWm_100
logistic NDL_SLSfailpass div NDL_Nm3BWm_100 div_NDL_Nm3BWm_100 //p=0.90

//NDL_SI_1 vs NDL_IP_4W

```
gen NDL_SI_1_NDL_IP_4W=NDL_SI_1*NDL_IP_4W
logistic NDL_SLSfailpass NDL_SI_1 NDL_IP_4W NDL_SI_1_NDL_IP_4W //p=0.99

//NDL_SI_1 vs NDL_Nm3BWm_100
gen NDL_SI_1_NDL_Nm3BWm_100=NDL_SI_1*NDL_Nm3BWm_100
logistic NDL_SLSfailpass NDL_SI_1 NDL_Nm3BWm_100 NDL_SI_1_NDL_Nm3BWm_100 //p=0.044******(OBS!
Hip strenght modifies the effect on SI. Hip strength is an effect modifier)

//NDL_IP_4W vs NDL_Nm3BWm_100
gen NDL_IP_4W_NDL_Nm3BWm_100=NDL_IP_4W*NDL_Nm3BWm_100
logistic NDL_SLSfailpass NDL_IP_4W NDL_Nm3BWm_100 NDL_IP_4W_NDL_Nm3BWm_100 // p=0.68


//Looking at the differences with and without the interaction term:
xi: stepwise, pr(.2) pe(.05): logistic NDL_SLSfailpass (i.div) (i.Age_cat) NDL_ADF
NDL_Nm3BWm_100 NDL_SI_1 NDL_TLI_1 NDL_IP_4W AFAQ PSS14 (i.PSQI_cat2) (i.GAD7_cat2)
xi: stepwise, pr(.2) pe(.05): logistic NDL_SLSfailpass (i.div) (i.Age_cat) NDL_ADF
NDL_Nm3BWm_100 NDL_SI_1 NDL_TLI_1 NDL_IP_4W AFAQ PSS14 (i.PSQI_cat2) (i.GAD7_cat2)
NDL_SI_1_NDL_Nm3BWm_100

lincom NDL_SI_1 + NDL_SI_1_NDL_Nm3BWm_100 //OR=.019

lincom NDL_SI_1 + 50*NDL_SI_1_NDL_Nm3BWm_100 //OR=0.08 significant (for those who have had a
SI (SI=1))
lincom NDL_SI_1 + 75*NDL_SI_1_NDL_Nm3BWm_100 //OR=0.17 significant
lincom NDL_SI_1 + 100*NDL_SI_1_NDL_Nm3BWm_100 //OR=0.37 significant
lincom NDL_SI_1 + 125*NDL_SI_1_NDL_Nm3BWm_100 //OR=0.77 n.s
lincom NDL_SI_1 + 150*NDL_SI_1_NDL_Nm3BWm_100 //OR=1.62 n.s
lincom NDL_SI_1 + 200*NDL_SI_1_NDL_Nm3BWm_100 //OR=7.22 n.s

//Changing the coding for SI to 0=Yes SI and 1=No SI. This to see how the OR looks like with
integer:
gen NDL_SI_opp_NDL_Nm3BWm_100=NDL_SI_opp*NDL_Nm3BWm_100
logistic NDL_SLSfailpass NDL_SI_opp NDL_Nm3BWm_100 NDL_SI_opp_NDL_Nm3BWm_100 //the variable SI
has an OR=45, Sd 66 and CI 2 to 805. this point estimate is very uncertain.

logistic NDL_SLSfailpass (i.div) (i.Age_cat) NDL_ADF NDL_Nm3BWm_100 NDL_SI_opp NDL_TLI_1
NDL_IP_4W AFAQ PSS14 (i.PSQI_cat2) (i.GAD7_cat2) NDL_SI_opp_NDL_Nm3BWm_100
logistic NDL_SLSfailpass (i.div) NDL_ADF NDL_Nm3BWm_100 NDL_SI_opp  NDL_IP_4W
NDL_SI_opp_NDL_Nm3BWm_100 //OR for NDL_SI_opp=60 with an CI of 3-1200


//Looking at the interaction term descriptively:

gen SI0_CLAM=.
replace SI0_CLAM= NDL_Nm3BWm_100 if NDL_SI_opp==0
gen SI1_CLAM=.
replace SI1_CLAM= NDL_Nm3BWm_100 if NDL_SI_opp==1 //generating two variable which depicts hip
strength for those with and without any previous SI.


//SI0_CLAM=52 observations and SI1_CLAM=194 observations.

sum SI0_CLAM if NDL_SLSfailpass==1, detail //Observations=14, Mean=108, Sd=29, Range 62-151
sum SI0_CLAM if NDL_SLSfailpass==0, detail //Observations=38, Mean=101, Sd=27, Range 40-160
graph box SI0_CLAM, over (NDL_SLSfailpass) //The spread is much greater in the group who
passed the SLS test.
ranksum SI0_CLAM, by (NDL_SLSfailpass) //p=0.41
```

sum SI1_CLAM if NDL_SLSfailpass==1, detail //Observations=100, Mean=94, Sd=21, Range 57-203
sum SI1_CLAM if NDL_SLSfailpass==0, detail //Observations=94, Mean=102, Sd=21, Range 56-162
graph box SI1_CLAM, over (NDL_SLSfailpass) // The spread is much greater in the group who passed the SLS test, but there is one great outlier in SLS=1
ranksum SI1_CLAM, by(NDL_SLSfailpass) //p=0.0045

//Conclusion: the OR of 60 and a CI of 3-1200 is unrealistic and an uncertain point estimate.
//In SI1 there is a difference in spread which could be a reason for the great CI. The interaction term will not be applicated.

***STEP 5: CHECKING FOR MODEL ASSUMPTIONS AND FIT**
xi: stepwise, pr(.2) pe(.05): logistic NDL_SLSfailpass (i.div) (i.Age_cat) NDL_ADF NDL_Nm3BWm_100 NDL_SI_1 NDL_TLI_1 NDL_IP_4W AFAQ PSS14 (i.PSQI_cat2) (i.GAD7_cat2)

*FIRSTLY, Check for collinearity; This will be checked in the stepwise command but could also be checked like this:
regress PSS14 AFAQ NDL_ADF NDL_Nm3BWm_100
vif // If vif =>5 there is coliniarity. In this test vif=1.00
collin PSS14 AFAQ NDL_ADF NDL_Nm3BWm_100 //If the variables are non correlated both vif and tolerance
//should be close to 1. In this test both tolerance and vif is close to 1.0

*SECONDLY, checking for outlying, high leverage and influential points
//Starts by looking at residual and leverage plots.
//A rule of thumb if the sample size is high, a point is influential if the Pearson and Deviance residual is >2 and that the leverage hat value is >2-3 times
//higher than the average of the leverage (ref: https://stats.oarc.ucla.edu/stata/webbooks/logistic/chapter3/lesson-3-logistic-regression-diagnostics-2/)
xi: stepwise, pr(.2) pe(.05): logistic NDL_SLSfailpass (i.div) (i.Age_cat) NDL_ADF NDL_Nm3BWm_100 NDL_SI_1 NDL_TLI_1 NDL_IP_4W AFAQ PSS14 (i.PSQI_cat2) (i.GAD7_cat2)

logistic NDL_SLSfailpass i.div i.Age_cat NDL_ADF NDL_Nm3BWm_100 NDL_SI_1 NDL_TLI_1 NDL_IP_4W AFAQ PSS14 i.PSQI_cat2 i.GAD7_cat2
predict phat_NDL, p
predict rstandard_NDL, rstandard
scatter rstandard_NDL phat_NDL, mlabel(Subject) //Produces a plot for the standardized Pearson residuals: Subject 35 and 138 are high and 203 is low.

scatter rstandard_NDL Subject, mlabel(Subject) //Produces an indexplot for the standardized Person residuals: same as above
predict dv_NDL, dev
scatter dv_NDL phat_NDL, mlab(Subject) //Produces a deviance residual plot, another type of plot that should give the same results as the Pearson residuals:
//Same as above except for 203

predict hat_NDL, hat
scatter hat_NDL phat_NDL, mlab(Subject) //Produces a leverage plot: the mean leverage is approximately 0.06 so subjects arund 0.15 should be checked up.
//subject 261 has the highest leverage.
scatter hat_NDL Subject, mlab(Subject) //Produces a leverage indexplot: Subjects 261 has high leverage.

```
//Looking at the values for all the different variables for subject
clist if Subject==261

//Conclusion: no specifik errors in registration found


//Comparing the models with and without Subject 261
xi: stepwise, pr(.2) pe(.05): logistic NDL_SLSfailpass (i.div) (i.Age_cat) NDL_ADF
NDL_Nm3BWm_100 NDL_SI_1 NDL_TLI_1 NDL_IP_4W AFAQ PSS14 (i.PSQI_cat2) (i.GAD7_cat2)
xi: stepwise, pr(.2) pe(.05): logistic NDL_SLSfailpass (i.div) (i.Age_cat) NDL_ADF
NDL_Nm3BWm_100 NDL_SI_1 NDL_TLI_1 NDL_IP_4W AFAQ PSS14 (i.PSQI_cat2) (i.GAD7_cat2) if
Subject!=261

//Conclusion: None of the subjects with large resiuduals had high leverage. //the variable
div_3 changed to n.s otherwise some n.s variables were included in the model.


*THIRDLY, Model fit
//the variable
xi: stepwise, pr(.2) pe(.05): logistic NDL_SLSfailpass (i.div) (i.Age_cat) NDL_ADF
NDL_Nm3BWm_100 NDL_SI_1 NDL_TLI_1 NDL_IP_4W AFAQ PSS14 (i.PSQI_cat2) (i.GAD7_cat2)
estat gof, group(10) //the gof test is non-significant p=0.78. (As the model contains a
continous variable the estat gof, gropu (10 can be used))
//A significant finding indicates lack of fit and a non-significant result rules out a gross
lack of fit. The test is very sensitive to
//fairly small fit discrepancies in a larger sample. Thus, a significant results in such cases
may not signal a serious fit problem in such cases
linktest //The model has a reasonable fit if _hat is significant, the results in this model is
p=0.000. The model is adequate if _hatsq P>0.05, this model has p=0.15.
//A significant result for _hatq means that an alternativ binary regression model should be
considered (quadratic function, not using the logit),
//it may also indicate that important predictors have been omitted or are represented
incorrectly in the model


//*Check of linearity in final model:
logistic NDL_SLSfailpass i.div i.Age_cat NDL_ADF NDL_Nm3BWm_100 NDL_SI_1 NDL_TLI_1 NDL_IP_4W
AFAQ PSS14 i.PSQI_cat2 i.GAD7_cat2
predict logits_NDL_final, xb
scatter logits_NDL NDL_Nm3BWm_100 || lowess logits_NDL NDL_Nm3BWm_100 //As above, this
variable has some linearity problems in the beginnin and end of the line,
// this is due to two correct outliers (one hig and one low).
boxtid logit NDL_SLSfailpass NDL_Nm3BWm_100 //p=0.99

//this is the final model
```

```
***********************************************************************************************
*MODEL BUILDING WITH THE COMMAND STEPWISE, A BACKWARD LOGISTIC REGRESSION MODEL FOR MULTI-
SEGMENTALL PASS/FAIL MODEL: DOMINANT LEG
***********************************************************************************************

* STEP 1.
//Looking at univariable logistic regression model for all possible covariate (see table 2)
and evaluating the //variables with descriptive statistics (i.e Outliers, normal distribution)

*CRUDE VALUES- SLS PASS/FAIL FOR ALL DL VARIABLES

//The variable div
logistic DL_SLSfailpass div
logistic DL_SLSfailpass i.div
tab DL_SLSfailpass div

//The variable Age
logistic DL_SLSfailpass  Age
sum Age if DL_SLSfailpass==0, detail
sum Age if DL_SLSfailpass==1, detail
graph box Age, over (DL_SLSfailpass) //Two outliers
scatter Age Subject, mlabel(Subject)
list Subject DL_SLSfailpass Age
logistic DL_SLSfailpass Age if Subject!=76 & Subject !=143 // Conclusion: No significant
change in OR, CI, Std error or P when the outliers are excluded.

histogram Age, normal //not normally distributed
symplot Age // skewed to the right
qnorm Age, grid //focus on the tails, fairly normally distributed
pnorm Age, grid //Focus on the middle of the distribution, fairly normally distributed
sktest Age // P<0.05, thus we can reject the hypothesis that Age is normally distributed
tabstat Age, stat(n mean sd p25 p50 p75)
display 22.27559 -22 //Mean-Median = .27559 (if skewed to the right/positive skeweness the
median is lower than the mean)
display 25-22 //Upper quartile-median= 3
display 22-19 //median-lower quartile= 3

//Conlusion: The variable Age is not normally distributed
//the variable Age_cat
tab Age_cat


//The variable DL_ADF
logistic DL_SLSfailpass  DL_ADF
sum DL_ADF if DL_SLSfailpass==0, detail
sum DL_ADF if DL_SLSfailpass==1, detail

graph box DL_ADF, over (DL_SLSfailpass) //Four outliers
scatter DL_ADF Subject, mlabel(Subject)
list Subject DL_SLSfailpass DL_ADF // subject: 64, 109, 6 and 258
logistic DL_SLSfailpass DL_ADF if Subject!=64 & Subject!=109 & Subject!=6 & Subject!=258 //
Conclusion: No greater significant change in OR, CI, Std error or P


                                                       // when the outliers are excluded.
```

```
histogram DL_ADF, normal //Fairly distributed, skewed to the left
symplot DL_ADF // skewed to the left
qnorm DL_ADF, grid //focus on the tails, skewed to the left
pnorm DL_ADF, grid //Focus on the middle of the distribution, fairly normally distributed
sktest DL_ADF // P>0.05, thus we can no reject the hypothesis that DL_ADF is normally
distributed
tabstat DL_ADF, stat(n mean sd p25 p50 p75)
di 44.76395-44.96423 //Mean-Median = -.20028 skewed to the left (if skewed to the
right/positive skeweness the median is lower than the mean)
di 47.33566-44.96423 //Upper quartile-median= 2.37143
di 44.96423- 42.23914 //median-lower quartile= 2.72509

//Conlusion: The variable DL_ADF is normally distributed with some skewness to the left



//The variable DL_ADF_cat
logistic DL_SLSfailpass DL_ADF_cat
logistic DL_SLSfailpass i.DL_ADF_cat
tab DL_SLSfailpass DL_ADF_cat


//the variable DL_Nm3BWm_100
logistic DL_SLSfailpass  DL_Nm3BWm_100
sum DL_Nm3BWm_100 if DL_SLSfailpass==0, detail
sum DL_Nm3BWm_100 if DL_SLSfailpass==1, detail

graph box DL_Nm3BWm_100, over (DL_SLSfailpass) //Four outliers
scatter DL_Nm3BWm_100 Subject, mlabel(Subject)
list Subject DL_SLSfailpass DL_Nm3BWm_100 // subject: 90, 226, 245 and 261
logistic DL_SLSfailpass DL_Nm3BWm_100 if Subject!=90 & Subject!=226 & Subject!=245 &
Subject!=261

//Conclusion: There is a significant change in OR when the outliers are excluded thus the
//OR will go from significant to n.s


histogram DL_Nm3BWm_100, normal //Fairly distributed, but skewed to the right
symplot DL_Nm3BWm_100 // skewed to the right
qnorm DL_Nm3BWm_100, grid //focus on the tails, skewed to the right
pnorm DL_Nm3BWm_100, grid //Focus on the middle of the distribution, fairly normally
distributed
sktest DL_Nm3BWm_100 // P<0.05, thus we can reject the hypothesis that DL_ADF is normally
distributed
tabstat DL_Nm3BWm_100, stat(n mean sd p25 p50 p75)
di 96.79302 - 95.75 //Mean-Median = 1.04 (if skewed to the right/positive skeweness the median
is lower than the mean)
di 110.905-95.75  //Upper quartile-median = 15.16
di 95.75-79.295  //median-lower quartile= 16.46


//The dichotomized variables DL_SI_1, DL_TLI_4W, DL_TLI_1, DL_IP_4W
logistic DL_SLSfailpass  DL_SI_1
tab DL_SLSfailpass  DL_SI_1

logistic DL_SLSfailpass  DL_TLI_4W
tab DL_SLSfailpass  DL_TLI_4W //this variable has low cell count for NDL and is therfore taken
away for the DL also (in the final model)
```

```
logistic DL_SLSfailpass  DL_TLI_1
tab DL_SLSfailpass  DL_TLI_1

logistic DL_SLSfailpass DL_TLI_total_time
tab DL_SLSfailpass DL_TLI_total_time

logistic DL_SLSfailpass  DL_IP_4W
tab DL_SLSfailpass  DL_IP_4W


//The variable AFAQ
logistic DL_SLSfailpass  AFAQ
sum AFAQ if DL_SLSfailpass==0, detail
sum AFAQ if DL_SLSfailpass==1, detail

graph box AFAQ, over (DL_SLSfailpass) //One outlier
scatter AFAQ Subject, mlabel(Subject)
list Subject DL_SLSfailpass AFAQ // subject: 80
logistic DL_SLSfailpass AFAQ if Subject!=80

//Conclusion: No significant change in OR, CI, Std error or P when the outliers are excluded.

histogram AFAQ, normal // Skewed to the right
symplot AFAQ // Skewed to the right
qnorm AFAQ, grid //focus on the tails, looks fairly normally distributed
pnorm AFAQ, grid //Focus on the middle of the distribution, looks fairly normally distributed
sktest AFAQ //P>0.05, thus we cannot reject the hypothesis that AFAQ is normally distributed
tabstat AFAQ, stat(n mean sd p25 p50 p75)
di  23.56746 -23 //Mean-Median = .56746
di 28-23 //Upper quartile-median = 5
di 23-19 //median-lower quartile= 4

//Conclusion: the variable AFAQ are fairly normally distributed but a little bit //skewed to
the right.


//The variable PSS14
logistic DL_SLSfailpass PSS14
sum PSS14 if DL_SLSfailpass==0, detail
sum PSS14 if DL_SLSfailpass==1, detail

graph box PSS14, over (DL_SLSfailpass) //Three outliers
scatter PSS14 Subject, mlabel(Subject)
list Subject DL_SLSfailpass PSS14 // subject: 46, 189 and 52
logistic DL_SLSfailpass PSS14 if Subject!=46 & Subject!=189 & Subject!=52


Conclusion:  No significant change in OR, CI, Std error or P when the outliers are excluded.


histogram PSS14, normal // Fairly normally distributed but a little bit Skewed to the left
symplot PSS14 // Skewed to the left
qnorm PSS14, grid //focus on the tails, does not look normally distributed
pnorm PSS14, grid //Focus on the middle of the distribution, does not look normally
distributed
sktest PSS14 //P>0.05, thus we cannot reject the hypothesis that PSS14 is normally distributed
tabstat PSS14, stat(n mean sd p25 p50 p75)
```

```
di 31.74206 -32 //Mean-Median = -.25794
di 34-32 //Upper quartile-median = 2
di 32-29.5 //median-lower quartile= 2.5

//Conclusion: Divergent results, the variable PSS14 will not be treated as normally
distributed.



//The variable PSQI
logistic DL_SLSfailpass  PSQI
sum PSQI if DL_SLSfailpass==0, detail
sum PSQI if DL_SLSfailpass==1, detail

graph box PSQI, over (DL_SLSfailpass) //Three outliers
scatter PSQI Subject, mlabel(Subject)
list Subject DL_SLSfailpass PSQI
list Subject DL_SLSfailpass PSQI if DL_SLSfailpass==0
list Subject DL_SLSfailpass PSQI if DL_SLSfailpass==1 // subject: 203, 92 and 225
logistic DL_SLSfailpass PSQI if Subject!=203 & Subject!=92 & Subject!=225

//Conclusion: No significant change in OR, CI, Std error or P when the outliers are excluded.

histogram PSQI, normal // Skewed to the right
symplot PSQI // Skewed to the right
qnorm PSQI, grid //focus on the tails, not ok
pnorm PSQI, grid //Focus on the middle of the distribution, not ok
sktest PSQI //P<0.05, thus we can reject the hypothesis that PSQI is normally distributed
tabstat PSQI, stat(n mean sd p25 p50 p75)
di 5.198413-5 //Mean-Median = .198413
di 7-5 //Upper quartile-median = 2
di 5-3 //median-lower quartile= 2


//Conclusion: the variable PSQI are not normally distributed

logistic DL_SLSfailpass PSQI_cat2
tab DL_SLSfailpass PSQI_cat2


//The variable GAD7_cat

logistic DL_SLSfailpass GAD7_cat
logistic DL_SLSfailpass i.GAD7_cat
tab DL_SLSfailpass  GAD7_cat   //OBS! THIS VARIABLE HAS A LOW CELL COUNT IN TABULATION AND
WILL BE TAKEN AWAY FROM FURTHER CALULATION!!!!!!!!!!!


histogram GAD7, normal // Skewed to the right and not normally distributed
symplot GAD7 // Skewed to the right
qnorm GAD7, grid //focus on the tails, not ok
pnorm GAD7, grid //Focus on the middle of the distribution, not ok
sktest GAD7 //P<0.05, thus we can reject the hypothesis that GAD/ is normally distributed
tabstat GAD7, stat(n mean sd p25 p50 p75)
di 6.115079-5 //Mean-Median = 1.115079
di 8-5 //Upper quartile-median = 3
di 5-3 //median-lower quartile= 2
//The variable GAD7 is not normally distributed
```

```
//The variable GAD7_cat2
logistic DL_SLSfailpass GAD7_cat2
logistic DL_SLSfailpass i.GAD7_cat2
tab DL_SLSfailpass GAD7_cat2
```

***STEP 2: CHECKING FOR COLLINEARITY AND LINEAR ASSUMPTIONS**

```
*Check for collinearity between the continous independent variables; This will be checked in the stepwise command

*Check for correlation between the dichotomous independent variables;

corr DL_SI_1 DL_TLI_4W DL_TLI_1 DL_TLI_total_time DL_IP_4W
spearman DL_SI_1 DL_TLI_4W DL_TLI_1 DL_TLI_total_time DL_IP_4W

corr DL_TLI_1 DL_TLI_total_time
spearman DL_TLI_1 DL_TLI_total_time
kap DL_TLI_1 DL_TLI_total_time  //conclusion: as for the non-dominant leg the variables DL_TLI_1 and DL_TLI_total_time is correlated.

//The variable NDL_TLI_1 is choosen to stay in the model as it´s p value
//is under 0.20 for the dominant leg. DL_TLI_4W was excluded in the model for the NDL due to low cell count, the variable will therfore be excluded in this model to.
//however, the cell count is also low for the DL.


corr DL_SI_1 DL_TLI_4W DL_TLI_1 DL_IP_4W PSQI_cat2 //no correlation seen
spearman DL_SI_1 DL_TLI_4W DL_TLI_1 DL_IP_4W PSQI_cat2 //no correlation seen
kap DL_SI_1 PSQI_cat2 //no correlation seen
kap DL_TLI_4W PSQI_cat2 //no correlation seen
kap DL_TLI_1 PSQI_cat2 //no correlation seen
kap DL_IP_4W PSQI_cat2 //no correlation seen


*Test the assumptions for liniarity for each continuous variable in the model; thus that the logit increases/decreases linearly
//as a function of the continuous covariates.

//FIRSTLY, linearity is checked with plots:
logistic DL_SLSfailpass i.div Age DL_ADF DL_Nm3BWm_100 DL_SI_1 DL_TLI_1 DL_IP_4W AFAQ PSS14 PSQI_cat2 i.GAD7_cat2
predict logits_DL, xb

scatter logits_DL Age || lowess logits_DL Age //does not look linear
scatter logits_DL DL_ADF || lowess logits_DL DL_ADF //looks ok
scatter logits_DL DL_Nm3BWm_100 || lowess logits_DL DL_Nm3BWm_100 //looks ok
scatter logits_DL AFAQ || lowess logits_DL AFAQ //looks ok
scatter logits_DL PSS14 || lowess logits_DL PSS14 //looks ok
scatter logits_DL PSQI || lowess logits_DL PSQI //looks ok


//SECONDLY, the boxtid test is used to check if the variables fullfills the assumption of linearity. If P>0.05 the assumption is fulfilled:
boxtid logit DL_SLSfailpass Age DL_Nm3BWm_100 AFAQ PSS14 PSQI GAD7 // Age, p=0.256; NDL_Nm3BWm_100, p=0.194; AFAQ, p=0.084; PSS14, p=0.335; PSQI, p=0.000; GAD7, p=0.775
```

//PSQI does not fulfill the assumption of linearity. This variable will be categorized in accordance to // a reference. //Conclusion: the variable Age will be categorized in accordance with the pass/fail model för NDL //and the knee model

***STEP 3: STEPWISE LOGISTISTIC REGRESSION**

```
//stepwise logistic without outliers. Outliers for the variable PSS14 and L_Nm3BWm_100 did affect the univariate models
xi: stepwise, pr(.2) pe(.05): logistic DL_SLSfailpass (i.div) (i.Age_cat) DL_ADF DL_Nm3BWm_100 DL_SI_1 DL_TLI_1 DL_IP_4W AFAQ PSS14 (i.PSQI_cat2) (i.GAD7_cat2)

//Excluding the subjects from PSS14
xi: stepwise, pr(.2) pe(.05): logistic DL_SLSfailpass (i.div) (i.Age_cat) DL_ADF DL_Nm3BWm_100 DL_SI_1 DL_TLI_1 DL_IP_4W AFAQ PSS14 (i.PSQI_cat2) (i.GAD7_cat2) if Subject!=42 & Subject!=189 & Subject!=63
//Conclusion: there is a small change in the p value for PSS14 which goes from p=0.019 to p=0.032

//Excluding the subjects from DL_Nm3BWm_100
xi: stepwise, pr(.2) pe(.05): logistic DL_SLSfailpass (i.div) (i.Age_cat) DL_ADF DL_Nm3BWm_100 DL_SI_1 DL_TLI_1 DL_IP_4W AFAQ PSS14 (i.PSQI_cat2) (i.GAD7_cat2) if Subject!=90 & Subject!=226 & Subject!=245 & Subject!=262
//Conclusion: the variable DL_Nm3BWm_100 will change from significant to n.s p=0.087 and the variable division will be included n.s
```

***STEP 4: CHECKING FOR INTERACTION AMONG THE VARIABLES THAT ARE LEFT IN THE FINAL MODEL FROM** ABOVE (in an exploratory purpose)

```
xi: stepwise, pr(.2) pe(.05): logistic DL_SLSfailpass (i.div) (i.Age_cat) DL_ADF DL_Nm3BWm_100 DL_SI_1 DL_TLI_1 DL_IP_4W AFAQ PSS14 (i.PSQI_cat2) (i.GAD7_cat2)
//The variables that are left in the model are: DL_TLI_1, GAD7, DL_ADF, DL_Nm3BWm_100, PSS14


//DL_TLI_1 vs GAD7
gen DL_TLI_1_GAD7_cat2=DL_TLI_1*GAD7_cat2
logistic DL_SLSfailpass DL_TLI_1 GAD7_cat2 DL_TLI_1_GAD7_cat2 //p=0.95
//DL_TLI_1 vs DL_ADF
gen DL_TLI_1_DL_ADF=DL_TLI_1*DL_ADF
logistic DL_SLSfailpass DL_TLI_1 DL_ADF DL_TLI_1_DL_ADF //p=0.67
//DL_TLI_1 vs DL_Nm3BWm_100
gen DL_TLI_1_DL_Nm3BWm_100=DL_TLI_1*DL_Nm3BWm_100
logistic DL_SLSfailpass DL_TLI_1 DL_Nm3BWm_100 DL_TLI_1_DL_Nm3BWm_100 //p=0.74
//DL_TLI_1 vs PSS14
gen DL_TLI_1_PSS14=DL_TLI_1*PSS14
logistic DL_SLSfailpass DL_TLI_1 PSS14 DL_TLI_1_PSS14 //p=0.042********** OR=400 and CI 1.7 to 89 000??????


//GAD7_cat2 vs DL_ADF
gen GAD7_cat2_DL_ADF=GAD7_cat2*DL_ADF
logistic DL_SLSfailpass GAD7_cat2 DL_ADF GAD7_cat2_DL_ADF //p=0.37
//GAD7_cat2 vs DL_Nm3BWm_100
gen GAD7_cat2_DL_Nm3BWm_100=GAD7_cat2*DL_Nm3BWm_100
logistic DL_SLSfailpass GAD7_cat2 DL_Nm3BWm_100 GAD7_cat2_DL_Nm3BWm_100 //p=0.45
```

```
//GAD7_cat2 vs PSS14
gen GAD7_cat2_PSS14=GAD7_cat2*PSS14
logistic DL_SLSfailpass GAD7_cat2 PSS14 GAD7_cat2_PSS14 //p=0.79


//DL_ADF vs DL_Nm3BWm_100
gen DL_ADF_DL_Nm3BWm_100=DL_ADF*DL_Nm3BWm_100
logistic DL_SLSfailpass DL_ADF DL_Nm3BWm_100 DL_ADF_DL_Nm3BWm_100 //P=0.51
//DL_ADF vs PSS14
gen DL_ADF_PSS14=DL_ADF*PSS14
logistic DL_SLSfailpass DL_ADF PSS14 DL_ADF_PSS14 //P=0.77


//DL_Nm3BWm_100 vs PSS14
gen DL_Nm3BWm_100_PSS14=DL_Nm3BWm_100*PSS14
logistic DL_SLSfailpass DL_Nm3BWm_100 PSS14 DL_Nm3BWm_100_PSS14 //p=0.43

//conclusion:One interaction found for DL_TLI_1 vs PSS14


logistic DL_SLSfailpass (i.div) (i.Age_cat) DL_ADF DL_Nm3BWm_100 DL_SI_1 DL_TLI_1 DL_IP_4W
AFAQ PSS14 (i.PSQI_cat2) (i.GAD7_cat2)
logistic DL_SLSfailpass (i.div) (i.Age_cat) DL_ADF DL_Nm3BWm_100 DL_SI_1 DL_TLI_1 DL_IP_4W
AFAQ PSS14 (i.PSQI_cat2) (i.GAD7_cat2) DL_TLI_1_PSS14
//the variable DL_TLI_1 has an OR of 294 and a CI of 0.9-91000

lincom DL_TLI_1 + DL_TLI_1_PSS14
lincom DL_TLI_1 + 5*DL_TLI_1_PSS14
lincom DL_TLI_1 + 10*DL_TLI_1_PSS14
lincom DL_TLI_1 + 15*DL_TLI_1_PSS14
lincom DL_TLI_1 + 20*DL_TLI_1_PSS14
lincom DL_TLI_1 + 25*DL_TLI_1_PSS14
lincom DL_TLI_1 + 30*DL_TLI_1_PSS14
lincom DL_TLI_1 + 35*DL_TLI_1_PSS14
lincom DL_TLI_1 + 40*DL_TLI_1_PSS14


logistic DL_SLSfailpass DL_ADF DL_Nm3BWm_100 DL_TLI_1 PSS14 (i.GAD7_cat2) DL_TLI_1_PSS14
// The OR for PSS14 with an interaction term is unrealistic and indicates an uncertain point
estimate

//Looking at the interaction term descriptively:
tab PSS14 DL_TLI_1
sum PSS14 if DL_SLSfailpass==0, detail
sum PSS14 if DL_SLSfailpass==1, detail

gen TLI0_PSS14=.
replace TLI0_PSS14= PSS14 if DL_TLI_1==0 //Generating
gen TLI1_PSS14=.
replace TLI1_PSS14= PSS14 if DL_TLI_1==1 //Generating

sum TLI0_PSS14 if DL_SLSfailpass==1, detail // Obs=86, Mean=32, Sd=3.4, Variance=11, Range=22-
40
sum TLI0_PSS14 if DL_SLSfailpass==0, detail //Obs=47, Mean=32, Sd=4.2, Variance=18, Range=24-
42
graph box TLI0_PSS14, over (DL_SLSfailpass) //The spread is greater for the SLS=0, there is
also have lesser subjects in that group
ranksum TLI0_PSS14, by (DL_SLSfailpass) //p=0.85
```

```
ttest TLI0_PSS14, by (DL_SLSfailpass) unequal //p=0.98

sum TLI1_PSS14 if DL_SLSfailpass==1, detail //Obs=86, Mean=31, Sd=3.7, variance=13, range=20-39
sum TLI1_PSS14 if DL_SLSfailpass==0, detail //obs=29, mean=33, sd=2.7, variance=7, range 27-39
graph box TLI1_PSS14, over (DL_SLSfailpass) //The spread is greater for the SLS=1, there is also more subjects in this group
ranksum TLI1_PSS14, by(DL_SLSfailpass) //p=0.01

ttest TLI1_PSS14, by(DL_SLSfailpass) unequal //       p=0.003



//Conclusion: The interaction term gives PSS14 an unrealistic and uncertain point estimate. perhaps due to //a power problem. There is also a greater difference in the number of subjects between the groups in TLI=0. //The spread differ in both groups which could give the uncertain point estimates.
```

***STEP 5: CHECKING FOR MODEL ASSUMPTIONS AND FIT**

```
xi: stepwise, pr(.2) pe(.05): logistic DL_SLSfailpass (i.div) (i.Age_cat) DL_ADF DL_Nm3BWm_100 DL_SI_1 DL_TLI_1 DL_IP_4W AFAQ PSS14 (i.PSQI_cat2) (i.GAD7_cat2)

*FIRSTLY, Check for collinearity; This will be checked in the stepwise command but could also be checked like this:
regress DL_ADF DL_Nm3BWm_100 AFAQ PSS14
vif //If vif =>5 there is coliniarity. Mean VIF is=1.03
collin DL_ADF DL_Nm3BWm_100 AFAQ PSS14 ////If the variables are non correlated both vif and tolerance //should be close to 1. They are that.



*SECONDLY, checking for outlying, high leverage and influential points
//Starts by looking at residual and leverage plots.
//A rule of thumb if the sample size is high, a point is influential if the Pearson and Deviance residual is >2 and that the leverage hat value is >2-3 times
//higher than the average of the leverage (ref: https://stats.oarc.ucla.edu/stata/webbooks/logistic/chapter3/lesson-3-logistic-regression-diagnostics-2/)
xi: stepwise, pr(.2) pe(.05): logistic DL_SLSfailpass (i.div) (i.Age_cat) DL_ADF DL_Nm3BWm_100 DL_SI_1 DL_TLI_1 DL_IP_4W AFAQ PSS14 (i.PSQI_cat2) (i.GAD7_cat2)

logistic DL_SLSfailpass i.div i.Age_cat DL_ADF DL_Nm3BWm_100 DL_SI_1 DL_TLI_4W DL_TLI_1 DL_IP_4W AFAQ PSS14 PSQI_cat2 i.GAD7_cat2
predict phat_DL, p
predict rstandard_DL, rstandard
scatter rstandard_DL phat_DL, mlabel(Subject) //Produces a plot for the standardized Pearson residuals:239, 268, 148, 112, 210, 187 seems to have high residuals.

scatter rstandard_DL Subject, mlabel(Subject) //Produces an indexplot for the standardized Person residuals:239, 268, 148, 112, 210, 187 have high residuals
predict dv_DL, dev
scatter dv_DL phat_DL, mlab(Subject) //Produces a deviance residual plot, another type of plot that should give the same results as the Pearson residuals: as above

predict hat_DL, hat
```

```
scatter hat_DL phat_DL, mlab(Subject) //Produces a leverage plot: The mean leverage seems to be 0.06, values around 0.15 are then of interest.

                                           // Subjects that have a high leverage are 155, 258, 12, 74, 108, 90, 144

                                           //None of the subjects with high residuals have large leverage
scatter hat_DL Subject, mlab(Subject) //Produces a leverage indexplot: same as above




//Looking at the values for all the different variables for subject
clist if Subject==155 | Subject==258 | Subject==12 | Subject==144 | Subject==74 | Subject==108


//Conclusion: No flaws found in registration, neither are any influential points found (high residuals + large leverage) //Comparing the models with and without Subject 258, 261, 246, 12, 144, 240, 74

xi: stepwise, pr(.2) pe(.05): logistic DL_SLSfailpass (i.div) (i.Age_cat) DL_ADF DL_Nm3BWm_100 DL_SI_1 DL_TLI_1 DL_IP_4W AFAQ PSS14 (i.PSQI_cat2) (i.GAD7_cat2)

xi: stepwise, pr(.2) pe(.05): logistic DL_SLSfailpass (i.div) (i.Age_cat) DL_ADF DL_Nm3BWm_100 DL_SI_1  DL_TLI_1 DL_IP_4W AFAQ PSS14 (i.PSQI_cat2) (i.GAD7_cat2) if Subject!=155
//Div and TLI will be included in a n.s way, IGAD7_cat2_2  will be included significantly
xi: stepwise, pr(.2) pe(.05): logistic DL_SLSfailpass (i.div) (i.Age_cat) DL_ADF DL_Nm3BWm_100 DL_SI_1  DL_TLI_1 DL_IP_4W AFAQ PSS14 (i.PSQI_cat2) (i.GAD7_cat2) if Subject!=258
//No changes
xi: stepwise, pr(.2) pe(.05): logistic DL_SLSfailpass (i.div) (i.Age_cat) DL_ADF DL_Nm3BWm_100 DL_SI_1  DL_TLI_1 DL_IP_4W AFAQ PSS14 (i.PSQI_cat2) (i.GAD7_cat2) if Subject!=12
//DL_TLI_1 will be included in a n.s way
xi: stepwise, pr(.2) pe(.05): logistic DL_SLSfailpass (i.div) (i.Age_cat) DL_ADF DL_Nm3BWm_100 DL_SI_1  DL_TLI_1 DL_IP_4W AFAQ PSS14 (i.PSQI_cat2) (i.GAD7_cat2) if Subject!=144
//DL_TLI_1 will be included in a n.s way, GAD7 will be included significantly for both categories,
xi: stepwise, pr(.2) pe(.05): logistic DL_SLSfailpass (i.div) (i.Age_cat) DL_ADF DL_Nm3BWm_100 DL_SI_1  DL_TLI_1 DL_IP_4W AFAQ PSS14 (i.PSQI_cat2) (i.GAD7_cat2) if Subject!=74
//No changes
xi: stepwise, pr(.2) pe(.05): logistic DL_SLSfailpass (i.div) (i.Age_cat) DL_ADF DL_Nm3BWm_100 DL_SI_1  DL_TLI_1 DL_IP_4W AFAQ PSS14 (i.PSQI_cat2) (i.GAD7_cat2) if Subject!=108
//DL_TLI_1 will be included in a n.s way
xi: stepwise, pr(.2) pe(.05): logistic DL_SLSfailpass (i.div) (i.Age_cat) DL_ADF DL_Nm3BWm_100 DL_SI_1  DL_TLI_1 DL_IP_4W AFAQ PSS14 (i.PSQI_cat2) (i.GAD7_cat2) if Subject!=155 & Subject!=258 & Subject!=12 & Subject!=144 & Subject!=108 & Subject!=74
//DL_TLI_1 will be included in a n.s way

//Conclusion: None of the subjects with large resiuduals had high leverage. //exclusion of some subjects due to hig leverage mostly include DL_TLI_1 in a n.s way. In two cases GAD7 will be included significantly. No further action is taken.
```

```
*THIRDLY, Model fit
xi: stepwise, pr(.2) pe(.05): logistic DL_SLSfailpass (i.div) (i.Age_cat) DL_ADF DL_Nm3BWm_100
DL_SI_1 DL_TLI_1 DL_IP_4W AFAQ PSS14 (i.PSQI_cat2) (i.GAD7_cat2)
estat gof, group(10) //p=0.97
linktest //_hat: p=0.03, _hatsq: p=0.90



//Conclusion: this is the final model



*******************************************************************************************
*KNEE MODEL FOR THE DOMINANT AND NON-DOMINANT LEG
*******************************************************************************************

*CATEGORISATION OF SOME VARIABLES:

//Variable GAD-7:
//already defined:gen GAD7_cat=.
//already defined:replace GAD7_cat=0 if GAD7<5
//already defined:replace GAD7_cat=1 if GAD7>=5 & GAD7<10
//already defined:replace GAD7_cat=2 if GAD7>=10 & GAD7<15
//already defined:replace GAD7_cat=3 if GAD7>=15 & GAD7<.
//already defined:label define GAD7_label 0 "No GAD" 1 "Mild GAD" 2 "Moderate GAD" 3 "Severe
GAD"
//already defined:label values GAD7_cat GAD7_label

//Variable GAD-7:
gen GAD7_cat2=.
replace GAD7_cat2=0 if GAD7<5
replace GAD7_cat2=1 if GAD7>=5 & GAD7<10
replace GAD7_cat2=2 if GAD7>=10 & GAD7<.
label define GAD7_label 0 "No GAD" 1 "Mild GAD" 2 "Moderate/Severe GAD"
label values GAD7_cat2 GAD7_label

//Variable div_cat
//already defined:gen div_cat=.
//already defined:replace div_cat=1 if div<2
//already defined:replace div_cat=2 if div>=2 & div<3
//already defined:replace div_cat=3 if div>=3 & div<.
//already defined:label define div_label 1 "Damallasvenskan" 2 "Elitettan" 3 "Div 1"
//already defined:label values div_cat div_label

//variable Age_cat
sum Age, detail //for the whole material: Q1=19, Q2=22 and Q3=25
gen Age_cat=.
replace Age_cat=1 if Age<20
replace Age_cat=2 if Age>=20 & Age<25
replace Age_cat=3 if Age>=25 & Age<.
label define Age_label 1 "<20" 2 "20-24" 3 ">24"
label values Age_cat Age_label


//Variable PSQI_cat:
```

```
sum PSQI, detail //Q1=3, Q2=5 and Q3=7
gen PSQI_cat=.
replace PSQI_cat=0 if PSQI <3
replace PSQI_cat=1 if PSQI >=3 & PSQI<5
replace PSQI_cat=2 if PSQI >=5 & PSQI<7
replace PSQI_cat=3 if PSQI >=7 & PSQI<.

label define PSQI_cat_label 0 "<3" 1 ">=3 to <5" 2 ">=5 to <7" 3">=7"
label values PSQI_cat PSQI_cat_label


//Variable PSQI_cat2:
sum PSQI, detail //According to the PSQI scale the cut off >5 can be used to distinguish
between good and poor sleepers
gen PSQI_cat2=.
replace PSQI_cat2=0 if PSQI <=5
replace PSQI_cat2=1 if PSQI >=6 & PSQI<.
label define PSQI_cat2_label 0 "<=5" 1 ">5"
label values PSQI_cat2 PSQI_cat2_label

//Variabeln PSS14
sum PSS14, detail // Q1=29.5, Q2=32, Q3=34

gen PSS14_cat=.
replace PSS14_cat=0 if PSS14 <29.5
replace PSS14_cat=1 if PSS14 >=29.5 & PSS14<32
replace PSS14_cat=2 if PSS14 >=32 & PSS14 <34
replace PSS14_cat=3 if PSS14 >=34 & PSS14<.

label define PSS14_cat_label 0 "<29.5" 1 ">=29.5 & PSS14<32" 2 ">=32 & PSS14 <34" 3 ">=34 &
PSS14<."
label values PSS14_cat PSS14_cat_label


//Variabeln PSS14
tab PSS14 // 0-29=25%, 30-32=23%, 32-34=33%, >34=19%

gen PSS14_cat2=.
replace PSS14_cat2=0 if PSS14 <30
replace PSS14_cat2=1 if PSS14 >=30 & PSS14<32
replace PSS14_cat2=2 if PSS14 >=32 & PSS14<35
replace PSS14_cat2=3 if PSS14 >34 & PSS14<.

label define PSS14_cat2_label 0 "<30" 1 "30-31" 2 "32-34" 3 ">34"
label values PSS14_cat2 PSS14_cat2_label

//Variabeln PSS14 with ten categories
gen PSS14_cat3=.
replace PSS14_cat3=0 if PSS14 <28
replace PSS14_cat3=1 if PSS14 >=28 & PSS14<29
replace PSS14_cat3=2 if PSS14 >=29 & PSS14<30
replace PSS14_cat3=3 if PSS14 >=30 & PSS14<31
replace PSS14_cat3=4 if PSS14 >=31 & PSS14<32
replace PSS14_cat3=5 if PSS14 >=32 & PSS14<33
replace PSS14_cat3=6 if PSS14 >=33 & PSS14<34
replace PSS14_cat3=7 if PSS14 >=34 & PSS14<35
replace PSS14_cat3=8 if PSS14 >=35 & PSS14<36
replace PSS14_cat3=9 if PSS14 >=36 & PSS14<37
```

```
replace PSS14_cat3=10 if PSS14 >37 & PSS14<.


//Variabeln AFAQ
sum AFAQ, detail //Q1=19, Q2=23, Q3=28

gen AFAQ_cat=.
replace AFAQ_cat=0 if AFAQ <19
replace AFAQ_cat=1 if AFAQ >=19 & AFAQ<23
replace AFAQ_cat=2 if AFAQ >=23 & AFAQ<28
replace AFAQ_cat=3 if AFAQ >=28 & AFAQ<.

label define AFAQ_cat_label 0 "<19" 1 "AFAQ >=19 & AFAQ<23" 2 "AFAQ >=23 & AFAQ<28" 3 "AFAQ
>=28 & AFAQ<."
label values AFAQ_cat AFAQ_cat_label


//Variabeln AFAQ with 4 categories
gen AFAQ_cat2=.
replace AFAQ_cat2=0 if AFAQ <19
replace AFAQ_cat2=1 if AFAQ >=19 & AFAQ<24
replace AFAQ_cat2=2 if AFAQ >=24 & AFAQ<28
replace AFAQ_cat2=3 if AFAQ >=28 & AFAQ<.

label define AFAQ_cat2_label 0 "<19" 1 "19-23" 2 "24-28" 3 "AFAQ >=28"
label values AFAQ_cat2 AFAQ_cat2_label


//Variabeln AFAQ with 9 categories
gen AFAQ_cat3=.
replace AFAQ_cat3=0 if AFAQ <14
replace AFAQ_cat3=1 if AFAQ >=14 & AFAQ<17
replace AFAQ_cat3=2 if AFAQ >=17 & AFAQ<20
replace AFAQ_cat3=3 if AFAQ >=20 & AFAQ<22
replace AFAQ_cat3=4 if AFAQ >=22 & AFAQ<24
replace AFAQ_cat3=5 if AFAQ >=24 & AFAQ<26
replace AFAQ_cat3=6 if AFAQ >=26 & AFAQ<29
replace AFAQ_cat3=7 if AFAQ >=29 & AFAQ<33
replace AFAQ_cat3=8 if AFAQ >=33 & AFAQ<.

***********************************************************************************
*STATISTICS FOR TABLE 2 (just shown for hip strenght)
***********************************************************************************************
sum NDL_Nm3BWm_100 if div==1, detail
sum NDL_Nm3BWm_100 if div==2, detail
sum NDL_Nm3BWm_100 if div==3, detail
sum DL_Nm3BWm_100 if div==1, detail
sum DL_Nm3BWm_100 if div==2, detail
sum DL_Nm3BWm_100 if div==3, detail
```

```
******************************************************************************************
*MCNEMAR´S CHI2 TEST FOR PARIED DATA ON NOMINAL LEVEL TO INVESTIGATE IF THERE IS ANY
SIGNIFICANT DIFFERENCE IN THE OUTCOME WHEN PERFORMING A SLS TEST ON THE DOMINANT OR NON-
DOMINANT LEG
******************************************************************************************
tab NDL_SLSknee DL_SLSknee
mcci 132 52 20 50 //p=0.0002 (McNemar's chi2(1) is a paried probabilty test on nominal data
level)
mcc NDL_SLSknee DL_SLSknee //p=0.002 (McNemar's chi2(1)


******************************************************************************************
*WILCOXON SIGNED RANK TEST FOR PAIRED (DEPENDENT)NON-NORMALLY DISTRIBUTED DATA TO INVESTIGATE
THE DIFFERENCES BETWEEN STRENGTH AND ANKLE DORSIFLEXION IN THE DOMINANT AND NON-DOMINANT LEG
******************************************************************************************

signrank NDL_Nm3BWm_100=DL_Nm3BWm_100 //p=0.0260 (Wilcoxon signed-rank test Is a paired test
for non normaly distributed data)
signrank NDL_ADF=DL_ADF //p=0.1091 (Wilcoxon signed-rank test Is a paired test for non normaly
distributed data)
ttest NDL_ADF=DL_ADF //p=0.1333 (paired t test: this variable is normally distributed and
could use t test statistics)



******************************************************************************************
*WILCOXON RANK SUM TEST FOR UNPAIRED (INDEPENDENT) NON-NORMALLY DISTRIBUTED DATA TO
INVESTIGATE THE DIFFERENCE IN STRENGTH AND ADF BETWEEN THOSE WHO FAILED AND PASSED THE SLS
******************************************************************************************
ranksum NDL_Nm3BWm_100, by(NDL_SLSknee) //p=0.0058 (Wilcoxon rank sum test is an unpaired test
for non-normally distributed data)
ttest NDL_Nm3BWm_100, by(NDL_SLSknee) //p=0.033
ranksum DL_Nm3BWm_100, by(DL_SLSknee) //p=0.3234
ttest DL_Nm3BWm_100, by(DL_SLSknee) //p=0.26

ranksum NDL_ADF, by(NDL_SLSknee) //p=0.86
ranksum DL_ADF, by(DL_SLSknee) //p=0.11
ttest NDL_ADF, by(NDL_SLSknee) //p=0.89 (this variable is normally distributed and could use t
test statistics)
ttest NDL_ADF, by(NDL_SLSknee) unequal //p=0.89 (The assumption of equal variances can be
optionally relaxed in the unpaired two-sample case.)
ttest DL_ADF, by(DL_SLSknee) //p=0.11
ttest DL_ADF, by(DL_SLSknee) unequal //p=0.11

******************************************************************************************
*ANOVA AND SIMPLE REGRESSION ANALYSIS TO INVESTIGATE THE DIFFERENCES BETWEEN STRENGTH AND
ANKLE DORSIFLEXION IN THE DOMINANT AND NON-DOMINANT LEG FOR DIFFERENT DIVISIONS
******************************************************************************************
oneway NDL_Nm3BWm_100 div //n.s
oneway NDL_Nm3BWm_100 div, bonferroni //n.s
reg NDL_Nm3BWm_100 i.div //n.s
oneway DL_Nm3BWm_100 div //n.s
oneway DL_Nm3BWm_100 div, bonferroni //n.s
reg DL_Nm3BWm_100 i.div //n.s

oneway NDL_ADF div //n.s
reg NDL_ADF i.div //n.s
oneway DL_ADF div //n.s
```

```
reg DL_ADF i.div //n.s

*As the variables above might not be perfectly normally distrbuted median regression is also performed

qreg NDL_Nm3BWm_100 i.div  //n.s
qreg DL_Nm3BWm_100 i.div //div 2 p=0.04
qreg NDL_ADF i.div //n.s
qreg DL_ADF i.div //n.s

*********************************************************************************************
*MODEL BUILDING WITH THE COMMAND STEPWISE, A BACKWARD LOGISTIC REGRESSION MODEL FOR THE KNEE: DOMINANT LEG
*********************************************************************************************
```

*** STEP 1.**

```
//Looking at univariable logistic regression model for all possible covariate (see table 2) and evaluating the
//variables with descriptive statistics (i.e Outliers, normal distribution)


//The variable division (Premier league, elitettan and division 1)
logistic DL_SLSknee div
logistic DL_SLSknee i.div
tab DL_SLSknee div


//The variable Age
logistic DL_SLSknee Age
sum Age if DL_SLSknee==0, detail
sum Age if DL_SLSknee==1, detail
graph box Age, over (DL_SLSknee) //Two outliers
scatter Age Subject, mlabel(Subject)
list Subject DL_SLSknee Age
logistic DL_SLSknee Age if Subject!=76 & Subject !=143 // Conclusion: No significant change in OR, CI, Std error or P when the outliers are excluded.

histogram Age, normal //not normally distributed
symplot Age // skewed to the right
qnorm Age, grid //focus on the tails, fairly normally distributed
pnorm Age, grid //Focus on the middle of the distribution, fairly normally distributed
sktest Age // P<0.05, thus we can reject the hypothesis that Age is normally distributed
tabstat Age, stat(n mean sd p25 p50 p75)
di22.27559-22//Mean-Median = .27559
display 25-22 //Upper quartile-median= 3
display 22-19 //median-lower quartile= 3


//Conlusion: The variable Age is not normally distributed



//The variable Age_cat
logistic DL_SLSknee Age_cat
logistic DL_SLSknee i.Age_cat
tab DL_SLSknee Age_cat
```

```
//The variable DL_ADF (Ankel dorsiflexion)
logistic DL_SLSknee DL_ADF
sum DL_ADF if DL_SLSknee==0, detail
sum DL_ADF if DL_SLSknee==1, detail
graph box DL_ADF, over (DL_SLSknee) // Four outliers,
scatter DL_ADF Subject, mlabel (Subject)
list Subject DL_SLSknee DL_ADF
logistic DL_SLSknee DL_ADF if Subject!=109 & Subject !=6 & Subject!=258 & Subject!=217 //No
significant change in OR, CI, Std error or P when the outliers are excluded

histogram DL_ADF, normal // Looks relatively normally distributed
symplot DL_ADF // Skewed to the left
qnorm DL_ADF, grid //focus on the tails, looks normally distributed
pnorm DL_ADF, grid //Focus on the middle of the distribution, looks normally distributed
sktest DL_ADF // p=0.35, We cannot reject the hypothesis that DL_ADF is normally distributed
tabstat DL_ADF, stat(n mean sd p25 p50 p75)
di 44.76395 -44.96423 //Mean-Median = -.20028
di 47.33566-44.96423  //Upper quartile-median = 2.37143
di 44.96423 -42.23914  //median-lower quartile 2.72509



//Conclusion: The variable DL_ADF are normally distributed but a little bit skewd to the left



//The variable DL_Nm3BWm_100 (Hip strenght/CLAM)
logistic DL_SLSknee DL_Nm3BWm_100
sum DL_Nm3BWm_100 if DL_SLSknee==0, detail
sum DL_Nm3BWm_100 if DL_SLSknee==1, detail
graph box DL_Nm3BWm_100, over (DL_SLSknee ) //Four outliers
scatter DL_Nm3BWm_100 Subject, mlabel (Subject)
list Subject DL_SLSknee DL_Nm3BWm_100
logistic DL_SLSknee DL_Nm3BWm_100 if Subject!=261 & Subject!=245 & Subject!=226 & Subject!=90
di .9936028 - .9941656 /.9936028    // OR decreased 0.7%, no greater change in CI and Std Err.

histogram DL_Nm3BWm_100, normal // Looks skewed to the right
symplot DL_Nm3BWm_100 // Skewed to the right (points lying over the reference line indicate
skewness to the right)
qnorm DL_Nm3BWm_100, grid //focus on the tails, looks fairly normally distributed
pnorm DL_Nm3BWm_100, grid //Focus on the middle of the distribution, looks normally
distributed
sktest DL_Nm3BWm_100 //P<0.05, thus we can reject the hypothesis that DL_Nm3BWm_100 is
normally distributed
tabstat DL_Nm3BWm_100, stat(n mean sd p25 p50 p75)
di 96.79302 - 95.75 //Mean-Median = 1.04
di 110.905-95.75  //Upper quartile-median = 15.16
di 95.75-79.295  //median-lower quartile= 16.46



              //Conclusion: this variable is not normally distributed


tabstat NDL_Nm3BWm_100, stat(n mean sd p25 p50 p75)
```

```
// The variable Serious injury, Serious knee injury, Time loss injury within the last 4 weeks and Injury problems within the last 4 weeks


logistic DL_SLSknee DL_SI_1
tab DL_SLSknee DL_SI_1

logistic DL_SLSknee DL_SI_knee_noknee
tab DL_SLSknee DL_SI_knee_noknee //due to low cell count for the non-dominant leg this variable is taken away.

logistic DL_SLSknee DL_TLI_4W
tab DL_SLSknee DL_TLI_4W

logistic DL_SLSknee DL_IP_4W
tab DL_SLSknee DL_IP_4W


//The variable AFAQ (Fear of avoidance Questionnaire, range 10-50 points)
logistic DL_SLSknee AFAQ
sum AFAQ if DL_SLSknee==0, detail
sum AFAQ if DL_SLSknee==1, detail
graph box AFAQ, over( DL_SLSknee ) //Two outliers
scatter AFAQ Subject, mlabel (Subject)
list Subject DL_SLSknee AFAQ
logistic DL_SLSknee AFAQ if Subject!=80 & Subject!=43 //No significant change in OR, CI, Std error or P when the outliers are excluded.

histogram AFAQ, normal // Skewed to the right
symplot AFAQ // Skewed to the right
qnorm AFAQ, grid //focus on the tails, looks fairly normally distributed
pnorm AFAQ, grid //Focus on the middle of the distribution, looks fairly normally distributed
sktest AFAQ //P>0.05, thus we cannot reject the hypothesis that AFAQ is normally distributed
tabstat AFAQ, stat(n mean sd p25 p50 p75)
di  23.56746 -23 //Mean-Median = .56746
di 28-23 //Upper quartile-median = 5
di 23-19 //median-lower quartile= 4

//Conclusion: the variable AFAQ are fairly normally distributed but a little bit //skewed to the right


//The variable PSS14 (Percieved stress scale-14, range 0-56 points)
logistic DL_SLSknee PSS14
sum PSS14 if DL_SLSknee==0, detail
sum PSS14 if DL_SLSknee==1, detail
graph box PSS14, over (DL_SLSknee ) //Four outliers
scatter PSS14  Subject, mlabel (Subject)
list Subject DL_SLSknee PSS14
logistic DL_SLSknee PSS14 if Subject!=46 & Subject!=52 & Subject!=63 & Subject!=189 //****The variable PSS14 goes from significant P=0.049 to non significant p=0.089
```

```
histogram PSS14, normal // Fairly normally distributed but a little bit Skewed to the left
symplot PSS14 // Skewed to the left
qnorm PSS14, grid //focus on the tails, does not look normally distributed
pnorm PSS14, grid //Focus on the middle of the distribution, does not look normally
distributed
sktest PSS14 //P>0.05, thus we cannot reject the hypothesis that PSS14 is normally distributed
tabstat PSS14, stat(n mean sd p25 p50 p75)
di 31.74206 -32 //Mean-Median = -.25794
di 34-32 //Upper quartile-median = 2
di 32-29.5 //median-lower quartile= 2.5



//Conclusion: Divergent results, the variable PSS14 will not be treated as normally
distributed.


//The variable PSQI (Pittsburgh Sleep Quality Inventory, range 0-21 points)
logistic DL_SLSknee PSQI
sum PSQI if DL_SLSknee==0, detail
sum PSQI if DL_SLSknee==1, detail
graph box PSQI, over (DL_SLSknee) // Four outliers
scatter PSQI  Subject, mlabel (Subject)
list Subject DL_SLSknee PSQI
logistic DL_SLSknee PSQI if Subject!=92 & Subject!=225 & Subject!=173 & Subject!=246
//******The variable PSQI goes from Non significant 0.19 to //significant P=0.03



histogram PSQI, normal // Skewed to the right
symplot PSQI // Skewed to the right
qnorm PSQI, grid //focus on the tails, not ok
pnorm PSQI, grid //Focus on the middle of the distribution, not ok
sktest PSQI //P<0.05, thus we can reject the hypothesis that PSQI is normally distributed
tabstat PSQI, stat(n mean sd p25 p50 p75)
di 5.198413-5 //Mean-Median = .198413
di 7-5 //Upper quartile-median = 2
di 5-3 //median-lower quartile= 2

//Conclusion: the variable PSQI is not normally distributed


logistic DL_SLSknee PSQI_cat2
logistic DL_SLSknee i.PSQI_cat2
tab DL_SLSknee PSQI_cat2


//The variable GAD-7 (General Anxiety Disorder, range 0-21)
logistic DL_SLSknee GAD7
sum GAD7 if DL_SLSknee==0, detail
sum GAD7 if DL_SLSknee==1, detail
graph box GAD7, over (DL_SLSknee) // Six outliers
scatter GAD7 Subject, mlabel (Subject)
list Subject DL_SLSknee GAD7
logistic DL_SLSknee GAD7 if Subject!=45 & Subject!=31 & Subject!=7 & Subject!=230 &
Subject!=75 & Subject!=111
//No significant change in OR, CI, Std error or P when the outliers are excluded.
```

```
histogram GAD7, normal // Skewed to the right and not normally distributed
symplot GAD7 // Skewed to the right
qnorm GAD7, grid //focus on the tails, not ok
pnorm GAD7, grid //Focus on the middle of the distribution, not ok
sktest GAD7 //P<0.05, thus we can reject the hypothesis that GAD/ is normally distributed
tabstat GAD7, stat(n mean sd p25 p50 p75)
di 6.115079-5 //Mean-Median = 1.115079
di 8-5 //Upper quartile-median = 3
di 5-3 //median-lower quartile= 2


                                        //The variable GAD7 is not normally distributed


logistic DL_SLSknee GAD7_cat2
logistic DL_SLSknee i.GAD7_cat2
tab DL_SLSknee GAD7_cat2
```

***STEP 2: CHECKING FOR COLLINEARITY AND LINEAR ASSUMPTIONS**

```
*Check for collinearity between the continous independent variables; This will be checked in the stepwise command

*Check for correlation between the dichotomous independent variables;
corr DL_SI_1 DL_SI_knee_noknee DL_TLI_4W DL_IP_4W
spearman DL_SI_1 DL_SI_knee_noknee DL_TLI_4W DL_IP_4W //there is some correlation between DL_SI_1 DL_SI_knee_noknee but not the other variables

reg DL_SI_1 DL_SI_knee_noknee //reg=0.73
corr DL_SI_1 DL_SI_knee_noknee //corr=0.60
spearman DL_SI_1 DL_SI_knee_noknee //spearman=0.60
kap DL_SI_1 DL_SI_knee_noknee //kap=0.58

//conclusion: There is a high correlation between DL_SI_1 and DL_SI_knee_noknee, the two variables should not //be analysed together in a model. The variable DL_SI_1 is used due to few cell count for the variable  DL_SI_knee_noknee//in the non-dominant leg.

reg GAD7_cat2 PSQI_cat2    //reg=0.48

corr GAD7_cat2 PSQI_cat2 //corr=0.32
spearman GAD7_cat2 PSQI_cat2 //spearman=0.31
kap GAD7_cat2 PSQI_cat2    //kap=0.15
//No correlation between GAD and PSQI


*Test the assumptions for liniarity for each continuous variable in the model; thus that the log odds of the outcome and independet variable hava a linear relationship

//FIRSTLY, linearity is checked with plots:

logistic DL_SLSknee i.div Age DL_ADF DL_Nm3BWm_100 DL_SI_1 DL_TLI_4W DL_IP_4W AFAQ PSS14 PSQI i.GAD7_cat2
predict logits_DL, xb

scatter logits_DL Age || lowess logits_DL Age //Does not look linear
scatter logits_DL DL_ADF || lowess logits_DL DL_ADF //Looks ok
```

```
scatter logits_DL DL_Nm3BWm_100 || lowess logits_DL DL_Nm3BWm_100 //Looks ok
scatter logits_DL AFAQ || lowess logits_DL AFAQ //Looks ok, but have a tendency to be flatened
out or U-shaped
scatter logits_DL PSS14 || lowess logits_DL PSS14 //Looks ok,
scatter logits_DL PSQI || lowess logits_DL PSQI //Does not look linear




//SECONDLY, the boxtid test is used to check if the variables fullfills the assumption of
linearity. If P>0.05 the assumption is fulfilled:
boxtid logit DL_SLSknee Age DL_ADF DL_Nm3BWm_100 AFAQ PSS14 PSQI //Age: p=0.029, DL_ADF:
p=0.90, DL_Nm3BWm_100: p=0.65, AFAQ: p=0.55, PSS14: p=0.29 and PSQI: p=0.005.
// Age and PSQI does not fulfill the assumption of linearity.

//In order to get linearity for Age and PSQI:

//Age: Checks if there is any difference when outliers are excluded. No differences, p<0.05.
See which are outliers in step 1.
boxtid logit DL_SLSknee Age_01

//Age: Transformation
gen Age_sqrt= sqrt(Age)
boxtid logit DL_SLSknee Age_sqrt //p=0.011
gen Age_sq=Age^2
boxtid logit DL_SLSknee Age_sq //p=0.0006
gen Age_log=log(Age)
boxtid logit DL_SLSknee Age_log //p=0.015



//Age:Visual check of linearity
logistic DL_SLSknee i.div Age DL_ADF DL_Nm3BWm_100 DL_SI_1 DL_TLI_4W DL_IP_4W AFAQ PSS14 PSQI
i.GAD7_cat2 Age_sqrt Age_sq Age_log
predict logits_DL_Age, xb
scatter logits_DL_Age Age_sqrt || lowess logits_DL_Age Age_sqrt
scatter logits_DL_Age Age_sq || lowess logits_DL_Age Age_sq
scatter logits_DL_Age Age_log || lowess logits_DL_Age Age_log


//The variable Age will be categorised instead of treated as a continous variable


//PSQI:Checks if there is any difference when outliers are excluded. No differences, p<0.05.
See which are outliers in step 1.
boxtid logit DL_SLSknee PSQI_01

//PSQI:Transformation
gen PSQI_sqrt= sqrt(PSQI) //Square root transformation in order to get linearity
boxtid logit DL_SLSknee PSQI_sqrt //p=0.03
gen PSQI_sq=PSQI^2
boxtid logit DL_SLSknee PSQI_sq //p=0.03
gen PSQI_log=log(PSQI)
boxtid logit DL_SLSknee PSQI_log //p=0.45

//PSQI: Visual check of linearity
logistic DL_SLSknee i.div Age DL_ADF DL_Nm3BWm DL_SI_1 DL_TLI_4W DL_IP_4W AFAQ PSS14 PSQI_log
i.GAD7_cat2
```

```
predict logits_DL_PSQI,xb
scatter logits_DL_PSQI PSQI_log || lowess logits_DL_PSQI PSQI_log


//PSQI could be seen as linear but it has a flat step in the beginning
//General conclusion of linearity: Age does not fulfil the assumption of linearity even if
//the variables is transformed in three ways, the variable will be categorized instead.
//PSQI_log fulfills the assumption for the boxtid test and have an ok linear relation on
visual inspection, //As we have a good reference with a cut off value for PSQI we descided to
categorize the variable instead.
```

***STEP 3: STEPWISE LOGISTISTIC REGRESSION**

```
//stepwise logistic without excluding outliers
//A good link to linktest:
https://stats.oarc.ucla.edu/stata/webbooks/logistic/chapter3/lesson-3-logistic-regression-
diagnostics/
xi: stepwise, pr(.2) pe(.05): logistic DL_SLSknee (i.div) (i.Age_cat) DL_ADF DL_Nm3BWm_100
DL_SI_1 DL_TLI_4W DL_IP_4W AFAQ PSS14 (i.PSQI_cat2) (i.GAD7_cat2)
estat gof, group(10)
linktest //https://stats.oarc.ucla.edu/stata/webbooks/logistic/chapter3/lesson-3-logistic-
regression-diagnostics/


//PSS-14:Exclusion of outliers where the variable PSS14 goes from significant P=0.049 to non
significant p=0.089 (see step 1)
replace PSS14_01 = . in 46
replace PSS14_01 = . in 52
replace PSS14_01 = . in 62
replace PSS14_01 = . in 185

xi: stepwise, pr(.2) pe(.05): logistic DL_SLSknee (i.div) (i.Age_cat) DL_ADF DL_Nm3BWm_100
DL_SI_1 DL_TLI_4W DL_IP_4W AFAQ PSS14 (i.PSQI_cat2) (i.GAD7_cat2)
estat gof, group(10)
linktest


xi: stepwise, pr(.2) pe(.05): logistic DL_SLSknee (i.div) (i.Age_cat) DL_ADF DL_Nm3BWm_100
DL_SI_1 DL_TLI_4W DL_IP_4W AFAQ PSS14_01 (i.PSQI_cat2) (i.GAD7_cat2)
estat gof, group(10)
linktest

clist if Subject==46 | Subject==52 | Subject==62 | Subject==185


//Conclusion: The two models with and without the outliers in PSS14 are similar. As there
doesn´t seem to //be any flaws in the registration of the outliers those will be used in the
variable PSS-14.

//PSQI: Exclusion of outliers where the variable PSQI goes from Non significant 0.19 to
significant P=0.03 (see step 1)
//In the variable PSQI_02_01 the the PSQI values for Subjects 92, 173, 225 and 246
              are deleted.

xi: stepwise, pr(.2) pe(.05): logistic DL_SLSknee (i.div) (i.Age_cat) DL_ADF DL_Nm3BWm_100
DL_SI_1 DL_TLI_4W DL_IP_4W AFAQ PSS14 (i.PSQI_cat2) (i.GAD7_cat2)
estat gof, group(10)
linktest
```

```
xi: stepwise, pr(.2) pe(.05): logistic DL_SLSknee (i.div) (i.Age_cat) DL_ADF DL_Nm3BWm_100
DL_SI_1 DL_TLI_4W DL_IP_4W AFAQ PSS14_01 (PSQI_cat2_01) (i.GAD7_cat2)
estat gof, group(10)
linktest

clist if Subject==92 | Subject==225 | Subject==173 | Subject==246

//Conclusion: The two models with and without the outliers in PSQI_cat are not similar. div_2
goes from significant to n.s.
//As there is no flaws in the registration of the outliers those will be used in the variable
PSQI.
```

***STEP 4: CHECKING FOR INTERACTION AMONG THE VARIABLES THAT ARE LEFT IN THE FINAL MODEL FROM ABOVE**

```
//At this stage the variable DL_SI_knee_noknee is taken away as it only has 3 outcome in on
category (se table 2), it is also highly correlated to DL_SI_1
xi: stepwise, pr(.2) pe(.05): logistic DL_SLSknee (i.div) (i.Age_cat) DL_ADF DL_Nm3BWm_100
DL_SI_1 DL_TLI_4W DL_IP_4W AFAQ PSS14 (i.PSQI_cat2) (i.GAD7_cat2)
//included variables in the final model are: div, PSS14, DL_ADF, GAD7


//div vs PSS14
gen div_PSS14=div*PSS14
logistic DL_SLSknee div PSS14 div_PSS14 //p=0.86

//div vs GAD7_cat2
gen div_GAD7_cat2=div*GAD7_cat2
logistic DL_SLSknee div GAD7_cat2 div_GAD7_cat2 //p=0.93


//GAD7_cat2 vs PSS14
gen GAD7_cat2_PSS14=GAD7_cat2* PSS14
logistic DL_SLSknee GAD7_cat2 PSS14 GAD7_cat2_PSS14 //p=0.75


              //Conclusion: No interaction found
```

***STEP 5: CHECKING FOR MODEL ASSUMPTIONS AND FIT**

```
xi: stepwise, pr(.2) pe(.05): logistic DL_SLSknee (i.div) (i.Age_cat) DL_ADF DL_Nm3BWm_100
DL_SI_1 DL_TLI_4W DL_IP_4W AFAQ PSS14 (i.PSQI_cat2) (i.GAD7_cat2)

*FIRSTLY, Check for collinearity; This will be checked in the stepwise command but could also
be checked like this:
regress DL_ADF DL_Nm3BWm_100 AFAQ PSS14
vif // If vif =>5 there is coliniarity. In this test vif=1.03
collin DL_ADF DL_Nm3BWm_100 AFAQ PSS14 //If the variables are non-correlated both vif and
tolerance

//should be close to 1. In this test both tolerance and vif is close to 1.0
```

```
*SECONDLY, checking for outlying, high leverage and influential points
//Starts by looking at residual and leverage plots
//A rule of thumb if the sample size is high, a point is influential if the Pearson and
Deviance residual is >2 and that the leverage hat value is >2-3 times
//higher than the average of the leverage (ref:
https://stats.oarc.ucla.edu/stata/webbooks/logistic/chapter3/lesson-3-logistic-regression-
diagnostics-2/)

xi: stepwise, pr(.2) pe(.05): logistic DL_SLSknee (i.div) (i.Age_cat) DL_ADF DL_Nm3BWm_100
DL_SI_1 DL_TLI_4W DL_IP_4W AFAQ PSS14 (i.PSQI_cat2) (i.GAD7_cat2)
logistic DL_SLSknee i.div i.Age_cat DL_ADF DL_Nm3BWm_100 DL_SI_1 DL_TLI_4W DL_IP_4W AFAQ PSS14
i.PSQI_cat2 i.GAD7_cat2

predict phat_DL, p
predict rstandard_DL, rstandard
scatter rstandard_DL phat_DL, mlabel(Subject) //Produces a plot for the standardized Pearson
residuals: Subject 239, 233 and 145 have a residual over 2.
scatter rstandard_DL Subject, mlabel(Subject) //Produces an indexplot for the standardized
Person residuals: Subjects 239, 233, 145, 123, 40 and 11 has a residual >2
predict dv_DL, dev
scatter dv_DL phat_DL, mlab(Subject) //Produces a deviance residual plot, another type of plot
that should give the same results as the Pearson residuals:
                                     //looks the same for 239 and 268.

predict hat_DL, hat
scatter hat_DL phat_DL, mlab(Subject) //Produces a leverage plot: The leverage mean is
approximately 0.06 (0.15/0.06=2.5) so values around 0.15
                                      //will be seen as high leverage. Observation  239, 233,
145, 123, 40 and 11 with large residuals have low leverage.

                                           //Observation 246, 258, 155, 240, 105 and 74  has
the highest leverage
scatter hat_DL Subject, mlab(Subject) //Produces a leverage indexplot. Observation  239, 233,
145, 123, 40 and 11 with large residuals have low leverage.

                                          //Observation 246, 258, 155, 240, 105 and 74  has
the highest leverage


clist if Subject==246 | Subject==258 | Subject==155 | Subject==240 | Subject==105 |
Subject==74
//Looking at the values for all the different variables for subject 246, 258, 155, 240, 105
and 74. As Subject 239, 233, 145, 123, 40 and 11 with
//large residuals have low leverage; those will stay in the model. Does not find any errors in
the registration of subject 246, 258, 155, 240, 105 and 74.



//Comparing the models with and without Subject 67 and 253
xi: stepwise, pr(.2) pe(.05): logistic DL_SLSknee (i.div) (i.Age_cat) DL_ADF DL_Nm3BWm_100
DL_SI_1 DL_TLI_4W DL_IP_4W AFAQ PSS14 (i.PSQI_cat2) (i.GAD7_cat2)
xi: stepwise, pr(.2) pe(.05): logistic DL_SLSknee (i.div) (i.Age_cat) DL_ADF DL_Nm3BWm_100
DL_SI_1 DL_TLI_4W DL_IP_4W AFAQ PSS14 (i.PSQI_cat2) (i.GAD7_cat2) if Subject!=155
xi: stepwise, pr(.2) pe(.05): logistic DL_SLSknee (i.div) (i.Age_cat) DL_ADF DL_Nm3BWm_100
DL_SI_1 DL_TLI_4W DL_IP_4W AFAQ PSS14 (i.PSQI_cat2) (i.GAD7_cat2) if Subject!=246
```

```
xi: stepwise, pr(.2) pe(.05): logistic DL_SLSknee (i.div) (i.Age_cat) DL_ADF DL_Nm3BWm_100
DL_SI_1 DL_TLI_4W DL_IP_4W AFAQ PSS14 (i.PSQI_cat2) (i.GAD7_cat2) if Subject!=240
xi: stepwise, pr(.2) pe(.05): logistic DL_SLSknee (i.div) (i.Age_cat) DL_ADF DL_Nm3BWm_100
DL_SI_1 DL_TLI_4W DL_IP_4W AFAQ PSS14 (i.PSQI_cat2) (i.GAD7_cat2) if Subject!=258
xi: stepwise, pr(.2) pe(.05): logistic DL_SLSknee (i.div) (i.Age_cat) DL_ADF DL_Nm3BWm_100
DL_SI_1 DL_TLI_4W DL_IP_4W AFAQ PSS14 (i.PSQI_cat2) (i.GAD7_cat2) if Subject!=105
xi: stepwise, pr(.2) pe(.05): logistic DL_SLSknee (i.div) (i.Age_cat) DL_ADF DL_Nm3BWm_100
DL_SI_1 DL_TLI_4W DL_IP_4W AFAQ PSS14 (i.PSQI_cat2) (i.GAD7_cat2) if Subject!=74
xi: stepwise, pr(.2) pe(.05): logistic DL_SLSknee (i.div) (i.Age_cat) DL_ADF DL_Nm3BWm_100
DL_SI_1 DL_TLI_4W DL_IP_4W AFAQ PSS14 (i.PSQI_cat2) (i.GAD7_cat2) if Subject!=155 &
Subject!=246 & Subject!=240 & Subject!=258 & Subject!=105 & Subject!=74



             // Conclusion: Exclusion of Subjects with high leverage does not make any
greater change in significance, OR or direction.

             //No flaws are seen in the registration. No influential points found. The
subjects will stay in the model.



*THIRDLY, Model fit and test of final linearity
xi: stepwise, pr(.2) pe(.05): logistic DL_SLSknee (i.div) (i.Age_cat) DL_ADF DL_Nm3BWm_100
DL_SI_1 DL_TLI_4W DL_IP_4W AFAQ PSS14 (i.PSQI_cat2) (i.GAD7_cat2)
estat gof, group(10) table //the gof test is non-significant p=0.96.
//A significant finding indicates lack of fit and a non-significant result rules out a gross
lack of fit. Large p values does not always say that
//you have a good fit, just that there is no evidence enought to say that there is a bad fit.
The test is very sensitive to
//fairly small fit discrepancies in a larger sample. Thus, a significant results in such cases
may not signal a serious fit problem in such cases
linktest //The model has a reasonable fit if _hat is significant, the results in this model is
p=0.002. The model is adequate if _hatsq P>0.05, this model has p=0.85.
//A significant result for _hatq means that an alternativ binary regression model should be
considered (quadratic function, not using the logit),
//it may also indicate that important predictors have been omitted or are represented
incorrectly in the model


*Check of linearity in final model:
logistic DL_SLSknee i.div i.Age_cat DL_ADF DL_Nm3BWm_100 DL_SI_1 DL_TLI_4W DL_IP_4W AFAQ PSS14
i.PSQI_cat2 i.GAD7_cat2
predict logits_DL_final, xb
scatter logits_DL_final PSS14 || lowess logits_DL_final PSS14 //looks ok
scatter logits_DL_final DL_ADF || lowess logits_DL_final DL_ADF     //looks ok


//THIS IS THE FINAL MODEL//
```

```
*******************************************************************************************
*MODEL BUILDING WITH THE COMMAND STEPWISE, A BACKWARD LOGISTIC REGRESSION MODEL FOR THE KNEE:
NON-DOMINANT LEG
*******************************************************************************************



* STEP 1.
//Looking at univariable logistic regression model for all possible covariate (see table 2)
and evaluating the
//variables with descriptive statistics (i.e Outliers, normal distribution)


//The variable division (Premier league, elitettan and division 1)
logistic NDL_SLSknee div
logistic NDL_SLSknee i.div
tab NDL_SLSknee div


//The variable Age
logistic NDL_SLSknee Age
sum Age if NDL_SLSknee==0, detail
sum Age if NDL_SLSknee==1, detail
graph box Age, over( NDL_SLSknee ) //Two outliers
scatter Age Subject, mlabel(Subject)
list Subject NDL_SLSknee Age
logistic NDL_SLSknee Age if Subject!=76 & Subject !=143 // Conclusion: No significant change
in OR, CI, Std error or P when the outliers are excluded.

histogram Age, normal //not normally distributed
symplot Age // skewed to the right:
qnorm Age, grid //focus on the tails, fairly normally distributed
pnorm Age, grid //Focus on the middle of the distribution, fairly normally distributed
sktest Age // P<0.05, thus we can reject the hypothesis that Age is normally distributed
tabstat Age, stat(n mean sd p25 p50 p75)
display 22.36742 -22 //Mean-Median = .36742 (if skewed to the right/positive skeweness the
median is lower than the mean)
display 25-22 //Upper quartile-median= 3
display 22-19 //median-lower quartile= 3



 //Conlusion: The variable Age are not normally distributed


//The variable Age_cat
logistic NDL_SLSknee Age_cat
logistic NDL_SLSknee i.Age_cat
tab NDL_SLSknee Age_cat




//The variable NDL_ADF (Ankel dorsiflexion)
logistic NDL_SLSknee NDL_ADF
sum NDL_ADF if NDL_SLSknee==0, detail
sum NDL_ADF if NDL_SLSknee==1, detail
```

```
graph box NDL_ADF, over( NDL_SLSknee ) // Three outliers
scatter NDL_ADF Subject, mlabel (Subject)
list Subject NDL_SLSknee NDL_ADF
logistic NDL_SLSknee NDL_ADF if Subject!=109 & Subject !=251 & Subject !=217 //No significant
change in OR, CI, Std error or P when the outliers are excluded

histogram NDL_ADF, normal // Looks relatively normally distributed
symplot NDL_ADF // Looks fairly normally distributed
qnorm NDL_ADF, grid //focus on the tails, looks normally distributed
pnorm NDL_ADF, grid //Focus on the middle of the distribution, looks normally distributed
sktest NDL_ADF // p=0.92, We cannot reject the hypothesis that NDL_ADF is normally distributed
tabstat NDL_ADF, stat(n mean sd p25 p50 p75)
di 45.01126 - 44.85346 //Mean-Median =  .1578
di 47.8549-44.85346 //Upper quartile-median = 3.00144
di 44.85346 -42.36025 //median-lower quartile 2.49321



//Conclusion: The variable NDL_ADF is normally distributed



//The variable NDL_Nm3BWm_100 (Hip strenght/CLAM)
logistic NDL_SLSknee NDL_Nm3BWm_100
sum NDL_Nm3BWm_100 if NDL_SLSknee==0, detail
sum NDL_Nm3BWm_100 if NDL_SLSknee==1, detail
graph box NDL_Nm3BWm_100, over( NDL_SLSknee ) //One outlier
scatter NDL_Nm3BWm_100 Subject, mlabel (Subject)
list Subject NDL_SLSknee NDL_Nm3BWm_100
logistic NDL_SLSknee NDL_Nm3BWm_100 if Subject!=261 //Conclusion: No significant change in OR,
CI, Std error or P when the outliers are excluded.


histogram NDL_Nm3BWm_100, normal // Looks fairly normally distributed
symplot NDL_Nm3BWm_100 // Skewed to the right
qnorm NDL_Nm3BWm_100, grid //focus on the tails, looks fairly normally distributed
pnorm NDL_Nm3BWm_100, grid //Focus on the middle of the distribution, looks normally
distributed
sktest NDL_Nm3BWm_100 //P<0.05, thus we can reject the hypothesis that NDL_Nm3BWm_100 is
normally distributed
tabstat NDL_Nm3BWm_100, stat(n mean sd p25 p50 p75)
di 98.97194 -97.53  //Mean-Median =  1.44194
di 113.09 - 97.53  //Upper quartile-median = 15.56
di 97.53 -82.91  //median-lower quartile= 14.62

//Conclusion: the statistics points at no normal distributions, but the plots//does. Thus,
this variable will be counted as not normal distributed


// The variable Serious injury, Serious knee injury, Time loss injury within the last 4 weeks
and Injury problems within the last 4 weeks


logistic NDL_SLSknee NDL_SI_1
tab NDL_SLSknee NDL_SI_1

logistic NDL_SLSknee NDL_SI_knee_noknee
```

```
tab NDL_SLSknee NDL_SI_knee_noknee //due to low cell count for the non-dominant leg this
variable is taken away both for the dominant and non-dominant leg

logistic NDL_SLSknee NDL_TLI_4W
tab NDL_SLSknee NDL_TLI_4W

logistic NDL_SLSknee NDL_IP_4W
tab NDL_SLSknee NDL_IP_4W


//The variable AFAQ (Fear of avoidance Questionnaire, range 10-50 points)
logistic NDL_SLSknee AFAQ
sum AFAQ if NDL_SLSknee==0, detail
sum AFAQ if NDL_SLSknee==1, detail
graph box AFAQ, over( NDL_SLSknee ) //One outliers
scatter AFAQ Subject, mlabel (Subject)
list Subject NDL_SLSknee AFAQ
logistic NDL_SLSknee AFAQ if Subject!=80  //No significant change in OR, CI, Std error or P
when the outliers are excluded.

histogram AFAQ, normal // Skewed to the right
symplot AFAQ // Skewed to the right
qnorm AFAQ, grid //focus on the tails, looks fairly normally distributed
pnorm AFAQ, grid //Focus on the middle of the distribution, looks fairly normally distributed
sktest AFAQ //P>0.05, thus we cannot reject the hypothesis that AFAQ is normally distributed
tabstat AFAQ, stat(n mean sd p25 p50 p75)
di  23.56746 -23 //Mean-Median = .56746
di 28-23 //Upper quartile-median = 5
di 23-19 //median-lower quartile= 4


              //Conclusion: the variable AFAQ are fairly normally distributed but a little bit
              //skewed to the right




//The variable PSS14 (Percieved stress scale-14, range 0-56 points)
logistic NDL_SLSknee PSS14
sum PSS14 if NDL_SLSknee==0, detail
sum PSS14 if NDL_SLSknee==1, detail
graph box PSS14, over (NDL_SLSknee ) //Three outliers
scatter PSS14  Subject, mlabel (Subject)
list Subject NDL_SLSknee PSS14
logistic NDL_SLSknee PSS14 if Subject!=46 & Subject!=190 & Subject!=189  //No significant
change in OR, CI, Std error or P when the outliers are excluded.

histogram PSS14, normal // Fairly normally distributed but a little bit Skewed to the left
symplot PSS14 // Skewed to the left
qnorm PSS14, grid //focus on the tails, does not look normally distributed
pnorm PSS14, grid //Focus on the middle of the distribution, does not look normally
distributed
sktest PSS14 //P>0.05, thus we cannot reject the hypothesis that PSS14 is normally distributed
tabstat PSS14, stat(n mean sd p25 p50 p75)
di 31.74206 -32 //Mean-Median = -.25794 (if skewed to the left/negative skeweness the median
is higher than the mean)
di 34-32 //Upper quartile-median = 2
di 32-29.5 //median-lower quartile= 2.5
```

```
//Conclusion: Divergent results, the variable PSS14 will not be treated as normally
distributed.


//The variable PSQI (Pittsburgh Sleep Quality Inventory, range 0-21 points)
logistic NDL_SLSknee PSQI
sum PSQI if NDL_SLSknee==0, detail
sum PSQI if NDL_SLSknee==1, detail
graph box PSQI, over (NDL_SLSknee) // Four outliers
scatter PSQI  Subject, mlabel (Subject)
list Subject NDL_SLSknee PSQI
logistic NDL_SLSknee PSQI if Subject!=92 & Subject!=225 & Subject!=173 & Subject!=246 //No
significant change in OR, CI, Std error or P when the outliers are excluded.

histogram PSQI, normal // Skewed to the right
symplot PSQI // Skewed to the right
qnorm PSQI, grid //focus on the tails, not ok
pnorm PSQI, grid //Focus on the middle of the distribution, not ok
sktest PSQI //P<0.05, thus we can reject the hypothesis that PSQI is normally distributed
tabstat PSQI, stat(n mean sd p25 p50 p75)
di 5.198413-5 //Mean-Median = .198413
di 7-5 //Upper quartile-median = 2
di 5-3 //median-lower quartile= 2

              //Conclusion: The variable PSQI is not normally distributed


logistic NDL_SLSknee PSQI_cat2
logistic NDL_SLSknee i.PSQI_cat2
tab NDL_SLSknee PSQI_cat2



//The variable GAD-7 (General Anxiety Disorder, range 0-21)
logistic NDL_SLSknee GAD7
sum GAD7 if NDL_SLSknee==0, detail
sum GAD7 if NDL_SLSknee==1, detail
graph box GAD7, over (NDL_SLSknee) // Six outliers
scatter GAD7 Subject, mlabel (Subject)
list Subject NDL_SLSknee GAD7
list Subject NDL_SLSknee GAD7 if GAD7>13
logistic NDL_SLSknee GAD7 if Subject!=45 & Subject!=31 & Subject!=75 & Subject!=111 &
Subject!=121 & Subject!=201
di (.9861077 -.9304718) /.9861077 // A small change of 5% of OR


//No significant change in OR, CI, Std error or P when the outliers are excluded.


histogram GAD7, normal // Skewed to the right and not normally distributed
symplot GAD7 // Skewed to the right
qnorm GAD7, grid //focus on the tails, not ok
pnorm GAD7, grid //Focus on the middle of the distribution, not ok
sktest GAD7 //P<0.05, thus we can reject the hypothesis that GAD/ is normally distributed
tabstat GAD7, stat(n mean sd p25 p50 p75)
di 6.115079-5 //Mean-Median = 1.115079
di 8-5 //Upper quartile-median = 3
di 5-3 //median-lower quartile= 2
```

```
//The variable GAD7 is not normally distributed


logistic NDL_SLSknee GAD7_cat2
logistic NDL_SLSknee i.GAD7_cat2
tab NDL_SLSknee GAD7_cat2
```

***STEP 2: CHECKING FOR COLLINEARITY AND LINEAR ASSUMPTIONS**

```
*Check for collinearity between the continous independent variables; This will be checked in
the stepwise command

*Check for correlation between the dichotomous independent variables;
reg NDL_SI_1 NDL_SI_knee_noknee //reg=0.83
spearman NDL_SI_1 NDL_SI_knee_noknee //spearman=0.66
spearman NDL_SI_1 NDL_SI_knee_noknee  //kap=0.63

//conclusion: There is a high correlation between NDL_SI_1 and NDL_SI_knee_noknee, they should
not //be analysed together in a model. The variable DL_SI_1 is used due to few cell count for
the variable non-dominant leg.

corr NDL_SI_1 NDL_SI_knee_noknee NDL_TLI_4W NDL_IP_4W
spearman NDL_SI_1 NDL_SI_knee_noknee NDL_TLI_4W NDL_IP_4W //non of the other dichotomous
variables are correlated


reg GAD7_cat2 PSQI_cat2    //reg=0.48

corr GAD7_cat2 PSQI_cat2 //corr=0.32
spearman GAD7_cat2 PSQI_cat2 //spearman=0.31
kap GAD7_cat2 PSQI_cat2    //kap=0.15


//No correlation between GAD and PSQI


*Test the assumptions for liniarity for each continuous variable in the model; thus that the
logit increases/decreases linearly
//as a function of the continuous covariates.
logistic NDL_SLSknee i.div Age NDL_ADF NDL_Nm3BWm_100 NDL_SI_1 NDL_TLI_4W NDL_IP_4W AFAQ PSS14
PSQI i.GAD7_cat2

//FIRST, linearity is checked with plots:

logistic NDL_SLSknee i.div Age NDL_ADF NDL_Nm3BWm_100 NDL_SI_1 NDL_TLI_4W NDL_IP_4W AFAQ PSS14
PSQI i.GAD7_cat2
predict logits_NDL, xb

scatter logits_NDL Age || lowess logits_NDL Age //Does not look linear, probably affected by
to outliers
scatter logits_NDL NDL_ADF || lowess logits_NDL NDL_ADF //looks ok
scatter logits_NDL NDL_Nm3BWm_100 || lowess logits_NDL NDL_Nm3BWm_100 //Looks ok
scatter logits_NDL AFAQ || lowess logits_NDL AFAQ //looks ok
scatter logits_NDL PSS14 || lowess logits_NDL PSS14 //looks ok
scatter logits_NDL PSQI || lowess logits_NDL PSQI //Does not look ok

//In order to get linearity for Age and PSQI:
```

```
//Age: Checks if there is any difference when outliers are excluded. No differences, p<0.05.
See which are outliers in step 1.
boxtid logit DL_SLSknee Age_01

//Age: Transformation
gen Age_sqrt= sqrt(Age)
boxtid logit DL_SLSknee Age_sqrt //p=0.011
gen Age_sq=Age^2
boxtid logit DL_SLSknee Age_sq //p=0.0006
gen Age_log=log(Age)
boxtid logit DL_SLSknee Age_log //p=0.015

//Age: Visual check of transformed
logistic NDL_SLSknee i.div Age NDL_ADF NDL_Nm3BWm_100 NDL_SI_1 NDL_TLI_4W NDL_IP_4W AFAQ PSS14
PSQI i.GAD7_cat2 Age_sqrt Age_sq Age_log
predict logits_NDL_Age, xb

scatter logits_NDL_Age Age_sqrt || lowess logits_NDL_Age Age_sqrt //does not look ok
scatter logits_NDL_Age Age_sq || lowess logits_NDL_Age Age_sq //does not look ok
scatter logits_NDL_Age Age_log || lowess logits_NDL_Age Age_log //does not look ok


//PSQI: Transformation
gen PSQI_sqrt= sqrt(PSQI) //Square root transformation in order to get linearity
boxtid logit NDL_SLSknee PSQI_sqrt //p=0.22
gen PSQI_sq=PSQI^2
boxtid logit NDL_SLSknee PSQI_sq //Hessian is not negative semidefinite
gen PSQI_log=log(PSQI)
boxtid logit NDL_SLSknee PSQI_log //p=0.22


//PSQI: Visual check of linearity
logistic NDL_SLSknee i.div Age NDL_ADF NDL_Nm3BWm_100 NDL_SI_1 NDL_TLI_4W NDL_IP_4W AFAQ PSS14
PSQI i.GAD7_cat2 PSQI_sqrt PSQI_sq PSQI_log
predict logits_NDL_PSQI, xb

scatter logits_NDL_PSQI PSQI_log || lowess logits_NDL_PSQI PSQI_log //does not look ok
scatter logits_NDL_PSQI PSQI_sqrt || lowess logits_NDL_PSQI PSQI_sqrt //does not look ok
scatter logits_NDL_PSQI PSQI_sq || lowess logits_NDL_PSQI PSQI_sq ////does not look ok


//SECONDLY, the boxtid test is used to check if the variables fullfills the assumption of
linearity. If P>0.05 the assumption is fulfilled:
//(For transformed values of PSQI and Age see above)
boxtid logit NDL_SLSknee Age NDL_Nm3BWm_100 NDL_ADF AFAQ PSS14 PSQI //Age=0.09, NDL_Nm3BWm_100
P=0.212, NDL_ADF=0.55, AFAQ P=0.52, PSS14=0.68 and PSQI=0.49

//THIRDLY, the linktest is used to test if the model has a reasonable fit and is adequate
logistic NDL_SLSknee i.div Age NDL_ADF NDL_Nm3BWm_100 NDL_SI_1 NDL_TLI_4W NDL_IP_4W AFAQ PSS14
PSQI i.GAD7_cat2
linktest
//The model has a reasonable fit if _hat is significant, the results in this model is p=0.000.
The _hatsq is adequate if P>0.05, this model has p=0.384.
//A significant result for _hatq means that an alternativ binary regression model should be
considered (quadratic function, not using the logit),
//it may also indicate that important predictors have been omitted or are represented
incorrectly in the model
```

```
//Conclusion:Age has a significant Boxtid test and is not linear when visually inspected, the variable is not either //linear when transformed, the variable will be categorized. //The variable PSQI has a n.s Boxtid test but is not linear when visually inspected//or transformed. This variable will be categorized in accordance with a reference with at cut off.
```

***STEP 3: STEPWISE LOGISTIC REGRESSION**

```
//stepwise logistic without excluding outliers: No outliers were found in step 1 that did any significant changes.

//As the variable Age has a low p value and does not look ok at the visual inspection, the continous variable Age
//is compared with the categorised variable.
xi: stepwise, pr(.2)pe(.05): logistic NDL_SLSknee (i.div) Age NDL_ADF NDL_Nm3BWm_100 NDL_SI_1 NDL_TLI_4W NDL_IP_4W AFAQ PSS14 PSQI (i.GAD7_cat2)
estat gof, group(10)
linktest //https://stats.oarc.ucla.edu/stata/webbooks/logistic/chapter3/lesson-3-logistic-regression-diagnostics/

xi: stepwise, pr(.2)pe(.05): logistic NDL_SLSknee (i.div) (i.Age_cat) NDL_ADF NDL_Nm3BWm_100 NDL_SI_1 NDL_TLI_4W NDL_IP_4W AFAQ PSS14 PSQI (i.GAD7_cat2)
estat gof, group(10)
linktest     //there is no difference in the output if the variable Age is categorised or not
```

***STEP 4: CHECK FOR INTERACTION AMONG THE VARIABLES IN THE FINAL MODEL**

```
//At this stage the variable DL_SI_1 is taken away as it is highly correlated to DL_SI_knee_noknee, the latter variable is of greater interest when
//looking at the specifik knee segment
xi: stepwise, pr(.2)pe(.05): logistic NDL_SLSknee (i.div) (i.Age_cat) NDL_ADF NDL_Nm3BWm_100 NDL_SI_1 NDL_TLI_4W NDL_IP_4W AFAQ PSS14 (i.PSQI_cat2) (i.GAD7_cat2)
//included variables are: div, AFAQ, NDL_IP_4W, NDL_Nm3BWm,

//div vs AFAQ
//Already defined: gen div_AFAQ=div* AFAQ
logistic NDL_SLSknee div AFAQ div_AFAQ //p=0.60
//div vs NDL_IP_4W
//Already defined: gen div_NDL_IP_4W=div* NDL_IP_4W
logistic NDL_SLSknee div NDL_IP_4W div_NDL_IP_4W //p=0.53
//div vs NDL_Nm3BWm
//Already defined: gen div_CLAM=div*NDL_Nm3BWm
logistic NDL_SLSknee div NDL_Nm3BWm div_CLAM //p=0.80

//AFAQ vs NDL_IP_4W
//Already defined: gen AFAQ_IP4W= AFAQ* NDL_IP_4W
logistic NDL_SLSknee AFAQ NDL_SI_knee_noknee AFAQ_IP4W //p=0.12
//AFAQ vs NDL_Nm3BWm_100
gen AFAQ_NDL_Nm3BWm_100= AFAQ* NDL_Nm3BWm_100
logistic NDL_SLSknee AFAQ NDL_Nm3BWm_100 AFAQ_NDL_Nm3BWm_100 //p=0.30

//NDL_IP_4W vs NDL_Nm3BWm_100
gen NDL_IP_4W_NDL_Nm3BWm_100= NDL_IP_4W* NDL_Nm3BWm_100
logistic NDL_SLSknee NDL_IP_4W NDL_Nm3BWm_100 NDL_IP_4W_NDL_Nm3BWm_100 //p=0.28

//conclusion: No interaction found
```

***STEP 5: CHECKING FOR MODEL ASSUMPTIONS AND FIT**

```
xi: stepwise, pr(.2)pe(.05): logistic NDL_SLSknee (i.div) (i.Age_cat) NDL_ADF NDL_Nm3BWm_100
NDL_SI_1 NDL_TLI_4W NDL_IP_4W AFAQ PSS14 (i.PSQI_cat2) (i.GAD7_cat2)

*FIRSTLY, Check for collinearity; This will be checked in the stepwise command but could also
be checked like this:
regress NDL_ADF NDL_Nm3BWm_100 AFAQ PSS14
vif // If vif =>5 there is coliniarity. In this test vif=1.04
collin DL_ADF DL_Nm3BWm_100 AFAQ PSS14 //If the variables are non correlated both vif and
tolerance

//should be close to 1. In this test both tolerance and vif is close to 1.0



*SECONDLY, checking for outlying, high leverage and influential points
//Starts by looking at residual and leverage plots
xi: stepwise, pr(.2)pe(.05): logistic NDL_SLSknee (i.div) (i.Age_cat) NDL_ADF NDL_Nm3BWm_100
NDL_SI_1 NDL_TLI_4W NDL_IP_4W AFAQ PSS14 (i.PSQI_cat2) (i.GAD7_cat2)

logistic NDL_SLSknee i.div i.Age_cat NDL_ADF NDL_Nm3BWm_100 NDL_SI_1 NDL_TLI_4W NDL_IP_4W AFAQ
PSS14 i.PSQI_cat2 i.GAD7_cat2
predict phat_NDL, p
predict rstandard_NDL, rstandard
scatter rstandard_NDL phat_NDL, mlabel(Subject) //Produces a plot for the standardized Pearson
residuals: Subject 145, 75, 71, 121 looks like outlierd. //But there is also a lot of other
variables that have a residual >2. Those are not identifiable

scatter rstandard_NDL Subject, mlabel(Subject) //Produces an indexplot for the standardized
Person residuals: Shows large residuals for //subjects 75, 145, 36, 83, 11, 25, 121, 71, 141,
178

predict dv_NDL, dev
scatter dv_NDL phat_NDL, mlab(Subject) //Produces a deviance residual plot, another type of
plot that should give the same results as the Pearson residuals: //not usable

predict hat_NDL, hat
scatter hat_NDL phat_NDL, mlab(Subject) //Produces a leverage plot: Mean levereage is
approximately 0.06. 0.15/0.06=2.5 so values around 0.06 should //be counted as having high
leverage. Subjects 258, 155, 74, 53, 62, 5, 90, 62, 143, 221, 211, has high leverage.
//subjects 75, 145, 36, 83, 11, 25, 121, 71, 141, 178  with large residual has low leverage.


scatter hat_NDL Subject, mlab(Subject) //Produces a leverage indexplot: subjects 75, 145, 36,
83, 11, 25, 121, 71, 141, 178  with large residual has low leverage. ///Subject 258, 155, 74,
5, 53, 62, 90, 143, 221 have high leverage.


clist if Subject==258 | Subject==155 | Subject==74 | Subject==5 | Subject==53 | Subject==62 |
Subject==90 | Subject==143 | Subject==221 | Subject==211

//Looking at the values for all the different variables for subject 258, 155, 74, 5, 53, 62,
90, 143, 221, 211.
//which does not show any flagrant errors

//Comparing the models with and without Subject 258, 155, 74, 5, 53, 62, 90, 143, 221, 211.
```

```
xi: stepwise, pr(.2)pe(.05): logistic NDL_SLSknee (i.div) (i.Age_cat) NDL_ADF NDL_Nm3BWm_100
NDL_SI_1 NDL_TLI_4W NDL_IP_4W AFAQ PSS14 (i.PSQI_cat2) (i.GAD7_cat2)

xi: stepwise, pr(.2)pe(.05): logistic NDL_SLSknee (i.div) (i.Age_cat) NDL_ADF NDL_Nm3BWm_100
NDL_SI_1 NDL_TLI_4W NDL_IP_4W AFAQ PSS14 (i.PSQI_cat2) (i.GAD7_cat2) if Subject!=258
//IPSQI_cat2 is counted into the model but n.s
xi: stepwise, pr(.2)pe(.05): logistic NDL_SLSknee (i.div) (i.Age_cat) NDL_ADF NDL_Nm3BWm_100
NDL_SI_1 NDL_TLI_4W NDL_IP_4W AFAQ PSS14 (i.PSQI_cat2) (i.GAD7_cat2) if Subject!=155
//IPSQI_cat2 and NDL_TLI_4W is counted into the model but n.s
xi: stepwise, pr(.2)pe(.05): logistic NDL_SLSknee (i.div) (i.Age_cat) NDL_ADF NDL_Nm3BWm_100
NDL_SI_1 NDL_TLI_4W NDL_IP_4W AFAQ PSS14 (i.PSQI_cat2) (i.GAD7_cat2) if Subject!=74
xi: stepwise, pr(.2)pe(.05): logistic NDL_SLSknee (i.div) (i.Age_cat) NDL_ADF NDL_Nm3BWm_100
NDL_SI_1 NDL_TLI_4W NDL_IP_4W AFAQ PSS14 (i.PSQI_cat2) (i.GAD7_cat2) if Subject!=5
//NDL_TLI_4W is counted into the model but n.s
xi: stepwise, pr(.2)pe(.05): logistic NDL_SLSknee (i.div) (i.Age_cat) NDL_ADF NDL_Nm3BWm_100
NDL_SI_1 NDL_TLI_4W NDL_IP_4W AFAQ PSS14 (i.PSQI_cat2) (i.GAD7_cat2) if Subject!=53
//NDL_TLI_4W is counted into the model but n.s
xi: stepwise, pr(.2)pe(.05): logistic NDL_SLSknee (i.div) (i.Age_cat) NDL_ADF NDL_Nm3BWm_100
NDL_SI_1 NDL_TLI_4W NDL_IP_4W AFAQ PSS14 (i.PSQI_cat2) (i.GAD7_cat2) if Subject!=62
xi: stepwise, pr(.2)pe(.05): logistic NDL_SLSknee (i.div) (i.Age_cat) NDL_ADF NDL_Nm3BWm_100
NDL_SI_1 NDL_TLI_4W NDL_IP_4W AFAQ PSS14 (i.PSQI_cat2) (i.GAD7_cat2) if Subject!=90
//IPSQI_cat2 is counted into the model but n.s
xi: stepwise, pr(.2)pe(.05): logistic NDL_SLSknee (i.div) (i.Age_cat) NDL_ADF NDL_Nm3BWm_100
NDL_SI_1 NDL_TLI_4W NDL_IP_4W AFAQ PSS14 (i.PSQI_cat2) (i.GAD7_cat2) if Subject!=143
//NDL_TLI_4W is counted into the model but n.s
xi: stepwise, pr(.2)pe(.05): logistic NDL_SLSknee (i.div) (i.Age_cat) NDL_ADF NDL_Nm3BWm_100
NDL_SI_1 NDL_TLI_4W NDL_IP_4W AFAQ PSS14 (i.PSQI_cat2) (i.GAD7_cat2) if Subject!=221
xi: stepwise, pr(.2)pe(.05): logistic NDL_SLSknee (i.div) (i.Age_cat) NDL_ADF NDL_Nm3BWm_100
NDL_SI_1 NDL_TLI_4W NDL_IP_4W AFAQ PSS14 (i.PSQI_cat2) (i.GAD7_cat2) if Subject!=211
//NDL_SI_1 is counted into the model but n.s
xi: stepwise, pr(.2)pe(.05): logistic NDL_SLSknee (i.div) (i.Age_cat) NDL_ADF NDL_Nm3BWm_100
NDL_SI_1 NDL_TLI_4W NDL_IP_4W AFAQ PSS14 (i.PSQI_cat2) (i.GAD7_cat2) if Subject!=258 &
Subject!=155 & Subject!=5 & Subject!=74 & Subject!=53  & Subject!=62 & Subject!=90 &
Subject!=143  & Subject!=221             & Subject!=211
// No greater change seen for the overall conclusion of the model, neighter for direction or
significance (n.s to significance or vice verse) for any specific coefficient.


//No influential points is found, no flagrant error is detected. All subjects stays in the
model



*THIRDLY, Model fit
xi: stepwise, pr(.2)pe(.05): logistic NDL_SLSknee (i.div) (i.Age_cat) NDL_ADF NDL_Nm3BWm_100
NDL_SI_1 NDL_TLI_4W NDL_IP_4W AFAQ PSS14 (i.PSQI_cat2) (i.GAD7_cat2)
estat gof, group(10) table //the gof test is significant p=0.03
//A significant finding indicates lack of fit and a non-significant result rules out a gross
lack of fit. Large p values does not always say that
//you have a good fit, just that there is no evidence enought to say that there is a bad fit.
The test is very sensitive to
//fairly small fit discrepancies in a larger sample. Thus, a significant results in such cases
may not signal a serious fit problem in such cases
linktest //The model has a reasonable fit if _hat is significant, the results in this model is
p=0.001. The model is adequate if _hatsq P>0.05, this model has p=0.50.
//A significant result for _hatq means that an alternativ binary regression model should be
considered (quadratic function, not using the logit),
//it may also indicate that important predictors have been omitted or are represented
incorrectly in the model
```

```
*Check of linearity in final model:
logistic NDL_SLSknee i.div NDL_Nm3BWm_100 NDL_IP_4W AFAQ
predict logits_NDL_final, xb

scatter logits_NDL_final NDL_Nm3BWm_100 || lowess logits_NDL_final NDL_Nm3BWm_100 //Looks a little bit flat in the beginning but this is probably due to the outliers
scatter logits_NDL_final AFAQ || lowess logits_NDL_final AFAQ //looks ok



              //THIS IS THE FINAL MODEL




*********************************************************************************************
*SENSITIVITY ANALYSIS FOR BOTH DOMINANT AND NON DOMINANT LEG
*********************************************************************************************
logistic NDL_SLSknee i.AFAQ_cat3 //A good or at least ok dose response for increased levels of fear of avoidance
logistic DL_SLSknee i.PSS14_cat3 // A poor dose response with increasing levels of stress
```
